# Electrochemical oxidative radical cascade cyclization of dienes and diselenides towards the synthesis of seleno-benzazepines†

**DOI:** 10.1039/d4ra01914h

**Published:** 2024-04-18

**Authors:** Ling Hu, Jingyi Zhang, Minghan Li, Yulin Feng, Fangling Lu

**Affiliations:** a The National Pharmaceutical Engineering Center for Solid Preparation in Chinese Herbal Medicine, Jiangxi University of Chinese Medicine 56 Yangming Road Jiangxi Nanchang 330006 P. R. China fengyulin2003@126.com 20211036@jxutcm.edu.cn

## Abstract

Selenium-containing compounds are important scaffolds owing to their value in medicinal chemistry, biochemistry and material chemistry. Herein, we report an electrochemical approach to access seleno-benzazepines through an oxidative radical cascade cyclization of dienes with diselenides under metal-free, external oxidant-free and base-free conditions. In a simple undivided cell, various dienes and diselenides were suitable for this transformation, generating the desired products in up to 84% yields. This method provides a green and convenient route for the synthesis of valuable selenium-containing seven-membered N-heterocycles from simple starting materials.

## Abstract 2

An electrochemical approach to access seleno-benzazepines through an oxidative radical cascade cyclization of dienes with diselenides under metal-free, external oxidant-free and base-free conditions.

## Graphical abstract

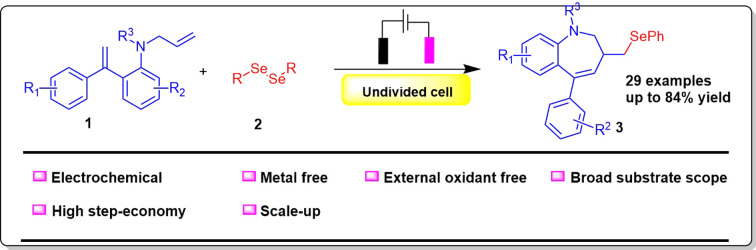

Benzazepine derivatives are a significant family of seven-membered heterocycles with unique bioactive and pharmaceutical properties (Scheme 1).^1–4^ As shown in Scheme 1a, the selected examples of benzazepine medicine exhibit properties such as anti-allergic, anti-depressant, anti-hypertension and diuretic.^5–8^ Therefore, chemists have devoted considerable efforts to develop various ways for the rapid construction of benzazepine derivatives. To date, the benzazepine skeletons can be synthesized by the expansion of smaller rings,^9–11^ Beckmann rearrangements,^12–14^ transition-metal-catalyzed oxidative annulations,^15–22^ radical reactions^23–30^ and others.^31–34^ Among these elegant methods, the radical cascade reaction stands out owing to its high efficiency and simple reaction conditions. For instance, in 2022, Sun and coworkers^29^ reported a K_2_S_2_O_8_/I_2_-promoted electrophilic selenylative cyclization to access seleno-benzo[*b*]azepines (Scheme 1b). Very recently, the same group^30^ developed a visible-light-promoted selective sulfonylation and selenylation of dienes using O_2_ as the terminal oxidant to afford seleno-benzazepines. Despite these advances, developing new methods to prepare selenium-containing seven-membered N-heterocycles under sustainable conditions at an affordable cost is still highly desirable.

**Scheme 1:**
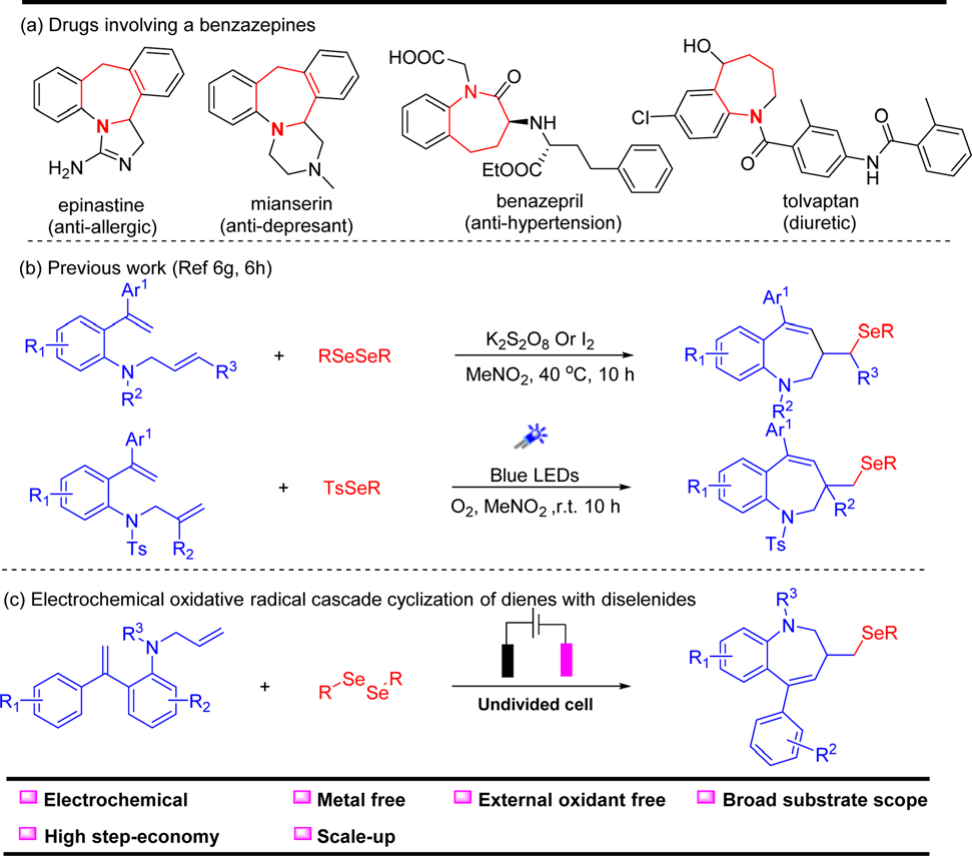
(a) Drugs with benzazepine structures, (b) previous work, (c) electrochemical oxidative selenocyclization of dienes with diselenides.

Over the past few decades, electrosynthesis has offered a green and efficient strategy for redox transformations by employing traceless electrons as redox agents instead of stoichiometric oxidants or reductants.^35–38^ In recent years, some breakthroughs have been made in electrochemical radical cascade selenocyclization to prepare selenium-containing heterocycles.^39–48^ In 2019, Lei and coworkers developed an electrochemical oxidative cyclization of olefinic carbonyls and diselenides towards the synthesis of seleno-dihydrofurans and oxazolines.^39^ Soon after, Sarkar,^40^ Pan,^41^ Xu^42^ and other groups^43–48^ reported a series of related works, respectively. As part of our ongoing focus on radical cascade selenocyclization,^44,45^ herein, we report a facile and efficient method for the selenocyclization of dienes *via* electrochemical synthesis under metal-free and external oxidant-free conditions (Scheme 1c).

We started our studies using *N*-allyl-4-methyl-*N*-(2-(1-phenylvinyl)phenyl)benzenesulfonamide 1a and 1,2-diphenyldiselane 2a as model substrates. After screening the reaction parameters, the seleno-benzazepines 3a was obtained in 80% yield by using ^*n*^Bu_4_NBF_4_ as the electrolyte, CH_3_CN as the solvent, platinum plate as the cathode, graphite rod as the anode, under 18 mA constant current for 6 h in an undivided cell (Table 1, entry 1). The yield decreased significantly when using MeOH as the solvent, and neither DMSO nor DMF was suitable for this transformation (Table 1, entries 2–4). When other supporting electrolytes, such as ^*n*^Bu_4_NPF_6_ and ^*n*^Bu_4_NClO_4_ were used, no better results were achieved (Table 1, entries 5 and 6). The current density had a great influence on the reaction, and increasing or decreasing the electric current resulted in relatively low yields (Table 1, entries 7 and 8). Using a Fe plate or Ni plate instead of a platinum plate as the cathode, the yields dropped to 43% and 41%, respectively (Table 1, entries 9 and 10). The ratio of 2a/1a was also evaluated, and both 3 : 4 and 1 : 2 gave results inferior to 1 : 1 (Table 1, entries 11 and 12). A control experiment indicated that electricity was essential for this reaction (Table 1, entry 11).

**Table 1:** Optimization of the reaction conditions^a^

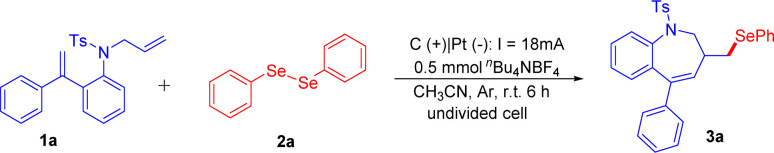		
Entry	Variation from the standard conditions	Yield^b^ (%)
1	None	80
2	MeOH instead of CH_3_CN	16
3	DMF instead of CH_3_CN	n.d.
4	DMSO instead of CH_3_CN	n.d.
5	^*n*^Bu_4_NPF_6_ instead of ^*n*^Bu_4_NBF_4_	63
6	^*n*^Bu_4_NClO_4_ instead of ^*n*^Bu_4_NBF_4_	60
7	12 mA, 9 h	47
8	21 mA, 5 h	68
9	C (+)|Fe (−) instead of C (+)|Pt (−)	43
10	C (+)|Ni (−) instead of C (+)|Pt (−)	41
11	0.5 equiv. 2a	28
12	0.7 equiv. 2a	48
13	No electric current	n.d.

a Reaction conditions: 1a (0.5 mmol), 2a (0.5 mmol), ^*n*^Bu_4_NBF_4_ (0.5 mmol), CH_3_CN (11 mL), graphite rod as the anode (*ϕ* 6 mm, about 15 mm immersion depth in solution) and platinum plate (15 mm × 15 mm × 0.3 mm) as the cathode, undivided cell, 18 mA, Ar, 6 h.

b Isolated yield. n.d. = not detected.

After establishing the optimal reaction conditions, we started to explore the substrate scope of this transformation, and the results are outlined in Scheme 2. Firstly, various substitutions on the aryl group attached to double bonds were tested. The substituents functional groups such as *p*-Me (3b), *m*-Me (3c), and halogens substituents such as *p*-F (3d), *p*-Cl (3e), *p*-Br (3f) were all compatible with this conversion to give the corresponding desired products in 54–84% yields. Subsequently, we turned our attention to exploring the substrate scope of substituents on the benzene ring in arylamines. A series of substituents on the arene were also compatible under the standard reaction conditions, leading to corresponding products in 45–78% yields (3g–3m). We also evaluated the reactivity of internal alkene, and the corresponding product 3n was produced in a 70% yield. Inspired by the above results, we examined the effect of the substitution pattern on the nitrogen atom of substrates. Alkyl sulfonamides were shown to be good candidates for this transformation (3o, 3p). Moreover, various substituents on their benzene rings (*p*-OMe, *p*-^*t*^Bu, *p*-F, *p*-Cl, *p*-Br, *p*-CF_3_, 3, 5-difluoro) were evaluated and shown to be good candidates for this transformation (3q–3v). To our delight, naphthalene-2-sulfonamide was also compatible and afforded the corresponding benzazepines in 78% yield (3w). To further exhibit the reaction generality, a variety of diselenides, including alkyl diselenides and aryl diselenides, were tested to couple with 1a, afforded the desired products in moderate yields (3x–3zc). Unfortunately, the diphenyl ditelluride (3zd) and a substituent on the terminal carbon of the *N*-allyl part (3ze, 3zf) were not tolerated in the standard reaction conditions.

**Scheme 2:**
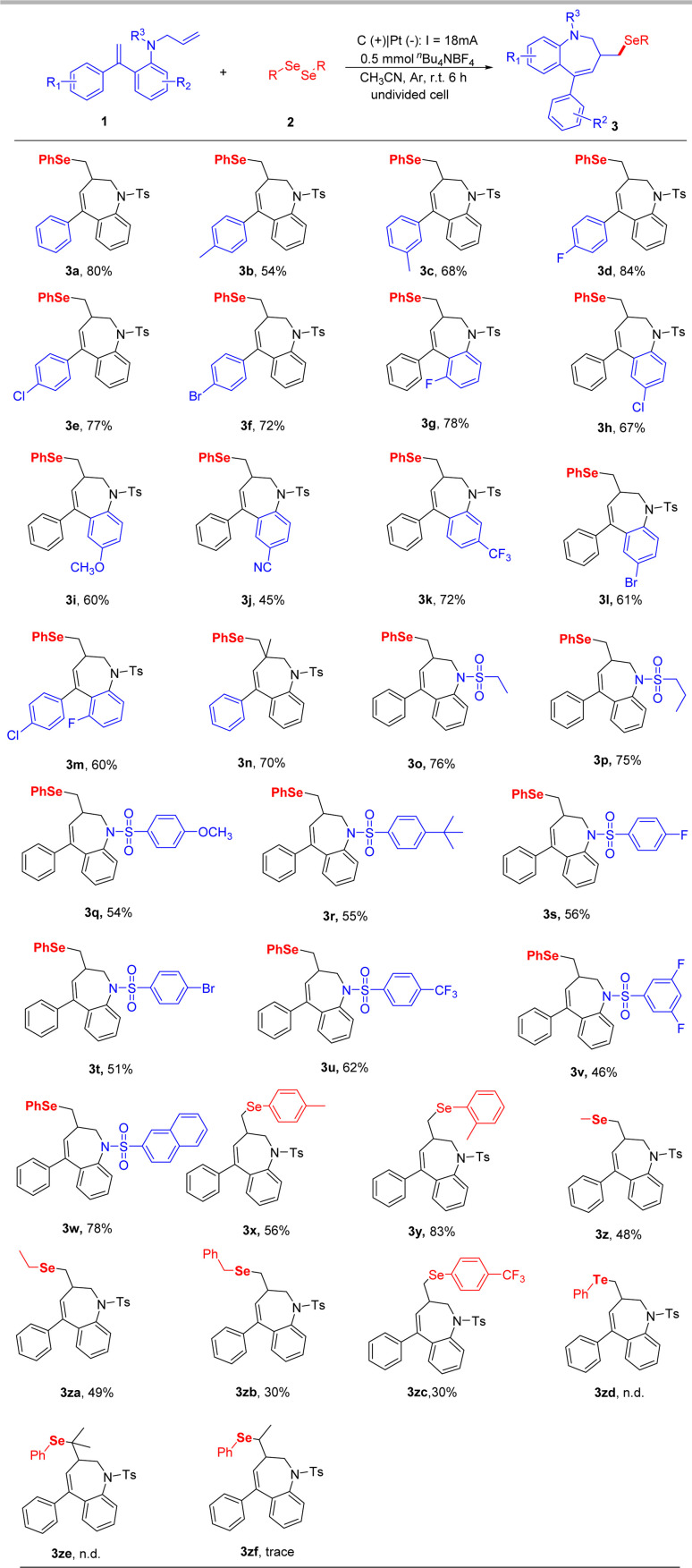
^a^ Reaction conditions: 1a (0.5 mmol), 2a (0.5 mmol), ^*n*^Bu_4_NBF_4_ (0.5 mmol), CH_3_CN (11 mL), graphite rod as the anode (*ϕ* 6 mm, about 15 mm immersion depth in solution) and platinum plate (15 mm × 15 mm × 0.3 mm) as the cathode, undivided cell, 18 mA, Ar, 6 h. ^*b*^ isolated yield. n.d. = not detected.

To verify the practicability of this protocol, the scalability of this electrochemical oxidative radical cascade cyclization of dienes with diselenides was evaluated by performing a 5.0 mmol scale reaction. The reaction of 1a and 2a afforded the desired product 3a in 55% yield (Scheme 3). Besides, the desired product 3a could be transformed into non-selenated heterocycles 4a through a simple treatment, which showed the application potential of this protocol.

**Scheme 3:**
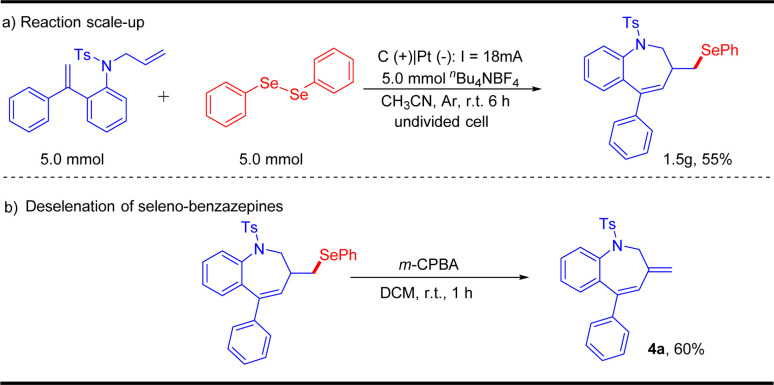
Gram-scale synthesis and derivatizations.

To gain insight into the mechanism of this electrochemical oxidative radical cascade cyclization reaction, related control experiments (Scheme 4) and cyclic voltammetry (CV) experiments (Scheme 5) were carried out. First, radical trapping experiments were conducted. The reaction was completely suppressed when 2.0 equiv. radical scavenger 2,2,6,6-tetramethyl-1-piperidinyloxy (TEMPO) was added. The adduct 5a was detected by LC-MS in the reaction mixture when 2.0 equiv. 1,1-diphenylethylene was added. These results indicated that this cascade cyclization reaction probably underwent a radical pathway, and selenium radical intermediate might be involved in this transformation. Furthermore, cyclic voltammetry (CV) experiments of *N*-allyl-4-methyl-*N*-(2-(1- phenylvinyl)phenyl) benzenesulfonamide 1a and 1,2-diphenyldiselane 2a were performed, respectively. An obvious oxidation peak of 1a was observed at 2.49 V, whereas the oxidation peak of 2a was observed at 1.98 V (see ESI† for details). This result indicated that 2a was oxidized preferentially at the anode in this system.

**Scheme 4:**
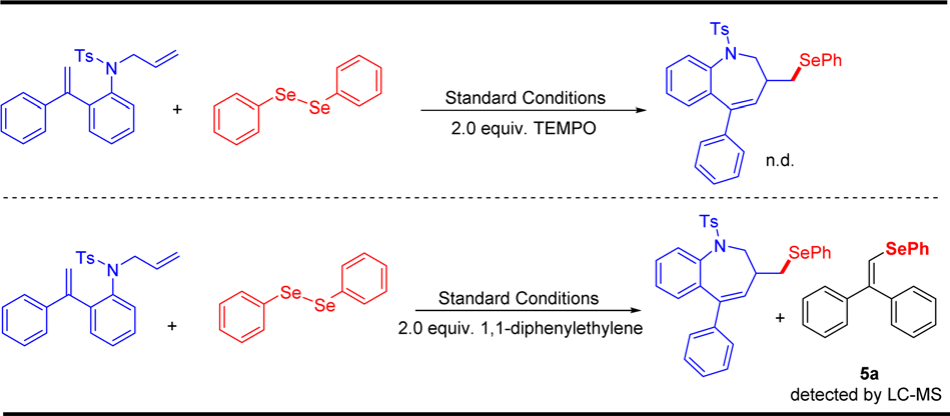
Control experiments.

**Scheme 5:**
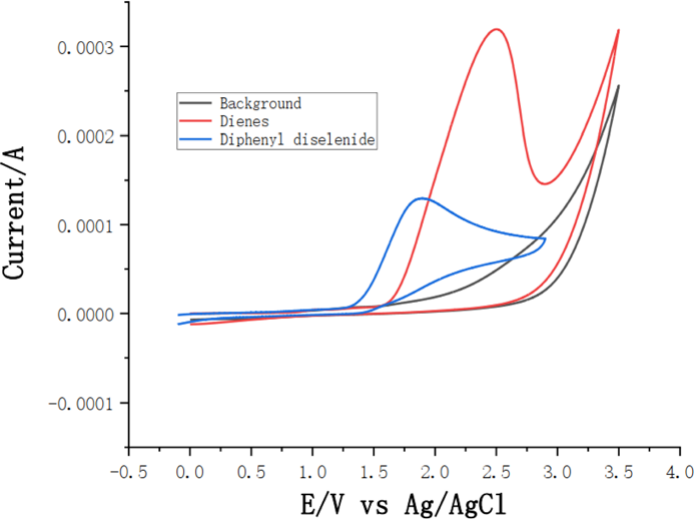
Cyclic voltammetry.

Based on the experimental results above (Scheme 4) and the previous reports,^46^ a possible reaction mechanism was proposed in Scheme 5. Initially, diphenyl selenide was oxidized at the anode to generate a radical cation intermediate A, which divided into phenyl-selenium radical B and phenyl-selenium cation C, respectively. Phenyl-selenium cation C was reduced to diphenyl-selenide at the cathode for the next cycle. Then, the phenyl-selenium radical B added to the C=C double bond of diene to generate the alkyl radical D. Subsequently, the intermediate D underwent radical cyclization to provide the intermediate E. Finally, the intermediate E was further oxidized at the anode and then deprotonated to afford the desired seleno-benzazepines 3a. At the cathode, the proton was reduced to give hydrogen gas during the reaction (Scheme 6).

**Scheme 6:**
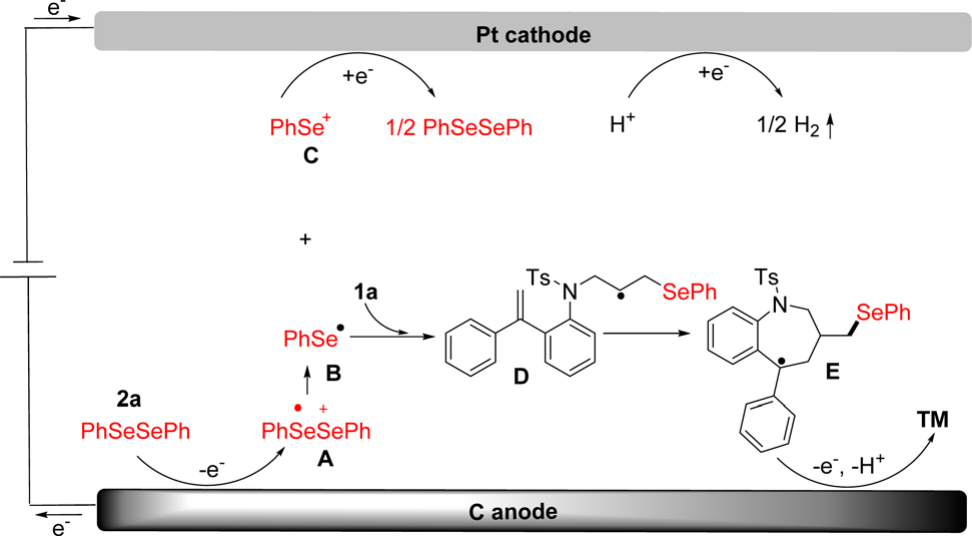
Proposed reaction mechanism.

In summary, a general and efficient electrochemical oxidative radical cascade cyclization of dienes and diselenides has been successfully achieved. Using this eco-friendly method, we were able to synthesize the seleno-benzazepines under metal-free, external oxidant-free and additive-free conditions. Preliminary mechanistic studies indicated that this reaction underwent a radical pathway, and selenium radical intermediate might be involved in this transformation. Further applications of electrochemical oxidative radical cascade cyclization of dienes are currently underway in our group.

## Conflicts of interest

There are no conflicts to declare.

## Supplementary Material

RA-014-D4RA01914H-s001
