# Supplementary material for: Electrochemical oxidative radical cascade cyclization of dienes and diselenides towards the synthesis of seleno-benzazepines
Source: RSC Adv. 2024 Apr 18;14(18):12556–60. doi: 10.1039/d4ra01914h (PMC11024674; doi:10.1039/d4ra01914h)

Electronic Supplementary Material (ESI) for RSC Advances.
This journal is © The Royal Society of Chemistry 2024

Supporting Information

Electrochemical Oxidative Radical Cascade Cyclization of Dienes and Diselenides towards the Synthesis of Seleno-benzazepines

Ling Hu[a], Jingyi Zhang[a], Minghan Li[a], Fangling Lu[*a], Yulin Feng*[a]

[a]The National Pharmaceutical Engineering Center for Solid Preparation in Chinese Herbal Medicine, Jiangxi University of Chinese Medicine, 56 Yangming Road, Jiangxi, Nanchang 330006, P. R. China.

Table of Contents

General information………………………………………………….. S3

Experimental procedure……………………………………………S4

Mechanism research………………………………………………..S6

Detail descriptions for products……………………………………S8

References…………………………………………….…….….….. S19

Copies of ^{1}H NMR, ^{13}C NMR and ^{19}F NMR spectra……………..... S20

General information

Unless otherwise noted, materials were obtained from commercial suppliers and used without further purification. The instrument for electrolysis was dual display potentiostat (DJS-292B) (made in China). The anodic electrode was graphite rod (ϕ 6 mm) and cathodic electrode was platinum plate (15 mm×15 mm×0.3 mm). Thin layer chromatography (TLC) employed glass 0.25 mm silica gel plates. Flash chromatography columns were packed with 300-400 mesh silica gel in petroleum (boiling point was between 60-90 ℃). Gradient flash chromatography was conducted eluting with a continuous gradient from petroleum to the indicated solvent, and they were listed as volume/volume ratios. NMR spectra were recorded on a Bruker spectrometer at 600 MHz (^{1}H NMR), 151 MHz (^{13}C NMR), 565 MHz (^{19}F NMR) or 400 MHz (^{1}H NMR), 100 MHz (^{13}C NMR). Chemical shifts were reported relative to tetramethylsilane, dimethyl sulfoxide (2.50 ppm for ^{1}H, 39.6 ppm for ^{13}C), respectively. And all ^{1}H, ^{13}C and ^{19}F NMR data spectra were reported in delta (δ) units, parts per million (ppm) downfield from the internal standard. Coupling constants were reported in Hertz (Hz). LC-MS spectra were recorded on a AB SCIEX TripleTOF 5600^{+}.

Experimental procedure

General procedure for the preparation of 3:

In an oven-dried undivided three-necked bottle (25 mL) equipped with a stir bar, dienes **1** (0.5 mmol), diselenides **2** (0.5 mmol), ^{n}Bu$_4$NBF$_4$ (0.5 mmol, 164.6 mg), CH_3CN (11 mL) was added. The bottle was equipped with graphite rod (ϕ 6 mm, about 15 mm immersion depth in solution) as the anode and platinum plate (15 mm×15 mm×0.3 mm) as the cathode. The reaction mixture was stirred and electrolyzed at a constant current of 18 mA under Ar atmosphere at room temperature for 6 h. After completion of the reaction, as indicated by TLC and LC-MS, the crude mixture product was obtained by flash column chromatography on silica gel (petroleum ether: ethyl acetate = 100: 1).

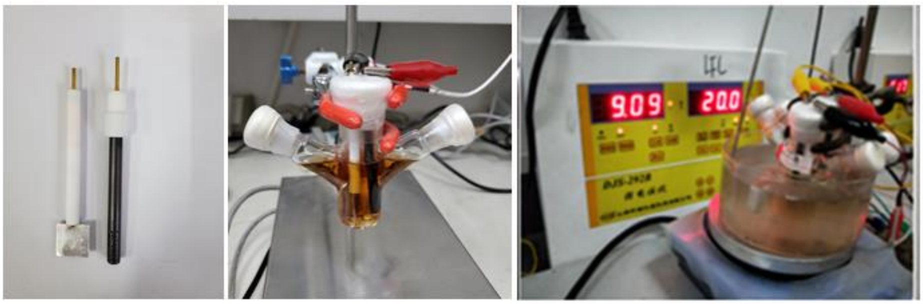

A **B** **C**

Figure **S1**. The experimental setup for electrolysis. (**A**: The electrodes used in the reaction. **B** and **C**: The electrochemical reaction apparatus used.)

Procedure for gram scale synthesis of 3a:

In an oven-dried undivided beaker (100 mL) equipped with a stir bar, *N*-allyl-4-methyl-*N*-(2-(1-phenylvinyl)phenyl)benzenesulfonamide **1a** (5.0 mmol, 1.95 g), 1,2-diphenyldiselane **2a** (5.0 mmol, 1.56 g), ^{n}Bu$_4$NBF$_4$ (5.0 mmol, 1.65 g), CH_3CN (100 mL) was added. The bottle was equipped with graphite rod (ϕ 6 mm, about 15 mm immersion depth in solution) as the anode and platinum plate (15 mm×15 mm×0.3 mm) as the cathode. The reaction mixture was stirred and electrolyzed at a constant current of 18 mA under Ar atmosphere at room temperature for 48 h, after completion of the reaction, as indicated by TLC and LC-MS. The pure product **3a** (yield 55%, white solid, 1.52 g) was obtained by flash column chromatography on silica gel (petroleum ether: ethyl acetate = 100:1).

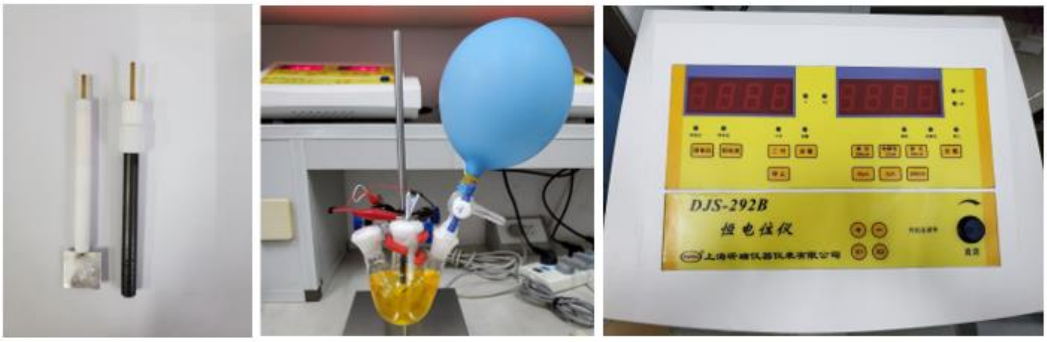

A **B** **C**

Figure **S2**. The experimental setup for electrolysis. (**A**: The electrodes used in the reaction. **B** and **C**: The electrochemical reaction apparatus used.)

General procedure for the preparation of 1[1-3]:

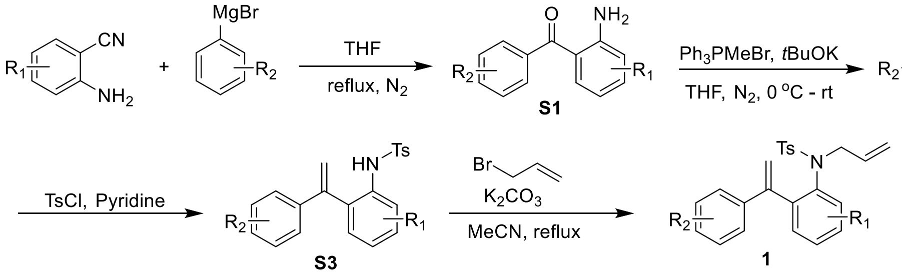

Step1: To a round-bottomed flask charged with the 2-aminobenzonitrile (10 mmol, 1.0 equiv.) in dry THF (20 mL), arylmagnesium bromide (30 mmol, 3.0 equiv., 2.8 M in THF) was added dropwise via syringe at 0 °C in a nitrogen atmosphere. The reaction mixture was heated to oil bath 65 °C for 10 h. Upon the reaction completed, the suspension was cooled to 0 °C, HCl solution (6 M) was added dropwise and mixture was stirred atroom temperature for another 12 h. Then, the mixture was basified to pH = 9 with 10% NaOH solution, extracted with ethyl acetate. The separated organic layer was washed with brine, dried over anhydrous Na_2SO_4, filtered, and concentrated in vacuo. The residue was purified by silica gel column chromatography (eluent: petroleum ether: ethyl acetate = 40:1) to afford **S1**.

Step2: To a round-bottom flask of tetrahydrofuran (20 mL) solution containing Ph_3PMeBr (10.4 mmol, 1.3 equiv.), potassium tert-butanol (13.6 mmol, 1.7 equiv.) was added in batches at 0 ℃ in a nitrogen atmosphere. **S1** (8.0 mmol, 1.0 equiv.) was added dropwise at 0 °C. The reaction mixture was warmed to room temperature and stirred for overnight under N_2. Upon the reaction completed, the desired product **S2** was purified on silica gel column by flash column chromatography (eluent: petroleum ether: ethyl acetate = 100:1).

Step3: To a round-bottomed flask with TsCl (13.2 mmol, 1.1 equiv.), product **S2** (12.0 mmol, 1.0 equiv.) was added and then pyridine (30 mL) was dropped at 0 °C. The reaction mixture was warmed to room temperature and stirred for 12 h. Upon the reaction completed, the reaction mixture was extracted with ethyl acetate. The separated organic layer was washed with brine, dried over anhydrous Na_2SO_4, filtered, and concentrated in vacuo. The residue was purified by silica gel column chromatography (petroleum ether: ethyl acetate = 80:1) to afford **S3**.

Step4: To a round bottom flask containing potassium carbonate (3.6 mmol, 3.0 equiv.), 4-Bromo-1-butene (17.3 mmol, 3.4 equiv.), **S3** (5.0 mmol, 1.0 equiv) and acetonitrile (40 mL) were added. The reaction mixture was stirred in oil bath at 85 °C for 12 h. Upon the reaction completed, the reaction mixture was concentrated in vacuo. The residue was purified by silica gel column chromatography (eluent: petroleum ether: ethyl acetate = 100:1) to afford **1**.

Mechanism research

In order to investigate the possible mechanism of this transformation, a series control experiments were carried out. When 2.0 equivalent radical scavenger 2,2,6,6-tetramethylpiperidin-1-oxyl (TEMPO) was added, no selenocyclization product as obtained. When 2.0 equiv. 1,1-diphenylethene (DPE) was added, the DPE-SePh adduct was detected by LC-MS in the reaction system (Figure **S3**). These results indicated that this electrochemical oxidative selenocyclization reaction probably underwent a radical pathway, and selenium radical intermediate might be involved in this transformation.

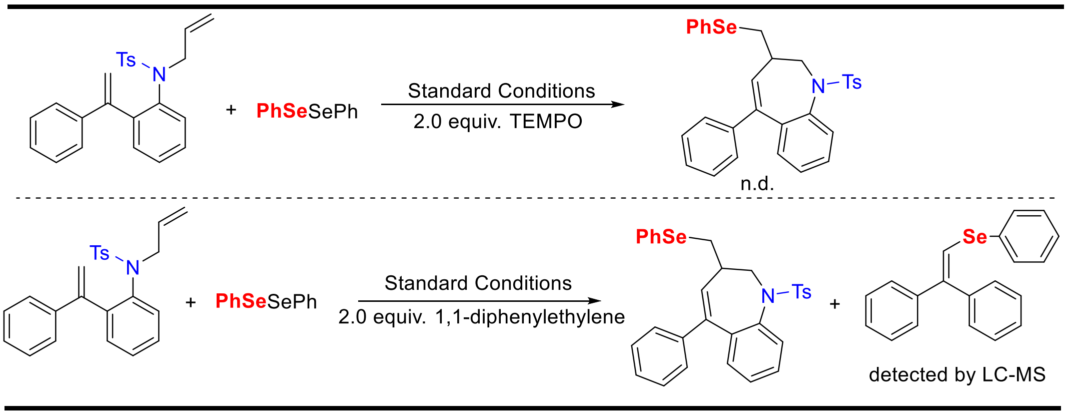

Scheme 1. Control experiments.

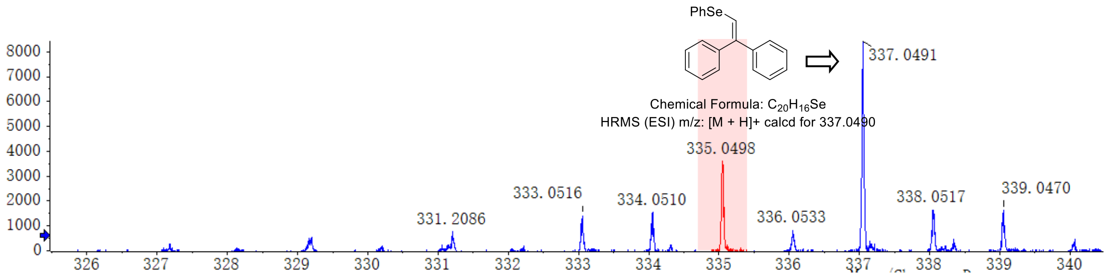

Figure **S3.** Free radical capture experiment (DPE-SePh)

General procedure for cyclic voltammetry (CV):

Cyclic voltammetry was performed in a three-electrode cell connected to a schlenk line under air at room temperature. The working electrode was a glassy carbon electrode, the counter electrode a platinum wire. The reference was an Ag/AgCl electrode submerged in saturated aqueous KCl solution. 10 mL of CH_3CN containing 0.01 M ^{n}Bu$_4$NBF$_4$ were poured into the electrochemical cell in all experiments. The scan rate is 0.1 V/s, ranging from 0 V to 3.5 V. The peak potentials *vs*. Ag/AgCl for used. Cyclic voltammetry (CV) experiments of dienes **1a** and 1,2-diphenyldiselane **2a** were performed. An oxidation peak of **1a** was observed at 2.49 V, while the oxidation peak of **2a** was observed at 1.98 V. This result indicated that **2a** was oxidized preferentially at the anode in this system.

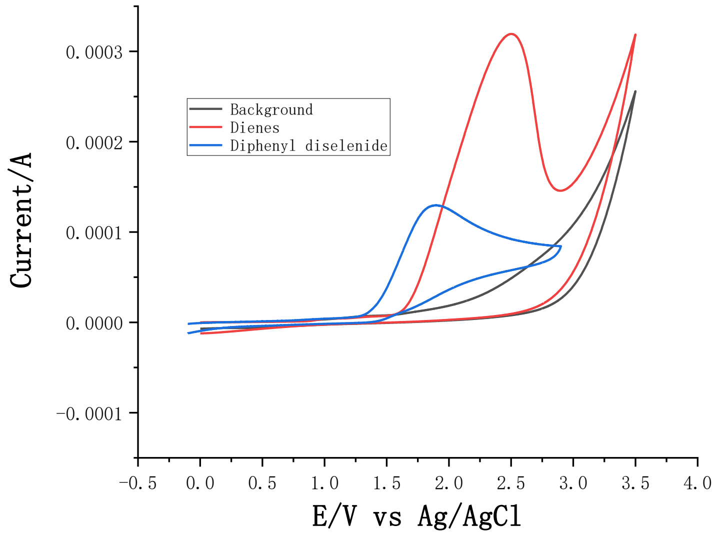

Figure **S4** Cyclic voltammetry

Detail descriptions for products

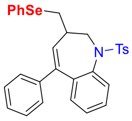

5-phenyl-3-((phenylselanyl)methyl)-1-tosyl-2,3-dihydro-1H-benzo[b]azepine (3a).[1] (White solid was obtained in 80% isolated yield, 217.8 mg). ^{1}H NMR (400 MHz, $CDCl_3$) δ 7.54 (d, 1H, *J* = 7.9 Hz), 7.48-7.44 (m, 4H), 7.26 (t, 1H, *J* = 8.5 Hz), 7.21-7.17 (m, 5H), 7.14 (t, 2H, *J* = 7.3 Hz), 6.89 (d, 3H, *J* = 7.8 Hz), 6.83 (d, 2H, *J* = 7.5 Hz), 5.99 (s, 1H), 4.35 (s, 1H), 4.05 (s, 1H), 3.01-2.91 (m, 2H), 2.74 (s, 1H), 2.16 (s, 3H). ^{13}C NMR (101 MHz, $CDCl_3$) δ 142.99, 141.75, 139.90, 137.83, 133.31, 131.26, 130.57, 129.88, 129.36, 129.30, 128.44, 128.14, 127.97, 127.91, 127.50, 127.41, 62.60, 37.91, 29.89, 21.46.

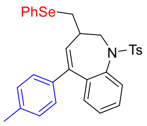

3-((phenylselanyl)methyl)-5-(p-tolyl)-1-tosyl-2,3-dihydro-1H-benzo[b]azepine (3b).[1] (White solid was obtained in 54% isolated yield, 150.8 mg). ^{1}H NMR (600 MHz, DMSO-d_6) δ 7.48-7.44 (m, 4H), 7.38 (d, 1H, *J* = 7.9 Hz), 7.33 (t, 1H, *J* = 6.8 Hz), 7.28-7.24 (m, 4H), 7.04 (d, 2H, *J* = 7.6 Hz), 7.00 (d, 2H, *J* = 7.9 Hz), 6.85 (s, 1H), 6.66 (d, 2H, *J* = 7.7 Hz), 6.03 (s, 1H), 4.38 (s, 1H), 3.99 (s, 1H), 3.15 (d, 2H, *J* = 6.3 Hz), 2.51 (s, 1H), 2.28 (s, 3H), 2.16 (s, 3H). ^{13}C NMR (151 MHz, DMSO-d_6) δ 143.28, 140.85, 140.16, 138.02, 137.12, 132.31, 131.37, 131.31, 130.28, 129.85, 129.74, 128.94, 128.77, 128.66, 127.66, 127.30, 63.31, 38.66, 28.68, 21.30, 21.15.

HRMS (ESI) *m/z*: $[M + H]^+$ calcd for $C_{31}H_{30}NO_2SSe^+$, 560.1157; found, 560.1151.

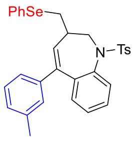

3-((phenylselanyl)methyl)-5-(m-tolyl)-1-tosyl-2,3-dihydro-1H-benzo[b]azepine (3c).[1] (White solid was obtained in 68% isolated yield, 189.9 mg). ^{1}H NMR (600 MHz, DMSO-d_6) δ 7.47-7.45 (m, 4H), 7.39 (d, 1H, *J* = 7.3 Hz), 7.31 (t, 1H, *J* = 7.7 Hz), 7.27-7.23 (m, 4H), 7.08-7.03 (m, 4H), 6.83 (s, 1H), 6.55 (d, 2H, *J* = 13.3 Hz), 6.04 (s, 1H), 4.39 (s, 1H), 3.98 (s, 1H), 3.13 (d, 2H, *J* = 5.9Hz), 2.51 (s, 1H), 2.19 (s, 3H), 2.14 (s, 3H). ^{13}C NMR (151 MHz, DMSO) δ 143.23, 141.08, 140.08, 139.51, 138.04, 137.73, 137.33, 133.35, 132.34, 131.29, 130.28, 129.79, 129.74, 128.80,

128.68, 128.53, 128.23, 127.33, 127.11, 126.32, 125.05, 64.03, 38.82, 28.65, 21.46, 21.29.

HRMS (ESI) *m/z*: $[M + H]^+$ calcd for $C_{31}H_{30}NO_2SSe^+$, 560.1157; found, 560.1147.

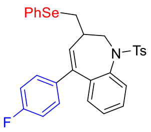

5-(4-fluorophenyl)-3-((phenylselanyl)methyl)-1-tosyl-2,3-dihydro-1H-benzo[b]azepine (3d).[1] (White solid was obtained in 84% isolated yield, 236.3 mg). ^{1}H NMR (600 MHz, DMSO-d_6) δ 7.47-7.44 (m, 4H), 7.39 (d, 1H, *J* = 7.9 Hz), 7.34 (t, 1H, *J* = 7.3 Hz), 7.31-7.24 (m, 4H), 7.06-7.01 (m, 4H), 6.84 (s, 1H), 6.78 (s, 2H), 6.05 (s, 1H), 4.41 (s, 1H), 4.00 (s, 1H), 3.14 (d, 2H, *J* = 11.1 Hz), 2.51 (s, 1H), 2.17 (s, 3H). ^{13}C NMR (151 MHz, DMSO-d_6) δ 162.08 (d, *J* = 244.6 Hz), 143.28, 139.83, 138.05, 137.66, 135.97, 132.37, 132.18, 131.60, 130.19, 129.88, 129.75, 129.62, 128.99, 128.85, 127.33 (d, *J* = 6.0 Hz), 115.15 (d, *J* = 21.1), 64.44, 38.12, 28.61, 21.26. ^{19}F NMR (565 MHz, DMSO) δ -114.95.

HRMS (ESI) *m/z*: $[M + H]^+$ calcd for $C_{30}H_{27}FNO_2SSe^+$, 564.0906; found, 564.0901.

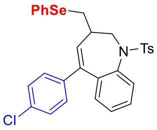

5-(4-chlorophenyl)-3-((phenylselanyl)methyl)-1-tosyl-2,3-dihydro-1H-benzo[b]azepine (3e).[1] (White solid was obtained in 77% isolated yield, 222.9 mg). ^{1}H NMR (600 MHz, DMSO-d_6) δ 7.47-7.40 (m, 5H), 7.37-7.30 (m, 2H), 7.26-7.23 (m, 5H), 7.02 (d, 2H, *J* = 7.8Hz), 6.84 (d, 1H, *J* = 7.2 Hz), 6.75 (d, 2H, *J* = 8.3 Hz) , 6.12 (s, 1H), 4.43 (s, 1H), 4.01 (s, 1H), 3.15 (s, 2H), 2.51 (s, 1H), 2.14 (s, 3H). ^{13}C NMR (151 MHz, DMSO-d_6) δ 143.26, 139.64, 139.46, 138.01, 137.61, 132.54, 132.40, 131.87, 130.17, 129.84, 129.75, 129.23, 129.09, 128.92, 128.34, 127.37, 127.32, 63.27, 38.81, 28.54, 21.25.

HRMS (ESI) *m/z*: $[M + H]^+$ calcd for $C_{30}H_{27}ClNO_2SSe^+$, 580.0611; found, 580.060.

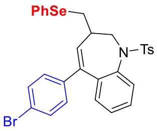

5-(4-bromophenyl)-3-((phenylselanyl)methyl)-1-tosyl-2,3-dihydro-1H-benzo[b]azepine (3f).[1] (White solid was obtained in 72% isolated yield, 224.8 mg). ^{1}H NMR (600 MHz, DMSO-d_6) δ 7.47-

7.46 (m, 2H), 7.43-7.35 (m 6H), 7.32-7.31 (m, 1H), 7.28-7.23 (m, 3H), 7.02 (d, 2H, *J* = 7.9 Hz), 6.85 (s, 1H), 6.67 (d, 2H, *J* = 8.1 Hz), 6.12 (s, 1H), 4.42 (s, 1H), 4.01 (s, 1H), 3.16 (s, 2H), 2.51 (s, 1H), 2.15 (s, 3H). ^{13}C NMR (151 MHz, DMSO-d_6) δ 143.27, 139.56, 138.01, 137.58, 137.18, 136.42, 132.41, 131.27, 130.15, 129.84, 129.76, 129.54, 129.10, 128.92, 127.38, 127.32, 123.21, 121.12, 65.66, 38.60, 28.52, 20.84.

HRMS (ESI) *m/z*: [M + H]$^+$ calcd for $C_{30}H_{27}BrNO_2SSe^+$, 624.0106; found, 624.0106.

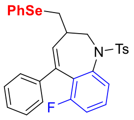

6-fluoro-5-phenyl-3-((phenylselanyl)methyl)-1-tosyl-2,3-dihydro-1H-benzo[b]azepine (3g).[1] (White solid was obtained in 78% isolated yield, 218.2 mg). ^{1}H NMR (600 MHz, DMSO-d_6) δ 7.48 (d, 4H, *J* = 8.8 Hz), 7.46-7.42 (m, 1H), 7.28-7.26 (m, 3H), 7.24-7.18 (m, 5H), 7.04 (d, 2H, *J* = 7.9 Hz), 6.82 (d, 2H, *J* = 7.1 Hz), 6.25 (d, 1H, *J* = 6.4 Hz), 4.32 (t, 1H, *J* = 12.2 Hz), 3.92-3.89 (m, 1H), 3.17 (d, 2H, *J* = 6.6 Hz), 2.57-2.51 (m, 1H), 2.18 (s, 3H). ^{13}C NMR (151 MHz, DMSO) δ 159.19 (d, *J* = 250.7 Hz), 143.50, 139.33, 138.79, 137.89, 136.31, 133.69, 132.66, 130.38 (d, *J* = 10.6 Hz), 129.96, 129.72, 128.39, 127.69, 127.47, 127.24, 127.08, 126.99, 125.96, 116.63 (d, *J* = 21.1 Hz), 60.93, 38.65, 28.31, 19.18. ^{19}F NMR (565 MHz, DMSO) δ -106.12.

HRMS (ESI) *m/z*: [M + H]$^+$ calcd for $C_{30}H_{27}FNO_2SSe^+$, 564.0906; found, 564.0896.

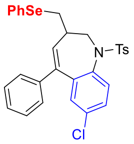

7-chloro-5-phenyl-3-((phenylselanyl)methyl)-1-tosyl-2,3-dihydro-1H-benzo[b]azepine (3h).[1] (White solid was obtained in 67% isolated yield, 193.3 mg). ^{1}H NMR (600 MHz, DMSO-d_6) δ 7.48-7.46 (m, 4H), 7.41 (s, 2H), 7.26-7.24 (m, 4H),7.23-7.20 (m, 2H), 7.06 (d, 2H, *J* = 7.9 Hz), 6.79-6.74 (m, 3H), 6.13 (s, 1H), 4.38 (s, 1H), 3.99 (s, 1H), 3.15 (d, 2H, *J* = 6.4 Hz), 2.51 (s, 1H), 2.16 (s, 3H). ^{13}C NMR (151 MHz, DMSO-d_6) δ143.54, 141.98, 139.97, 139.04, 137.71, 136.68, 133.29, 132.49, 131.47, 130.11, 130.01, 129.73, 129.38, 128.81, 128.53, 128.08, 127.77, 127.37, 127.32, 63.17, 28.74, 28.52, 21.34.

HRMS (ESI) *m/z*: [M + H]$^+$ calcd for $C_{30}H_{27}ClNO_2SSe^+$, 580.0611; found, 580.0608.

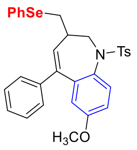

7-methoxy-5-phenyl-3-((phenylselanyl)methyl)-1-tosyl-2,3-dihydro-1H-benzo[b]azepine (3i).[1] (White solid was obtained in 60% isolated yield, 172.4 mg). ^{1}H NMR (600 MHz, DMSO-d_6) δ 7.49 – 7.47 (m, 2H), 7.45 (d, 2H, J = 7.9 Hz), 7.32 (d, 1H, J = 9.0 Hz), 7.28 – 7.23 (m, 4H), 7.21 – 7.18 (m, 2H), 7.02 (d, 2H, J = 7.8 Hz), 6.93 (dd, 1H, J = 8.8, 3.0 Hz), 6.80 (d, 2H, J = 7.6 Hz), 6.32 (s, 1H), 6.08 (s, 1H), 4.38 (s, 1H), 3.98 (s, 1H), 3.62 (s, 3H), 3.16 (s, 2H), 2.54 (s, 1H), 2.15 (s, 3H). ^{13}C NMR (151 MHz, DMSO) δ 158.66, 143.76, 141.25, 141.06, 139.35, 138.08, 132.67, 132.31, 131.73, 130.32, 129.84, 129.74, 128.32, 127.86, 127.76, 127.32, 127.29, 114.84, 114.14, 63.33, 56.69, 36.36, 28.10, 20.55.

HRMS (ESI) *m/z*: $[M + H]^+$ calcd for $C_{31}H_{30}NO_3SSe^+$, 576.1106; found, 576.1111.

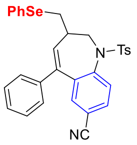

5-phenyl-3-((phenylselanyl)methyl)-1-tosyl-2,3-dihydro-1H-benzo[b]azepine-7-carbonitrile (3j). (White solid was obtained in 45% isolated yield, 128.2 mg). ^{1}H NMR (600 MHz, DMSO-d_6) δ 7.83 (dd, 1H, J = 8.3, 2.0 Hz), 7.60 (d, 1H, J = 8.3 Hz), 7.48 (d, 4H, J = 7.98 Hz), 7.29 – 7.23 (m, 7H), 7.09 (d, 2H, J = 7.9 Hz), 6.76 (d, 2H, J = 7.08 Hz), 6.16 (s, 1H), 4.31 (s, 1H), 4.07 (s, 1H), 3.16 (d, 2H, J = 6.6 Hz), 2.51 (s, 1H), 2.18 (s, 3H). ^{13}C NMR (151 MHz, DMSO-d_6) δ 143.82, 142.34, 139.11, 137.46, 134.14, 132.54, 132.41, 130.13, 130.03, 129.75, 128.65, 128.19, 127.79, 127.40, 127.30, 118.34, 111.57, 62.20, 38.62, 28.42, 20.34.

HRMS (ESI) *m/z*: $[M + H]^+$ calcd for $C_{30}H_{27}N_2O_2SSe^+$, 571.0953; found, 571.0951.

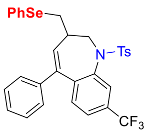

5-phenyl-3-((phenylselanyl)methyl)-1-tosyl-8-(trifluoromethyl)-2,3-dihydro-1H-benzo[b]azepine (3k). (White solid was obtained in 72% isolated yield, 220.5 mg). ^{1}H NMR (600 MHz, DMSO-d_6) δ 7.70 – 7.67 (m, 2H), 7.50 – 7.47 (m, 4H), 7.29 – 7.22 (m, 6H), 7.08 (d, 3H, J = 7.9 Hz), 6.75 (d, 2H, J = 7.5 Hz), 6.19 (s, 1H), 4.41 (s, 1H), 4.10 (s, 1H), 3.21-3.51 (m, 2H), 2.51

(s, 1H), 2.17 (s, 3H).^{13}C NMR (151 MHz, DMSO) δ 145.00, 143.73, 139.72, 138.60, 137.53, 133.64, 132.53, 131.57, 130.09, 130.05, 129.73, 128.92 (q, *J* =16.61 Hz), 128.53, 128.10, 127.82, 127.39, 127.34, 125.40, 124.05 (q, *J* =273.31 Hz), 63.82, 33.11, 28.49, 21.31.^{19}F NMR (565 MHz, DMSO) δ -60.59.

HRMS (ESI) *m/z*: [M + H]$^+$ calcd for $C_{31}H_{27}F_3NO_2SSe^+$, 614.0874; found, 614.0876.

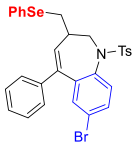

7-bromo-5-phenyl-3-((phenylselanyl)methyl)-1-tosyl-2,3-dihydro-1H-benzo[b]azepine (3l).[1] (White solid was obtained in 61% isolated yield, 190.6 mg). ^{1}H NMR (600 MHz, DMSO-d_6) δ 7.53-7.52 (m, 1H), 7.47-7.45 (m, 4H), 7.33 (d, 1H, *J* = 8.6 Hz), 7.26-7.23 (m, 4H), 7.20 (t, 2H, *J* = 7.3 Hz), 7.05 (d, 2H, *J* = 7.9 Hz), 6.91 (s, 1H), 6.74 (d, 2H, *J* = 7.5 Hz),6.11 (s, 1H), 4.37 (s, 1H), 3.98 (s, 1H),3.13 (d, 2H, *J* = 6.5 Hz), 2.51 (s, 1H), 2.15 (s, 3H). ^{13}C NMR (151 MHz, DMSO-d_6) δ 143.56, 142.28, 139.91, 139.03, 137.66, 137.09, 133.54, 132.88, 132.47, 131.75, 130.08, 130.01, 129.73, 128.53, 128.10, 127.74, 127.37, 127.30, 121.83, 46.44, 28.50, 21.59, 8.94.

HRMS (ESI) *m/z*: [M + H]$^+$ calcd for $C_{30}H_{27}BrNO_2SSe^+$, 624.0106; found, 624.0112.

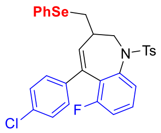

5-(4-chlorophenyl)-6-fluoro-3-((phenylselanyl)methyl)-1-tosyl-2,3-dihydro-1H-benzo[b] azepine (3m). (White solid was obtained in 60% isolated yield, 180.4 mg). ^{1}H NMR (600 MHz, DMSO-d_6) δ 7.49-7.44 (m, 5H), 7.29-7.22 (m, 7H), 7.01 (d, 2H, *J* = 8.0 Hz), 6.75 (d, 2H, *J* = 8.6 Hz), 6.29 (d, 1H, *J* = 6.5 Hz), 4.38 (t, 1H, *J* = 12.3 Hz), 3.95-3.92 (m, 1H), 3.17 (d, 2H, *J* = 5.7 Hz), 2.55-2.51 (m, 1H), 2.16 (s, 3H). ^{13}C NMR (151 MHz, DMSO) δ 159.09 (d, *J* = 416.8 Hz), 143.44, 139.03 (d, *J* = 6.0 Hz), 137.85, 137.22, 134.86, 134.55, 132.71, 132.67, 132.31, 130.64 (d, *J* = 10.6 Hz), 129.87, 129.75, 128.36, 127.86, 127.84, 127.53, 127.26, 126.46 (d, *J* = 13.6 Hz), 116.79 (d, *J* = 21.1 Hz), 62.54, 38.76, 28.13, 21.27. ^{19}F NMR (565 MHz, DMSO) δ -106.36.

HRMS (ESI) *m/z*: [M + H]$^+$ calcd for $C_{30}H_{26}ClFNO_2SSe^+$, 598.0517; found, 598.0508.

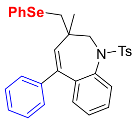

3-methyl-5-phenyl-3-((phenylselanyl)methyl)-1-tosyl-2,3-dihydro-1H-benzo[b]azepine (3n).[4]

(White solid was obtained in 70% isolated yield, 195.5 mg). ^{1}H NMR (600 MHz, DMSO-d_6) δ 7.60 – 7.58 (m, 2H), 7.40 (s, 2H), 7.33 – 7.29 (m, 2H), 7.26 – 7.20 (m, 9H), 6.84 – 6.83 (m, 2H), 6.72 (d, 1H, J = 7.8Hz), 5.94 (s, 1H), 4.14 (s, 2H), 3.06 (s, 2H), 2.26 (s, 3H), 1.18 (s, 3H). ^{13}C NMR (151 MHz, DMSO-d_6) δ 143.63, 142.78, 139.42, 139.30, 138.78, 138.31, 137.81, 132.28, 131.24, 131.01, 130.11, 129.70, 128.89, 128.69, 128.42, 127.79, 127.36, 127.18, 64.67, 38.78, 27.93, 21.38.

HRMS (ESI) m/z: [M + H]$^+$ calcd for $C_{31}H_{30}NO_2SSe^+$, 560.1157; found, 560.1161.

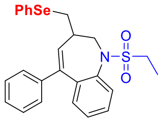

1-(ethylsulfonyl)-5-phenyl-3-((phenylselanyl)methyl)-2,3-dihydro-1H-benzo[b]azepine (3o). (Colorless oil was obtained in 76% isolated yield, 184.5 mg). ^{1}H NMR (600 MHz, DMSO-d_6) δ 7.48-7.45 (m, 3H), 7.38-7.30 (m, 5H), 7.27-7.22 (m, 3H), 7.17 (d, 2H, J = 8.1 Hz), 6.93 (d, 1H, J = 7.5 Hz), 6.26 (d, 1H, J = 6.0 Hz), 4.13 (s, 1H), 3.96 (s, 1H), 3.18-3.06 (m, 3H), 3.03-2.97 (m, 1H), 2.51 (s, 1H), 1.14 (t, 3H, J = 7.3 Hz). ^{13}C NMR (151 MHz, DMSO-d_6) δ 141.32, 140.29, 138.45, 132.35, 131.19, 130.31, 130.22, 129.71, 129.60, 128.99, 128.91, 128.73, 128.19, 128.05, 127.33, 126.89, 63.64, 46.74, 39.08, 27.11, 7.91.

HRMS (ESI) m/z: [M + H]$^+$ calcd for $C_{25}H_{26}NO_2SSe^+$, 484.0844; found, 484.0841.

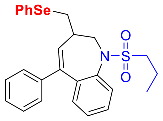

5-phenyl-3-((phenylselanyl)methyl)-1-(propylsulfonyl)-2,3-dihydro-1H-benzo[b]azepine (3p). (Colorless oil was obtained in 75% isolated yield, 186.7 mg). ^{1}H NMR (600 MHz, DMSO-d_6) δ 7.49 – 7.48 (m, 2H), 7.47-7.45 (m, 1H), 7.38 – 7.31 (m, 5H), 7.28 – 7.24 (m, 3H), 7.19 (d, 2H, J = 7.4 Hz), 6.96 (d, 1H, J = 7.5 Hz), 6.29 (d, 1H, J = 6.0 Hz), 4.13 (s, 1H), 3.95 (s, 1H), 3.16 (s, 2H), 3.03 (s, 1H), 2.94 (s, 1H), 2.51 (s, 1H), 1.65 – 1.60 (m, 2H), 0.74 (t, 3H, J = 7.4 Hz).^{13}C NMR (151 MHz, DMSO-d_6) δ 141.14, 139.91, 138.41, 132.38, 131.55, 130.31, 130.22, 129.75, 129.00, 128.90, 128.77, 128.22, 127.94, 127.34, 61.60, 54.28, 38.15, 26.09, 16.81, 12.97.

HRMS (ESI) m/z: [M + H]$^+$ calcd for $C_{26}H_{28}NO_2SSe^+$, 498.1000; found, 498.0999.

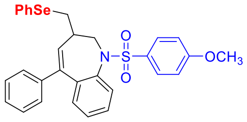

1-((4-methoxyphenyl)sulfonyl)-5-phenyl-3-((phenylselanyl)methyl)-2,3-dihydro-1H-

benzo[b]azepine (3q). (Colorless oil was obtained in 54% isolated yield, 152.7 mg). ^{1}H NMR (600 MHz, DMSO-d_6) δ 7.50 – 7.47 (m, 4H), 7.41 (d, 1H, J = 7.9 Hz), 7.35 (t, 1H, J = 7.5), 7.30 – 7.20 (m, 7H), 6.86 (s, 1H), 6.79 (d, 2H, J = 7.4 Hz), 6.75 (d, 2H, J = 8.2 Hz), 6.09 (s, 1H), 4.41 (s, 1H), 4.00 (s, 1H), 3.66 (s, 3H), 3.12 (s, 2H), 2.52 (s, 1H).^{13}C NMR (151 MHz, DMSO) δ 162.68, 141.00, 139.82, 137.89, 132.52, 132.35, 131.51, 130.24, 129.75, 129.45, 128.83, 128.66, 128.37, 127.85, 127.33, 114.53, 63.17, 55.85, 38.72, 28.70.

HRMS (ESI) m/z: $[M + H]^+$ calcd for $C_{30}H_{28}NO_3SSe^+$,562.0950; found, 562.0954.

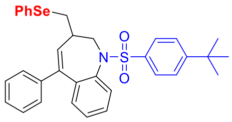

1-((4-(tert-butyl)phenyl)sulfonyl)-5-phenyl-3-((phenylselanyl)methyl)-2,3-dihydro-1H-benzo[b]azepine (3r). (White solid was obtained in 55% isolated yield, 160.7 mg). ^{1}H NMR (600 MHz, DMSO-d_6) δ 7.52 (d, 2H, J = 6.3 Hz), 7.47 (d, 2H, J = 7.6 Hz), 7.41 (d, 1H, J = 8.1 Hz), 7.35 (t, 1H, J = 7.5 Hz), 7.29-7.19 (m, 9H), 6.89 (s, 3H), 5.98 (s, 1H), 4.33 (s, 1H), 4.00 (s, 1H), 3.14 (d, 2H, J = 5.9 Hz), 2.51 (s, 1H), 1.11 (s, 9H).^{13}C NMR (151 MHz, DMSO-d_6) δ 155.85, 141.43, 140.63, 139.79, 139.61, 138.14, 132.34, 131.52, 131.16, 130.26, 129.73, 129.27, 128.85, 128.43, 128.02, 127.86, 127.32, 127.07, 126.30, 63.12, 38.57, 35.04, 31.08, 28.77.

HRMS (ESI) m/z: $[M + H]^+$ calcd for $C_{33}H_{34}NO_2SSe^+$,588.1470; found, 588.1477.

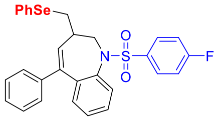

1-((4-fluorophenyl)sulfonyl)-5-phenyl-3-((phenylselanyl)methyl)-2,3-dihydro-1H-benzo[b] azepine (3s). (Colorless oil was obtained in 56% isolated yield, 154.4 mg). ^{1}H NMR (600 MHz, DMSO-d_6) δ 7.63 (s, 2H), 7.46 (d, 2H, J = 5.8 Hz), 7.40 (d, 1H, J = 7.9 Hz), 7.36 (t, 1H, J = 7.5 Hz), 7.32-7.31 (m, 1H), 7.27-7.23 (m, 4H), 7.21-7.19 (m, 2H), 7.02 (s, 2H), 6.86 (s, 1H), 6.80 (d, 2H, J =7.6 Hz), 6.09 (s, 1H), 4.36 (s, 1H), 4.02 (s, 1H), 3.15 (d, 2H, J = 6.1 Hz), 2.50 (s, 1H). ^{13}C NMR (151 MHz, DMSO) δ 164.59 (d, J = 150.7 Hz), 140.88, 139.87, 139.37, 137.40, 136.72, 132.37, 131.74, 131.46, 130.32 (d, J = 10.6 Hz), 130.14, 129.75, 129.00, 128.42, 127.99, 127.59, 127.36, 116.525 (d, J = 22.6 Hz), 62.90, 38.65, 28.58. ^{19}F NMR (565 MHz, DMSO) δ -106.49.

HRMS (ESI) m/z: $[M + H]^+$ calcd for $C_{29}H_{25}FNO_2SSe^+$, 550.0750; found, 550.0745.

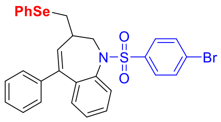

1-((4-bromophenyl)sulfonyl)-5-phenyl-3-((phenylselanyl)methyl)-2,3-dihydro-1H-benzo[b]azepine (3t). (Colorless oil was obtained in 51% isolated yield, 155.7 mg). ^{1}H NMR (600 MHz, DMSO-d_6) δ 7.49-7.47 (m, 4H), 7.42-7.38 (m, 4H), 7.28-7.25 (m, 4H), 7.23-7.20 (m, 2H), 6.87 (s, 1H), 6.76 (d, 2H, J = 7.7 Hz), 6.08 (s, 1H), 4.40 (s, 1H), 4.04 (s, 1H), 3.17 (d, 2H, J = 6.0 Hz), 2.51 (s, 2H). ^{13}C NMR (151 MHz, DMSO-d_6) δ 140.83, 140.00, 139.83, 139.27, 137.25, 132.51, 132.37, 131.84, 131.57, 130.35, 130.16, 129.77, 129.27, 129.04, 128.46, 128.04, 127.52, 127.40, 127.09, 67.42, 38.62, 28.51.

HRMS (ESI) m/z: $[M + H]^+$ calcd for $C_{29}H_{25}BrNO_2SSe^+$, 609.9949; found, 609.9940.

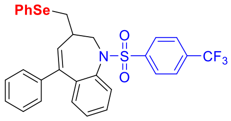

5-phenyl-3-((phenylselanyl)methyl)-1-((4-(trifluoromethyl)phenyl)sulfonyl)-2,3-dihydro-1H-benzo[b]azepine (3u). (White solid was obtained in 62% isolated yield, 185.5 mg). ^{1}H NMR (600 MHz, DMSO-d_6) δ 7.80 (d, 2H, J = 8.0 Hz), 7.56 (d, 2H, J = 8.0 Hz), 7.49-7.45 (m, 3H), 7.40 (t, 1H, J = 6.8 Hz), 7.37-7.33 (m, 1H), 7.28-7.24 (m, 3H), 7.22-7.20 (m, 1H), 7.17-7.14 (m, 2H), 6.87 (s, 1H), 6.75 (d, 2H, J = 8.6 Hz), 6.06 (s, 1H), 4.46 (s, 1H), 4.09 (s, 1H), 3.18 (d, 2H, J =5.7 Hz), 2.51 (s, 1H). ^{13}C NMR (151 MHz, DMSO) δ 144.56, 140.69, 139.68, 139.15, 137.07, 132.58 (q, J =31.7 Hz), 132.39, 132.11, 131.37, 130.41, 130.15, 129.75, 129.21, 129.12, 128.34, 128.27, 127.99, 127.43, 127.36, 126.54, 123.65 (q, J =273.3 Hz), 64.46, 37.70, 29.02. ^{19}F NMR (565 MHz, DMSO) δ -61.73.

HRMS (ESI) m/z: $[M + H]^+$ calcd for $C_{30}H_{25}F_3NO_2SSe^+$, 600.0718; found, 600.0726.

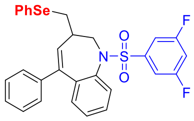

1-((3,5-difluorophenyl)sulfonyl)-5-phenyl-3-((phenylselanyl)methyl)-2,3-dihydro-1H-benzo[b]azepine (3v). (Colorless oil was obtained in 46% isolated yield, 131.0 mg). ^{1}H NMR (600 MHz, DMSO-d_6) δ 7.48-7.46 (m, 2H), 7.44-7.36 (m, 3H), 7.30 (d, 2H, J = 3.4 Hz), 7.28-7.24 (m, 4H), 7.23-7.20 (m, 2H), 7.13 (s, 1H), 6.91 (d, 1H, J = 7.4 Hz), 6.85 (d, 2H, J = 7.5 Hz), 6.15 (s, 1H), 4.47 (s, 1H), 4.08 (s, 1H), 3.24-3.16 (m, 2H), 2.51 (s, 1H). ^{13}C NMR (151 MHz, DMSO) δ 162.26

(d, J = 250.6 Hz), 143.89, 140.48, 139.61, 139.05, 136.92, 132.38, 132.05, 131.39, 130.39, 130.13, 129.75, 129.30, 129.20, 128.46, 128.10, 127.37, 127.23, 111.32 (d, J = 25.7 Hz), 108.57, 61.30, 37.38, 28.38. ^{19}F NMR (565 MHz, DMSO) δ -106.51.

HRMS (ESI) *m/z*: $[M + H]^+$ calcd for $C_{29}H_{24}F_2NO_2SSe^+$, 568.0656; found, 568.0663.

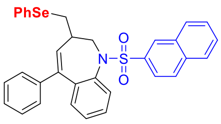

1-(naphthalen-2-ylsulfonyl)-5-phenyl-3-((phenylselanyl)methyl)-2,3-dihydro-1H-benzo[b]azepine (3w). (White solid was obtained in 78% isolated yield, 226.5 mg). ^{1}H NMR (600 MHz, DMSO-d_6) δ 8.19 (s, 1H), 7.95 (d, 1H, J = 8.1 Hz), 7.81 (s, 1H), 7.74 (s, 1H), 7.65-7.57 (m, 3H), 7.45-7.26 (m, 8H), 7.03 (s, 1H), 6.90 (s, 2H), 6.80 (s, 1H), 6.61 (s, 2H), 6.05 (s, 1H), 4.58 (s, 1H), 4.08 (s, 1H), 3.18 (s, 1H), 2.51 (s, 2H). ^{13}C NMR (151 MHz, DMSO-d_6) δ 140.99, 140.09, 139.43, 137.84, 137.61, 134.60, 132.33, 132.00, 131.61, 130.18, 129.76, 129.59, 129.09, 128.95, 128.26, 128.11, 128.07, 127.81, 127.71, 127.34, 122.91, 63.38, 39.00, 28.58.

HRMS (ESI) *m/z*: $[M + H]^+$ calcd for $C_{33}H_{28}NO_2SSe^+$, 582.1000; found, 582.1001.

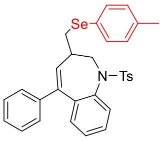

5-phenyl-3-((p-tolylselanyl)methyl)-1-tosyl-2,3-dihydro-1H-benzo[b]azepine (3x).[1] (Colorless oil was obtained in 56% isolated yield, 156.4 mg). ^{1}H NMR (600 MHz, DMSO-d_6) δ 7.45 (d, 2H, J = 8.0 Hz), 7.38-7.33 (m, 4H), 7.31-7.29 (m, 1H), 7.26-7.24 (m, 1H), 7.22-7.20 (m, 2H), 7.09-7.05 (m, 4H), 6.84 (d, 1H, J = 7.5 Hz), 6.76 (d, 2H, J = 7.5 Hz), 6.04 (s, 1H), 4.34 (s, 1H), 3.94 (s, 1H),3.10 (d, 2H, J =6.1 Hz), 2.51 (s, 1H), 2.25 (s, 3H), 2.18 (s, 3H). ^{13}C NMR (151 MHz, DMSO) δ 143.32, 140.05, 138.05, 137.66, 137.03, 134.63, 134.22, 132.95, 131.37, 130.41, 129.91, 129.37, 128.82, 128.67, 128.36, 127.82, 127.28, 126.19, 63.22, 38.77, 28.96, 21.33, 21.06.

HRMS (ESI) *m/z*: $[M + H]^+$ calcd for $C_{31}H_{30}NO_2SSe^+$, 560.1157; found, 560.1164.

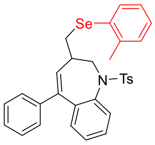

5-phenyl-3-((o-tolylselanyl)methyl)-1-tosyl-2,3-dihydro-1H-benzo[b]azepine (3y).[1] (Colorless oil was obtained in 83% isolated yield, 233.0 mg). ^{1}H NMR (600 MHz, DMSO-d_6) δ 7.48 (d, 2H, J

= 7.9 Hz), 7.45 (d, 1H, J = 7.7 Hz), 7.41 (d, 1H, J = 7.8 Hz), 7.36 (t, 1H, J = 5.9 Hz), 7.33-7.31 (m, 1H), 7.27-7.22 (m, 4H), 7.16 (t, 1H, J = 6.4 Hz), 7.13-7.07 (m, 3H), 6.86-6.82 (m, 3H), 6.12 (s, 1H), 4.40 (s, 1H), 4.04 (s, 1H), 3.16-3.11 (m, 2H), 2.53 (s, 1H), 2.30 (s, 3H), 2.19 (s, 3H). ^{13}C NMR (151 MHz, DMSO) δ 142.86, 141.23, 139.97, 138.74, 137.66, 131.94, 131.57, 131.14, 130.63, 130.43, 130.28, 129.90, 128.84, 128.69, 128.38, 127.84, 127.30, 127.16, 126.72, 62.40, 38.56, 28.14, 22.32, 20.02.

HRMS (ESI) *m/z*: $[M + H]^+$ calcd for $C_{31}H_{30}NO_2SSe^+$, 560.1157; found, 560.1164.

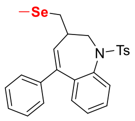

3-((methylselanyl)methyl)-5-phenyl-1-tosyl-2,3-dihydro-1H-benzo[b]azepine (3z).[1] (White solid was obtained in 48% isolated yield, 115.8 mg). ^{1}H NMR (400 MHz, DMSO-d_6) δ 7.50 (d 2H, J = 8.0 Hz,), 7.45 – 7.42 (m, 2H), 7.33-7.29 (m, 1H), 7.26-7.22 (m, 3H), 7.08 (d, 2H, J = 8.2 Hz), 6.88 (d, 3H, J = 8.0 Hz), 6.10 (s, 1H), 4.33 (s, 1H), 4.04 (s, 1H), 2.73 (d, 2H, J = 5.6 Hz), 2.43 (s, 1H), 2.18 (s, 3H), 1.92 (s, 3H). ^{13}C NMR (101 MHz, DMSO) δ 142.94, 140.04, 138.10, 132.17, 131.28, 130.32, 129.93, 128.83, 128.69, 128.42, 127.86, 127.33, 127.09, 62.98, 38.47, 26.75, 21.35, 5.68.

HRMS (ESI) *m/z*: $[M + H]^+$ calcd for $C_{25}H_{26}NO_2SSe^+$, 484.0844; found, 484.0843.

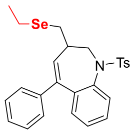

3-((ethylselanyl)methyl)-5-phenyl-1-tosyl-2,3-dihydro-1H-benzo[b]azepine (3za). (Colorless oil was obtained in 49% isolated yield, 121.8 mg). ^{1}H NMR (600 MHz, DMSO-d_6) δ 7.48 (d, 2H, J = 8.0 Hz), 7.41 (d, 1H, J = 7.9 Hz), 7.37 (t, 1H, J = 6.0 Hz), 7.34-7.31 (m, 1H), 7.28-7.23 (m , 3H), 7.09 (d, 2H, J = 7.9 Hz), 6.86 (d, J = 6.8 Hz, 3H), 6.05 (s, 1H), 4.32 (s, 1H), 4.02 (s, 1H), 2.76 (d, 2H, J = 6.5 Hz), 2.54-2.51 (m, 3H), 2.19 (s, 3H), 1.29 (t, 3H, J = 7.5 Hz). ^{13}C NMR (151 MHz, DMSO) δ 143.37, 140.81, 139.90, 138.05, 132.03, 131.23, 129.94, 128.86, 128.74, 128.44, 127.86, 127.31, 126.68, 62.79, 24.81, 21.33, 17.95, 16.30.

HRMS (ESI) *m/z*: $[M + H]^+$ calcd for $C_{26}H_{28}NO_2SSe^+$, 498.1000; found, 498.0992.

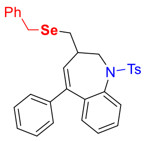

3-((benzylselanyl)methyl)-5-phenyl-1-tosyl-2,3-dihydro-1H-benzo[b]azepine (3zb). (Colorless oil was obtained in 30% isolated yield, 83.8 mg). ^{1}H NMR (600 MHz, DMSO-d_6) δ 7.49 – 7.44 (m, 3H), 7.40-7.34 (m, 2H), 7.27-7.21 (m, 8H), 7.08 (s, 2H), 6.89 (s, 1H), 6.82 (d, 2H, J = 7.5 Hz), 5.97 (s, 1H), 4.28 (s, 1H), 3.90 (s, 1H), 3.79 (s, 1H), 2.73 (s, 2H), 2.51 (s, 2H), 2.19 (s, 3H). ^{13}C NMR (151 MHz, DMSO) δ 143.35, 140.05, 138.05, 129.93, 129.40, 129.24, 129.14, 128.97, 128.90, 128.79, 128.47, 128.40, 128.31, 127.84, 127.42, 127.29, 127.02, 32.30, 32.09, 27.73, 25.25, 21.34. HRMS (ESI) *m/z*: $[M + H]^+$ calcd for $C_{31}H_{30}NO_2SSe^+$, 560.1157; found, 560.1151.

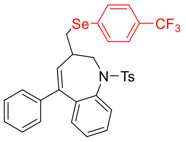

3-((benzylselanyl)methyl)-5-phenyl-1-tosyl-2,3-dihydro-1H-benzo[b]azepine (3zc).[1] (Colorless oil was obtained in 30% isolated yield, 91.9 mg). ^{1}H NMR (600 MHz, DMSO-d_6) δ 7.69 (d, 2H, J = 8.2 Hz), 7.61 (d, 2H, J = 8.2 Hz), 7.46 (d, 2H, J = 8.2 Hz), 7.40 – 7.34 (m, 2H), 7.31 – 7.29 (m, 1H), 7.27 – 7.24 (m, 1H), 7.21-7.18 (m, 2H), 7.07 (d, 2H, J = 7.9 Hz), 6.84 (s, 1H), 6.74 (s, 2H), 6.05 (s, 1H), 4.41 (s, 1H), 4.04 (s, 1H), 3.31-3.27 (m, 2H), 2.51 (s, 1H), 2.18 (s, 3H). ^{13}C NMR (151 MHz, DMSO) δ 143.36, 140.98, 139.72, 138.02, 137.04, 131.68, 131.15, 130.85, 130.72, 130.26, 129.92, 128.91, 128.72, 128.34, 127.84 (q, J =16.61 Hz), 127.49, 127.28, 127.06, 126.22, 126.19, 125.79, 124.49 (q, J =341.26 Hz), 53.58, 37.45, 28.11, 21.31. ^{19}F NMR (565 MHz, DMSO) δ -60.74. HRMS (ESI) *m/z*: $[M + H]^+$ calcd for $C_{31}H_{27}F_3NO_2SSe^+$, 614.0874; found, 614.0879.

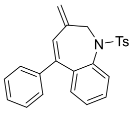

3-methylene-5-phenyl-1-tosyl-2,3-dihydro-1H-benzo[b]azepine (4a).[1] (White solid was obtained in 60% isolated yield, 69.7 mg). ^{1}H NMR (600 MHz, DMSO-d_6) δ 7.56 (d, 1H, J = 8.0 Hz), 7.38-7.35 (m , 3H), 7.29 – 7.27 (m, 3H), 7.20 (t, 1H, J = 7.6 Hz), 7.17 (d, J = 8.1 Hz, 2H), 6.77-6.76 (m, 2H), 6.73 (d, 1H, J = 8.0 Hz), 6.08 (s, 1H), 5.31 (d, 2H, J = 15.9 Hz), 4.85 (d, 1H, J = 16.4 Hz), 3.98 (d, 1H, J = 16.3 Hz), 2.18 (s, 3H). ^{13}C NMR (151 MHz, DMSO) δ 145.26, 143.62, 142.29, 139.68, 138.20, 137.01, 136.18, 133.56, 132.53, 130.47, 129.62, 129.19, 128.92, 128.39, 128.07,

127.61, 127.33, 120.53, 55.01, 21.26.

HRMS (ESI) *m/z*: $[M + H]^+$ calcd for $C_{24}H_{22}NO_2SSe^+$, 388.1366; found, 388.1368.

References

(1) Zhang, Z.; Wang, S.; Tan, P.; Gu, X.; Sun, W.; Liu, C.; Chen, J.; Li, J.; Sun, K. $K_2S_2O_8/I^{2-}$ Promoted Electrophilic Selenylative Cyclization To Access Seleno-Benzoazepines. *Org. Lett.* **2022**, *24*, 2288-2293.

(2) Chen, J.; Chen, Z.; Zhao, H.; Zhang, T.; Wang, W.; Zou, Y.; Zhang, X.; Yan, M. Intramolecular addition of diarylmethanols to imines promoted by KO*t*-Bu/DMF: a new synthetic approach to indole derivatives. *Org. Biomol. Chem.* **2016**, *14*, 4071-4076.

(3) Guo, J.; Xu, C.; Wang, L.; Huang, W.; Wang, M. Catalyst-free and selective trifluoromethylative cyclization of acryloanilides using $PhICF_3Cl$. *Org. Biomol. Chem.* **2019**, *17*, 4593-4599.

(4) Tan, P.; Lu, L.; Wang, S.; Wang, J.; Chen, J.; Zhang, Y.; Xie, L.; Yang, S.; Chen, J.; Zhang, Z. Photo- or Electrochemical Cyclization of Dienes with Diselenides to Access Seleno-Benzoazepines. *The Journal of Organic Chemistry* **2023**, *88*, 7245-7255.

Copies of ^{1}H NMR, ^{13}C NMR and ^{19}F NMR spectra

^{1}H NMR (400 MHz, $CDCl_3$) of compound **3a**

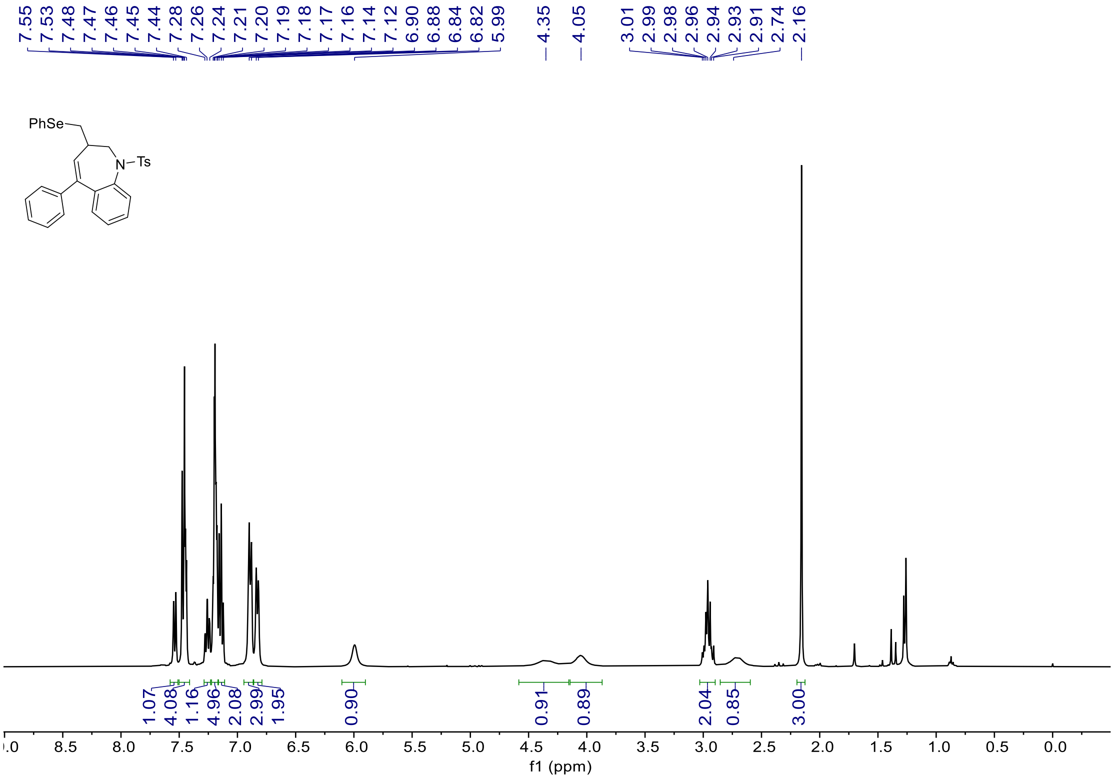

^{13}C NMR (101 MHz, $CDCl_3$) of compound **3a**

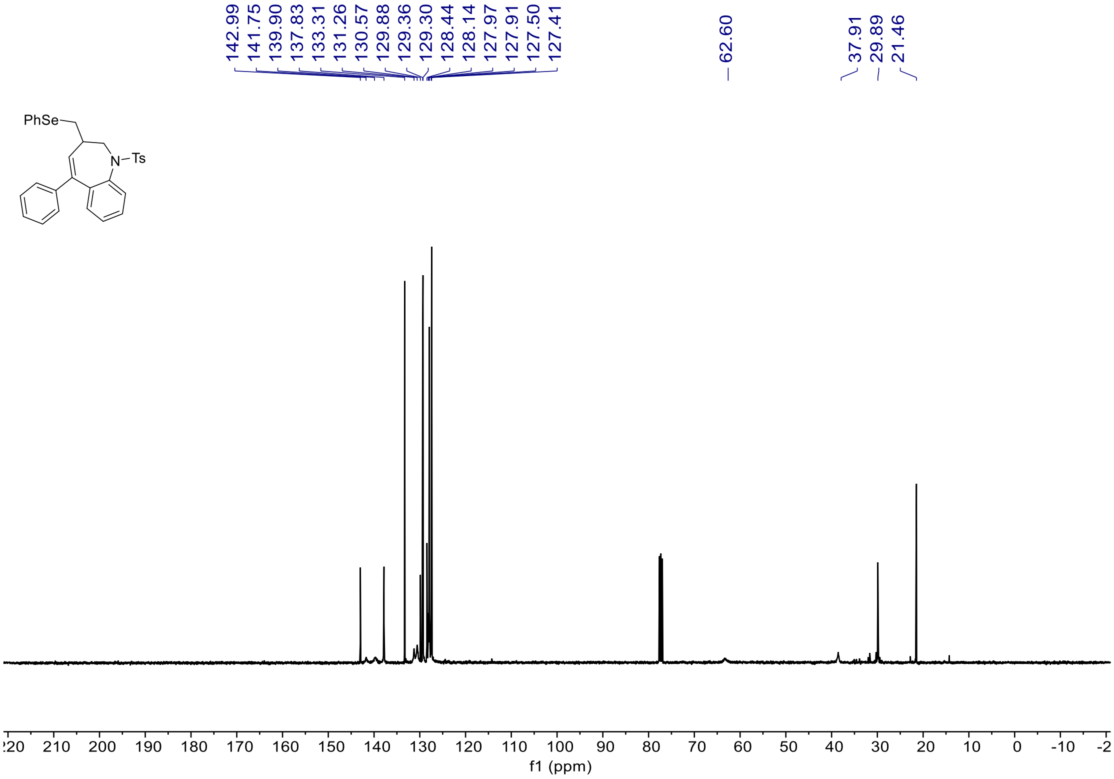

^{1}H NMR (600 MHz, DMSO-d_6) of compound **3b**

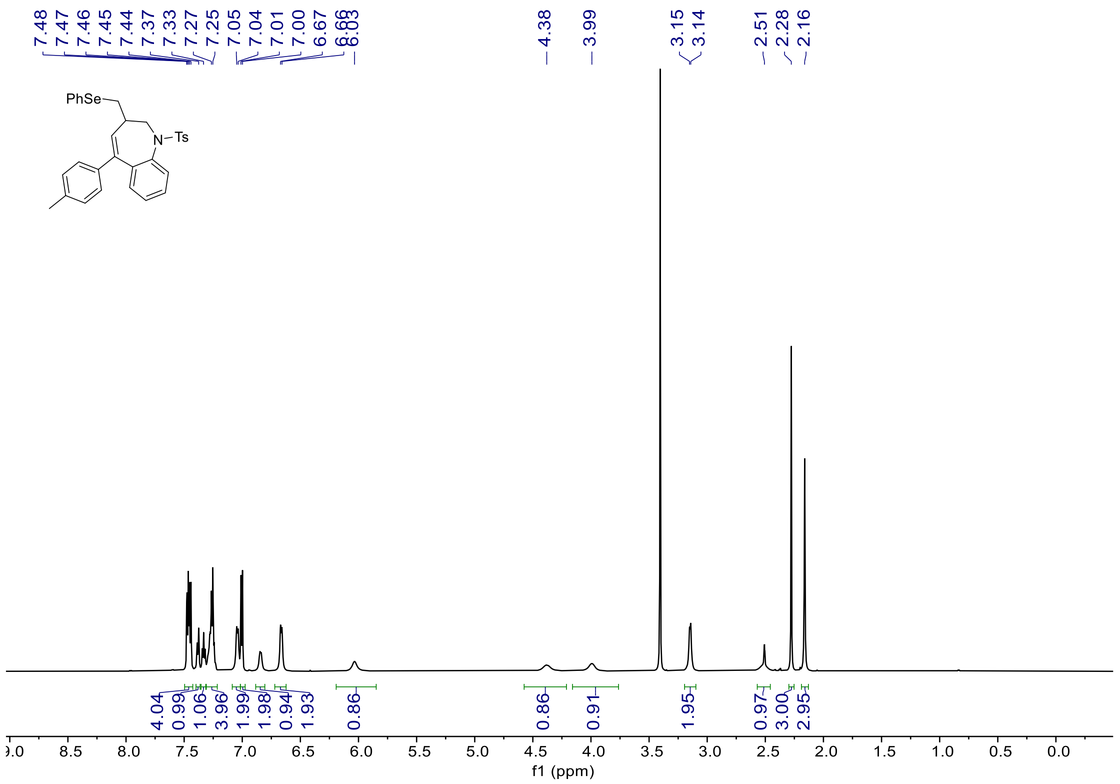

^{13}C NMR (151 MHz, DMSO-d_6) of compound **3b**

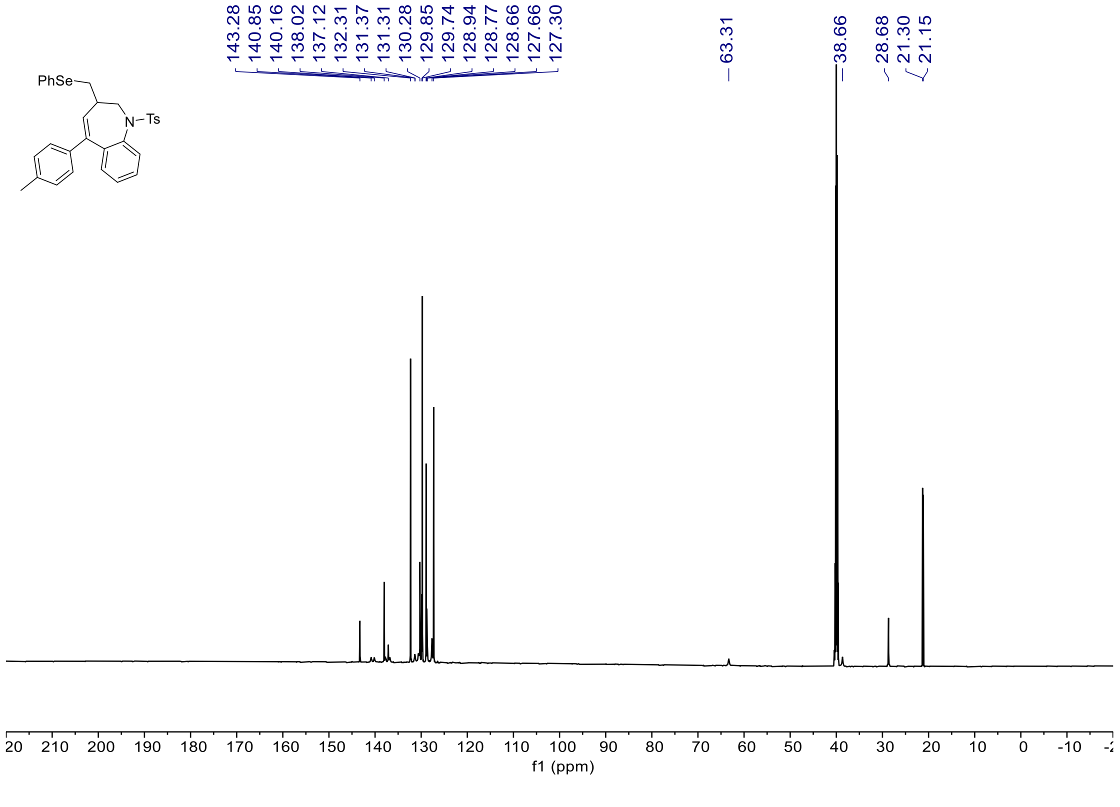

^{1}H NMR (600 MHz, DMSO-d_6) of compound **3c**

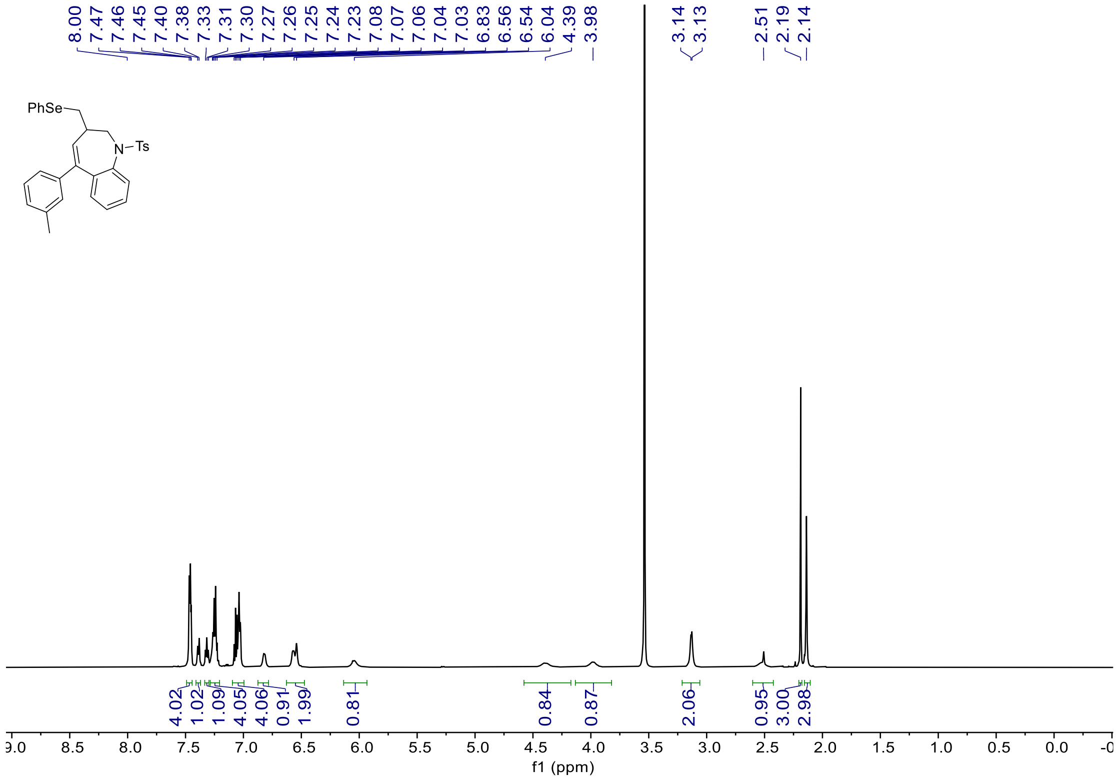

^{13}C NMR (151 MHz, DMSO-d_6) of compound **3c**

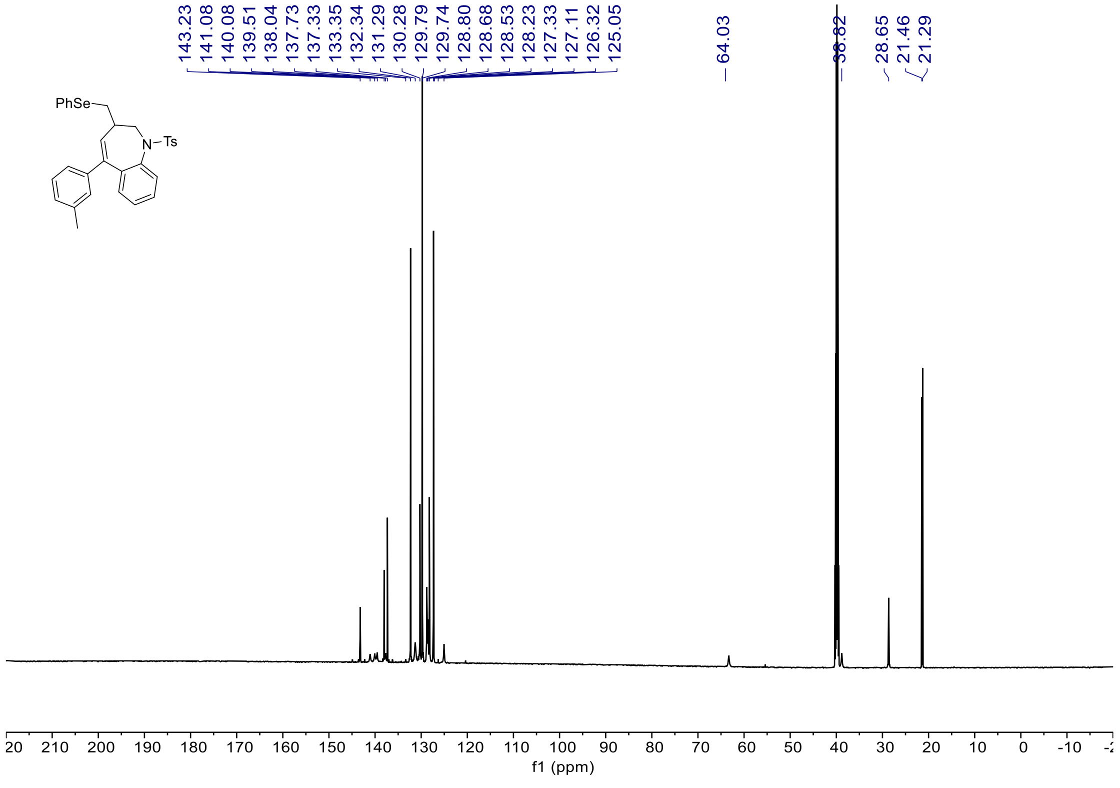

^{1}H NMR (600 MHz, DMSO-d_6) of compound **3d**

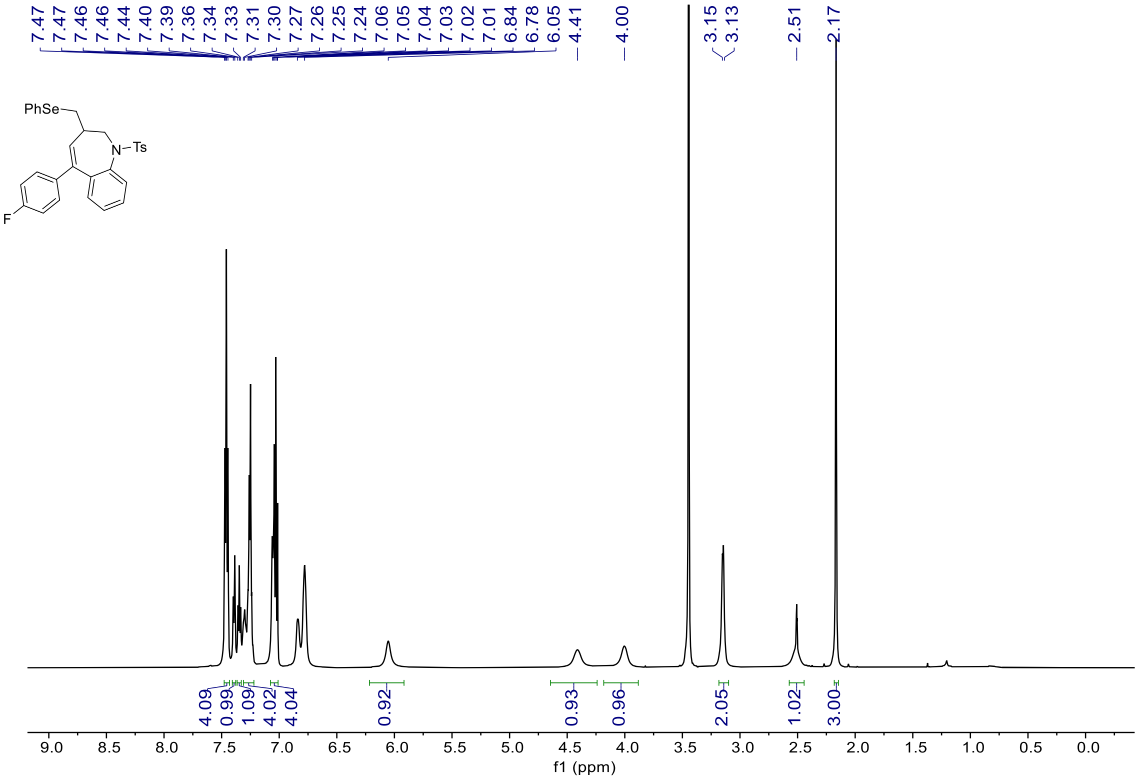

^{13}C NMR (151 MHz, DMSO-d_6) of compound **3d**

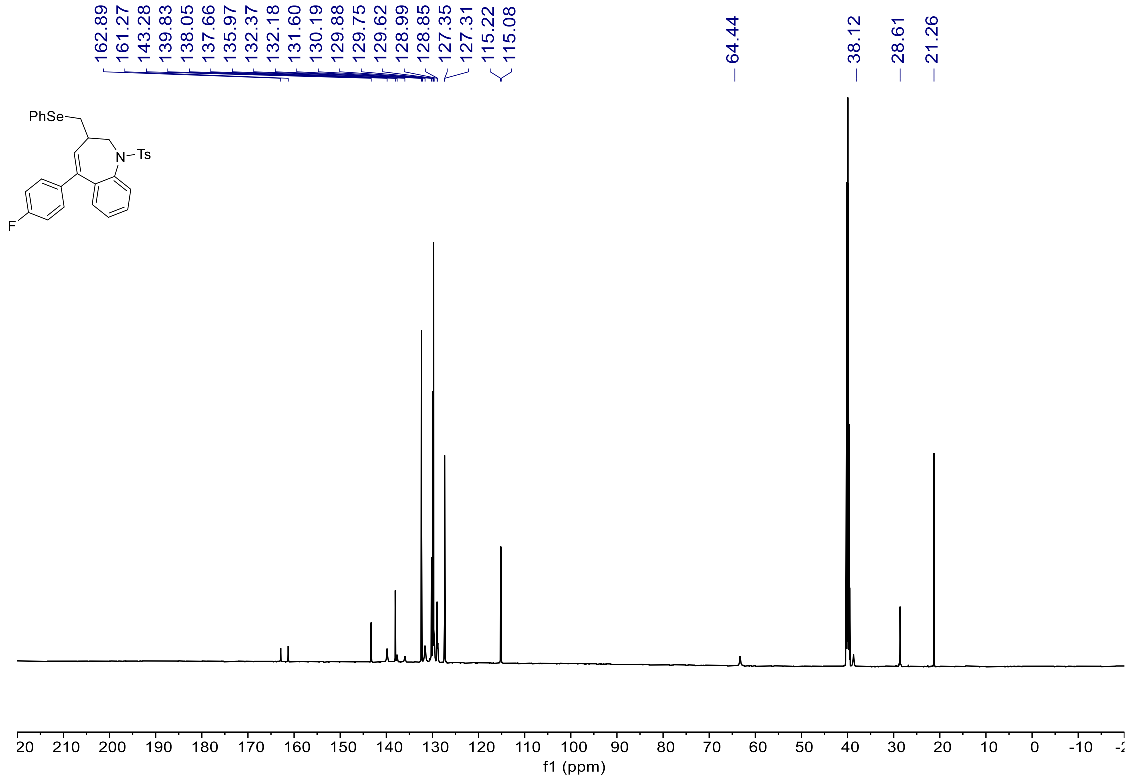

^{19}F NMR (565 MHz, DMSO-d_6) of compound **3d**

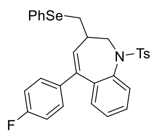

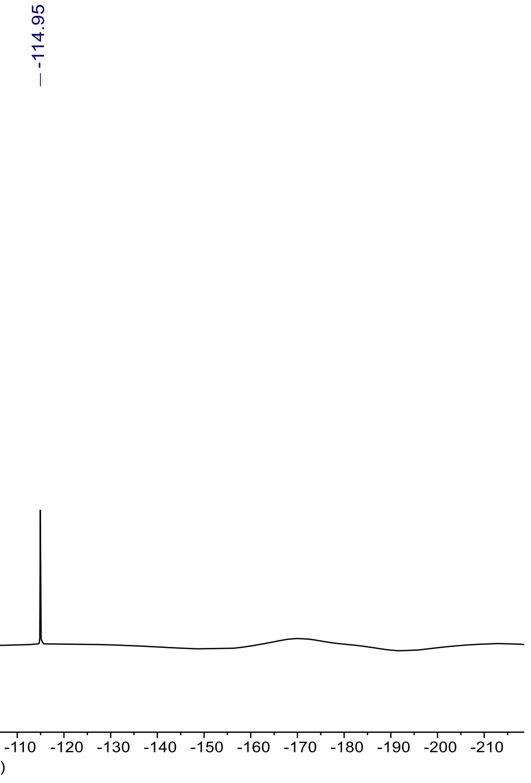

^{1}H NMR (600 MHz, DMSO-d_6) of compound **3e**

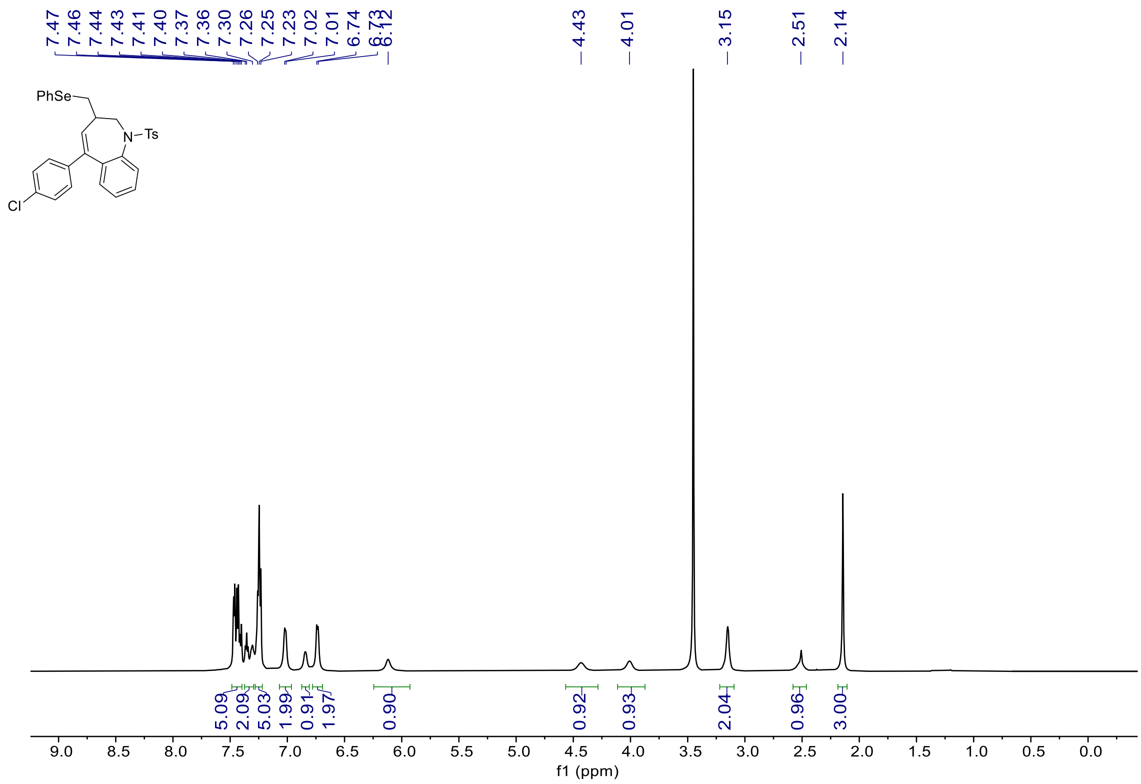

^{13}C NMR (151 MHz, DMSO-d_6) of compound **3e**

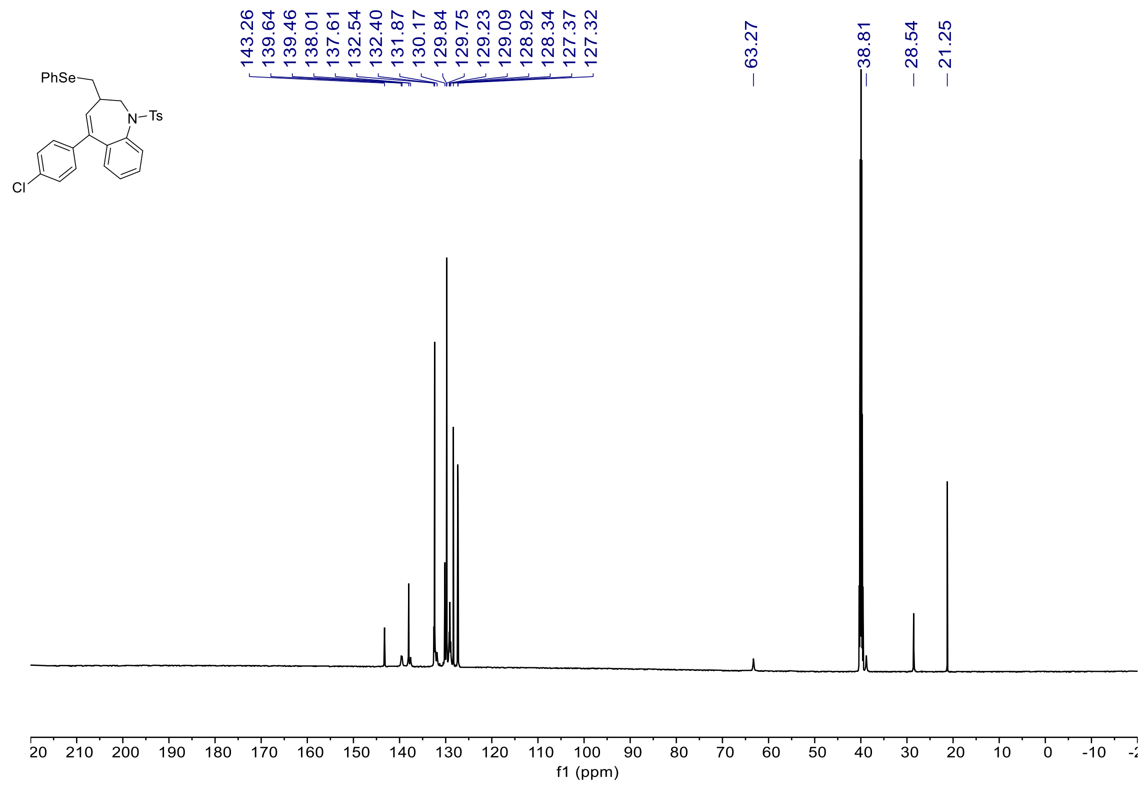

^{1}H NMR (600 MHz, DMSO-d_6) of compound **3f**

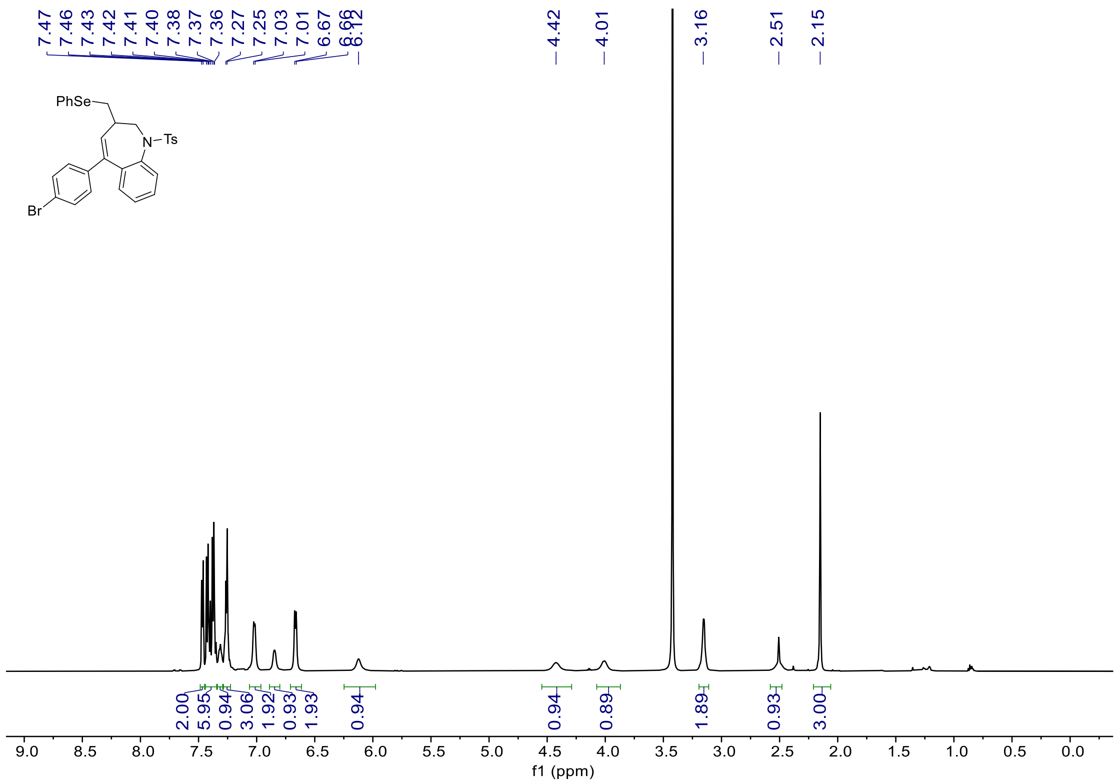

^{13}C NMR (151 MHz, DMSO-d_6) of compound **3f**

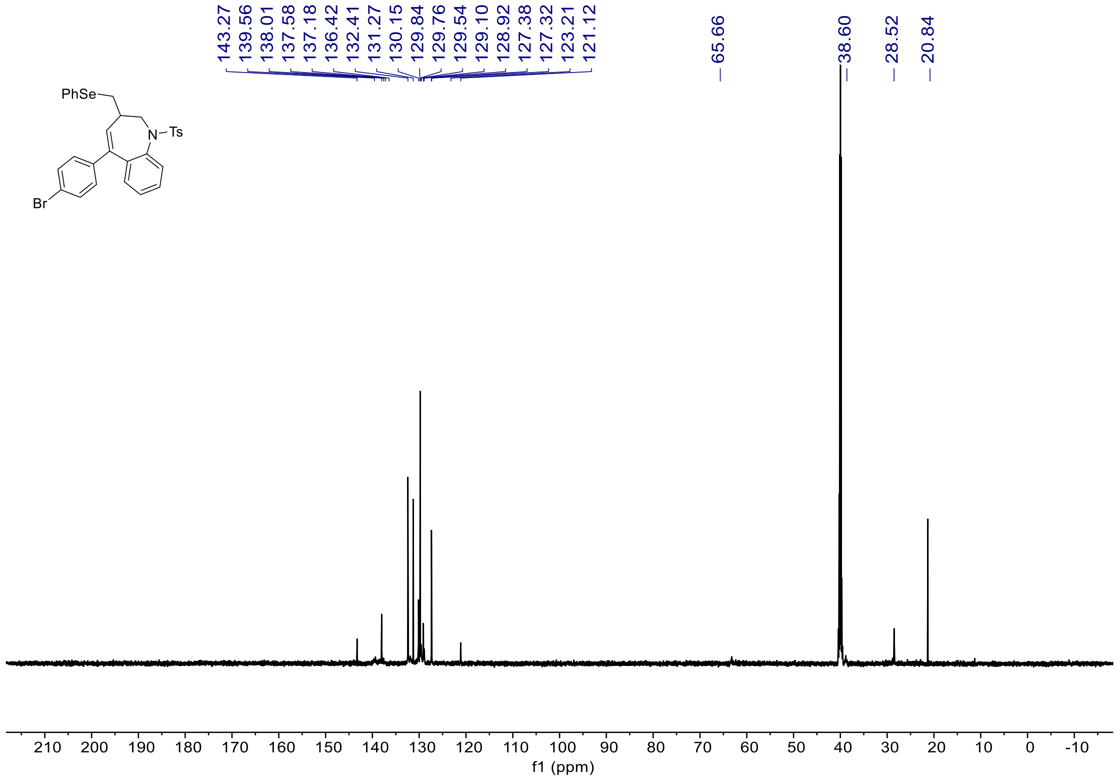

^{1}H NMR (600 MHz, DMSO-d_6) of compound **3g**

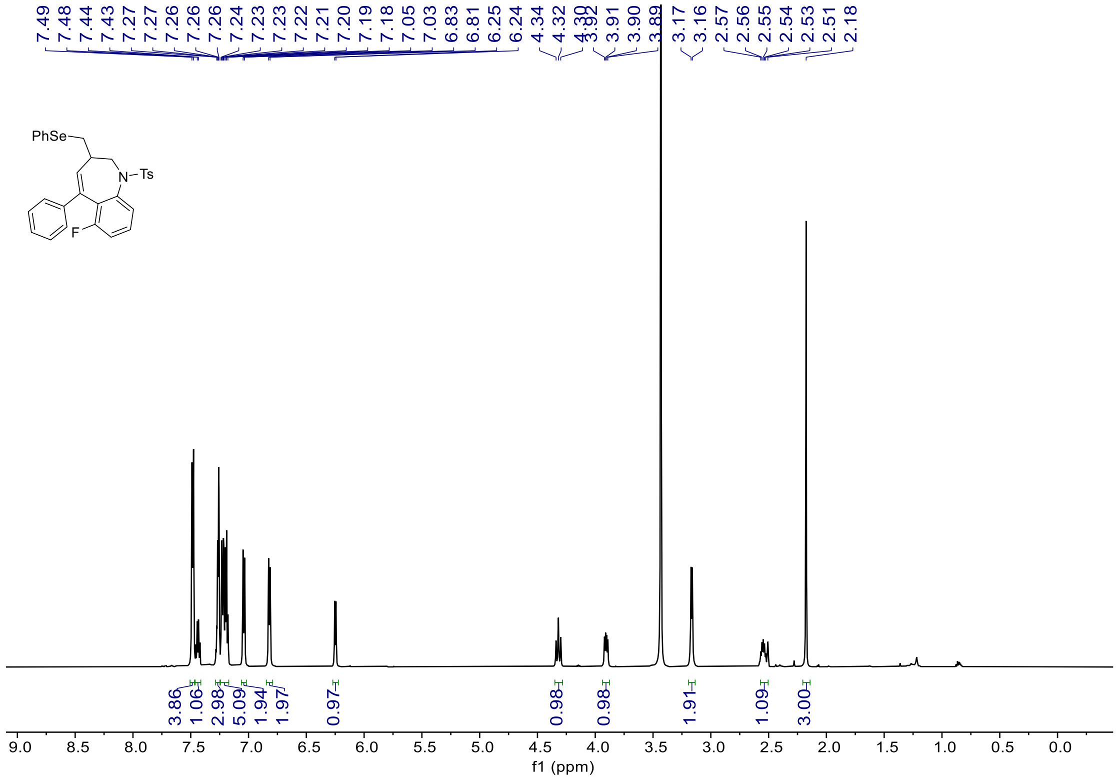

^{13}C NMR (151 MHz, DMSO-d_6) of compound **3g**

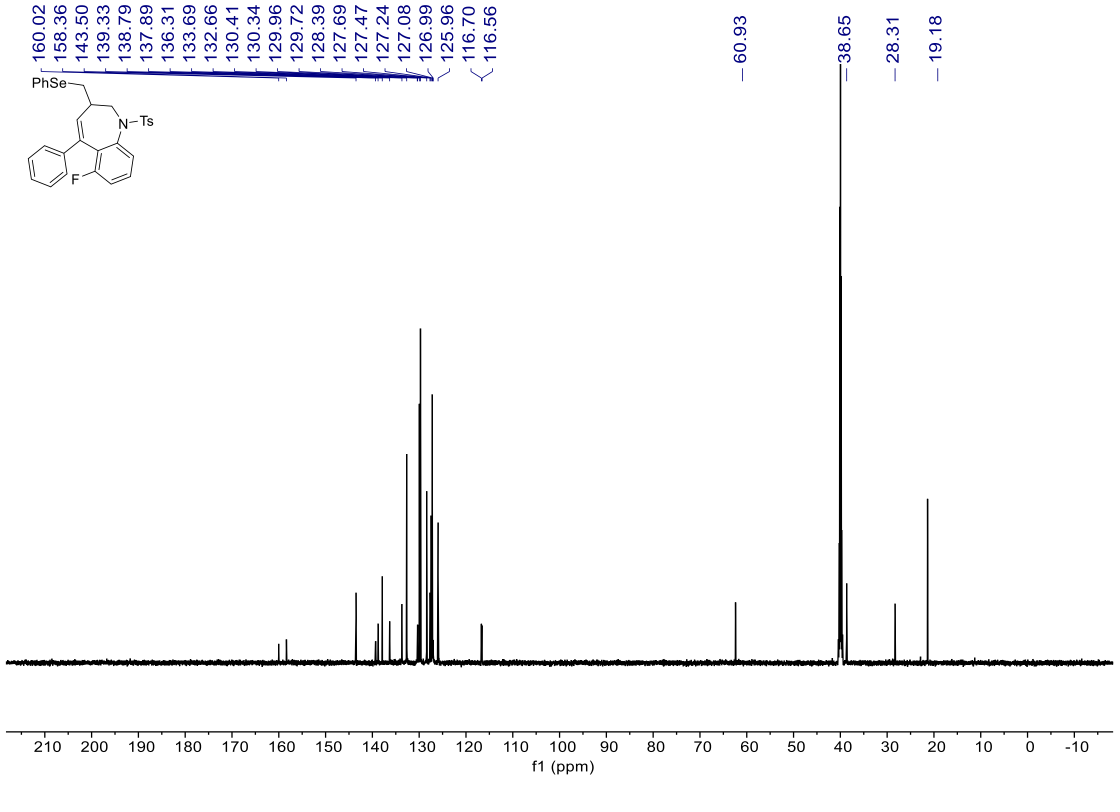

^{19}F NMR (565 MHz, DMSO-d_6) of compound **3g**

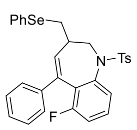

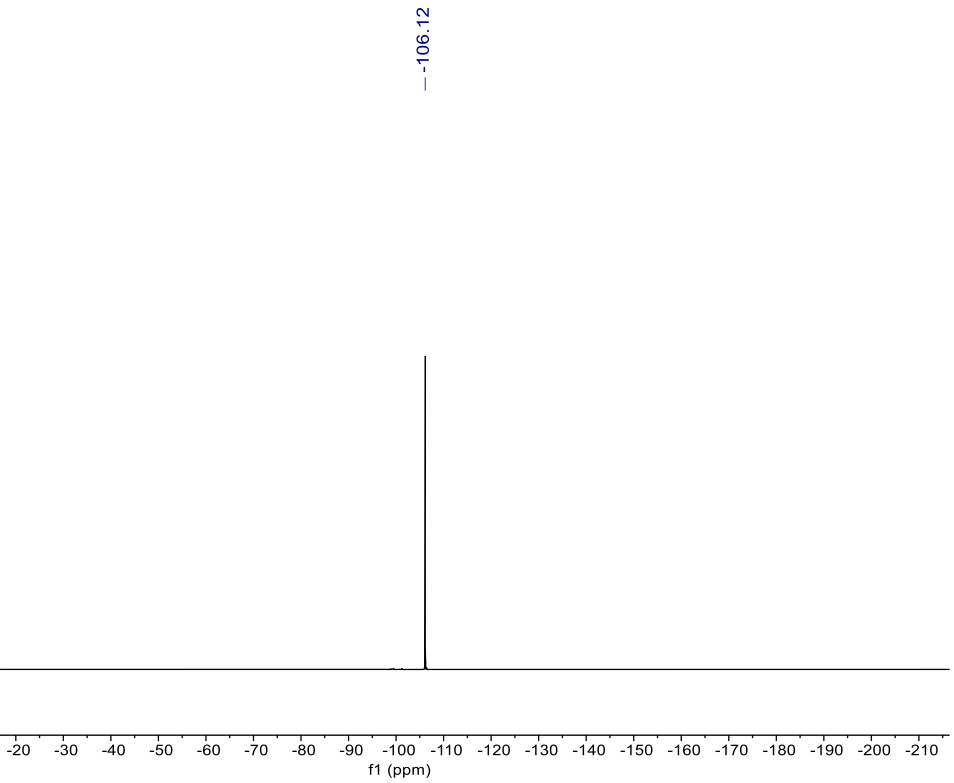

^{1}H NMR (600 MHz, DMSO-d_6) of compound **3h**

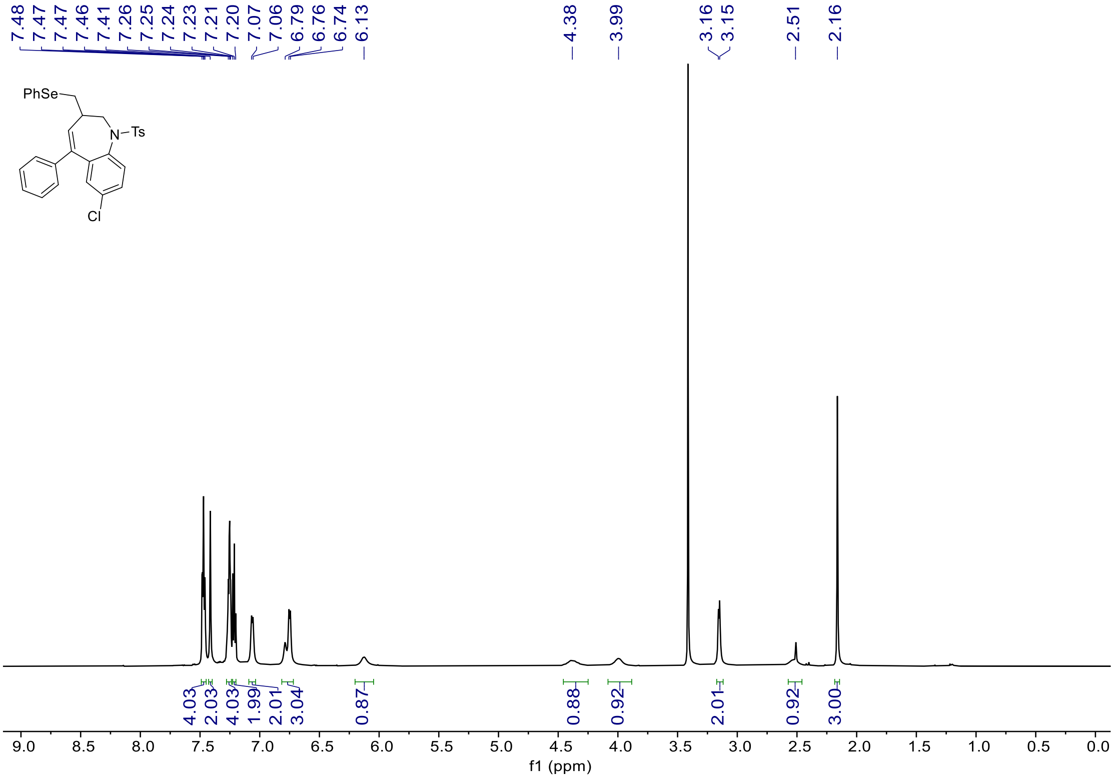

^{13}C NMR (151 MHz, DMSO-d_6) of compound **3h**

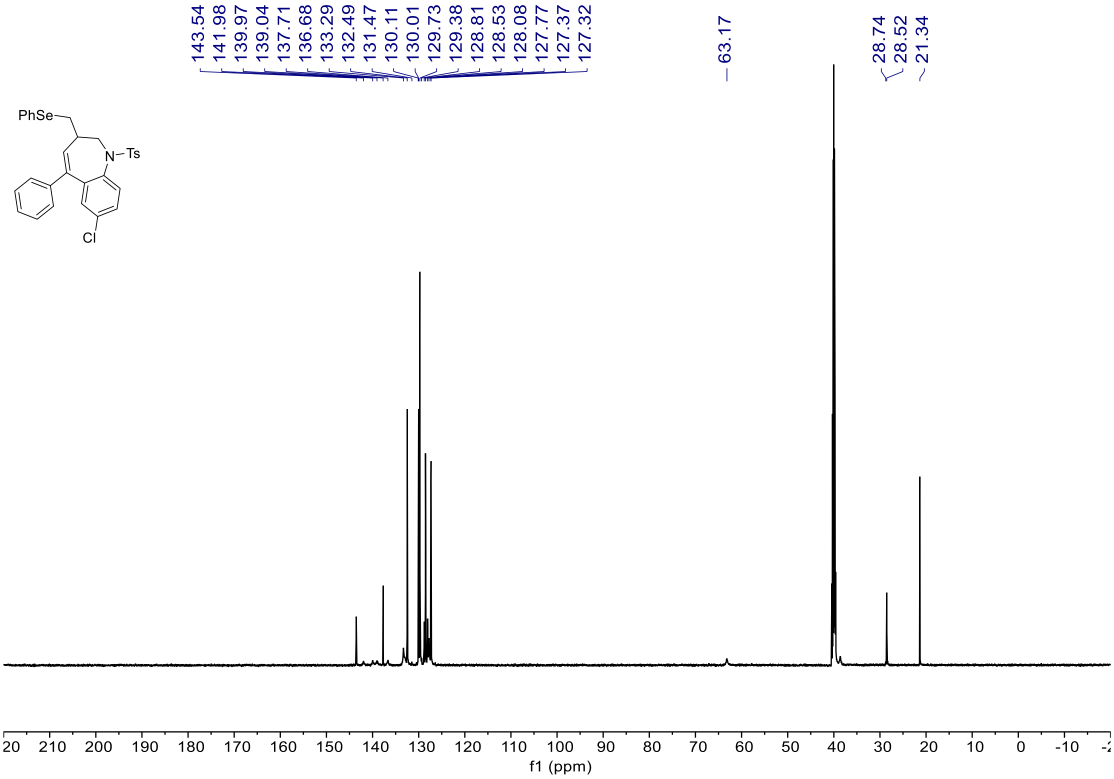

^{1}H NMR (600 MHz, DMSO-d_6) of compound **3i**

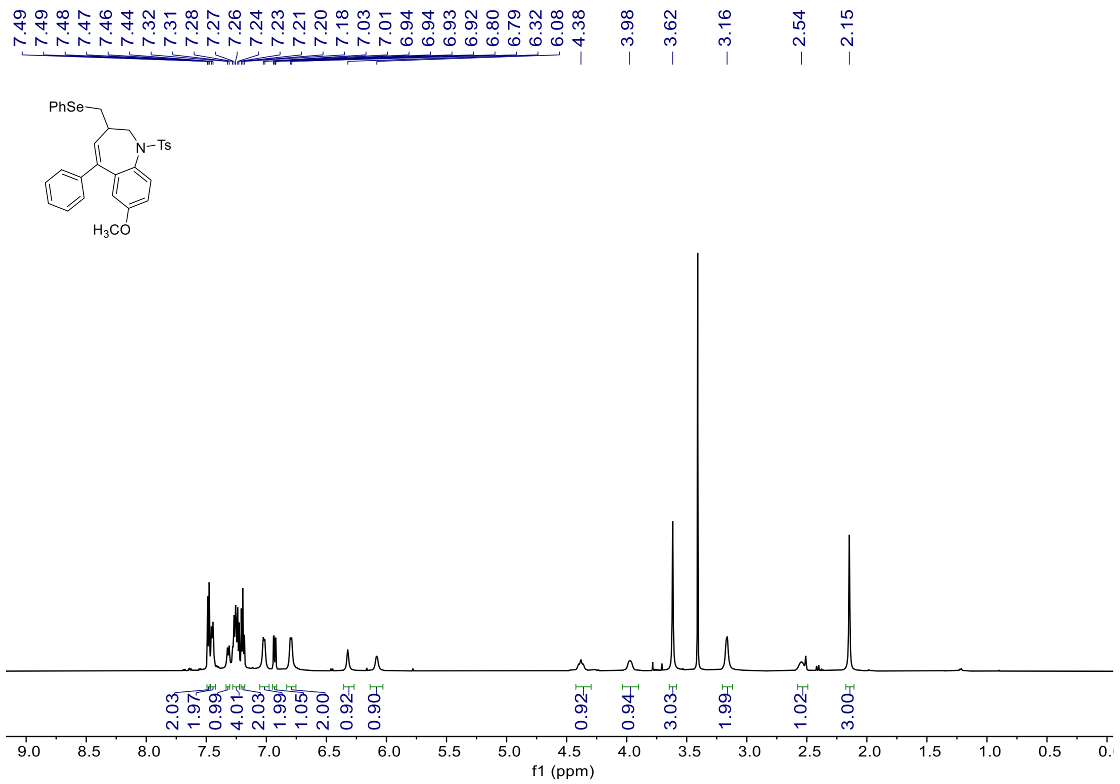

^{13}C NMR (151 MHz, DMSO-d_6) of compound **3i**

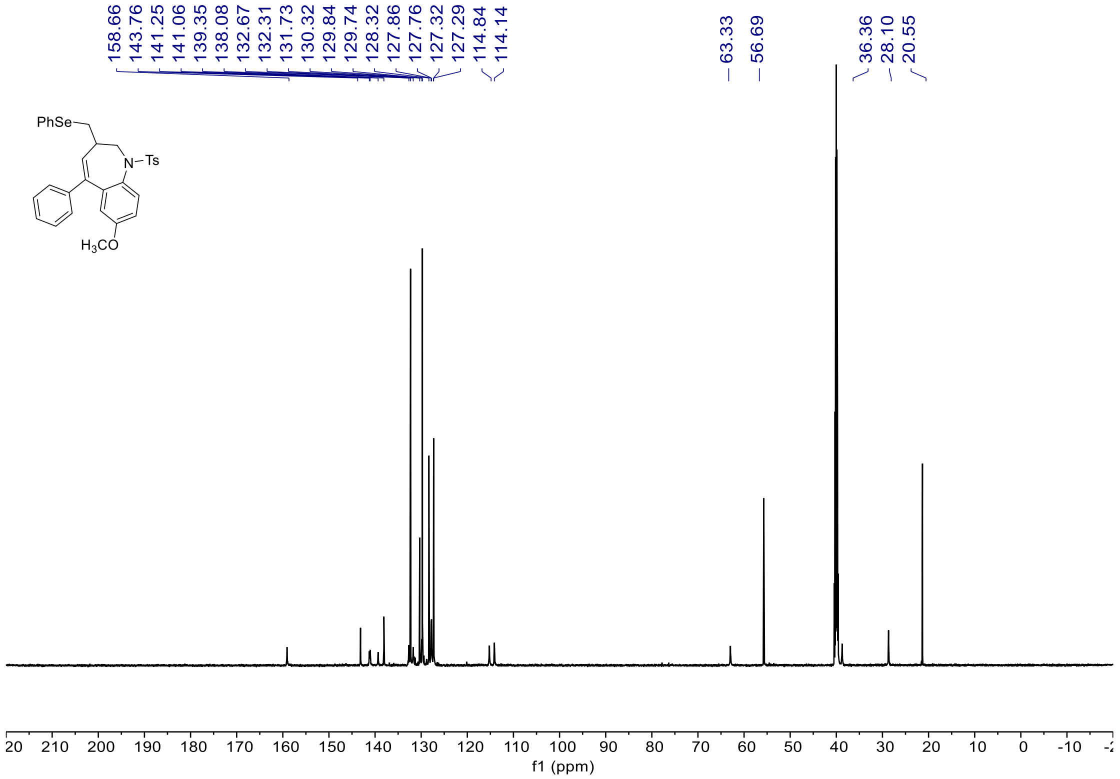

^{1}H NMR (600 MHz, DMSO-d_6) of compound **3j**

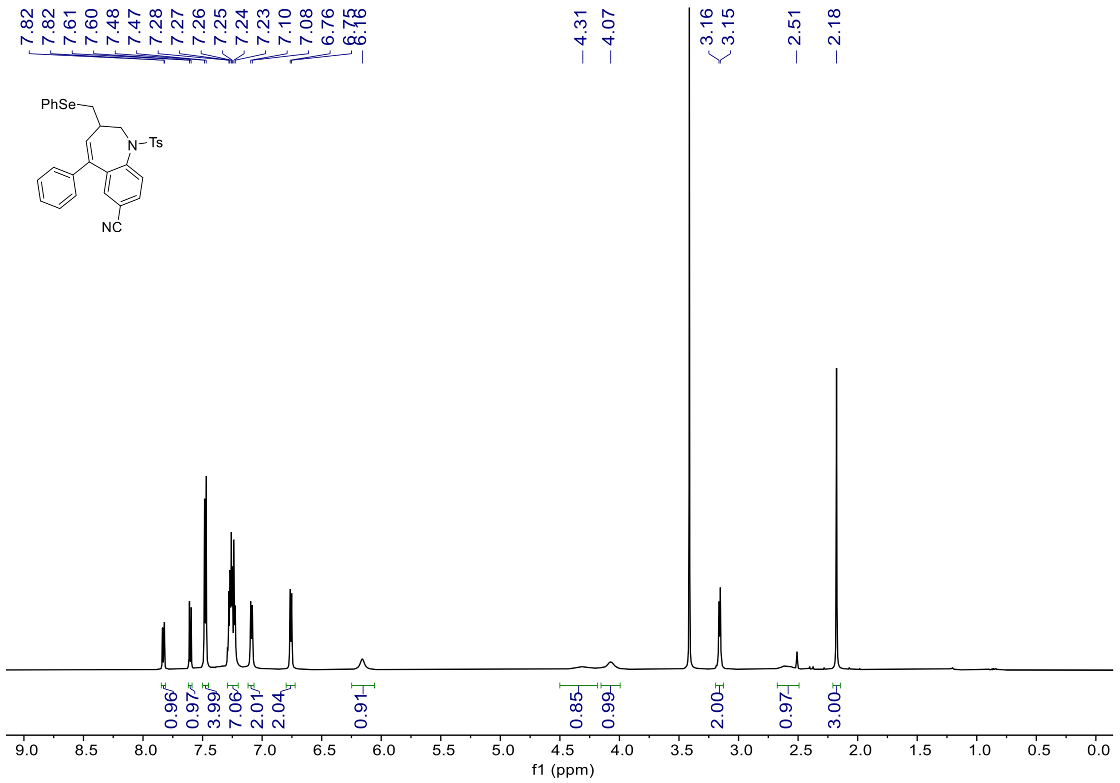

^{13}C NMR (151 MHz, DMSO-d_6) of compound **3j**

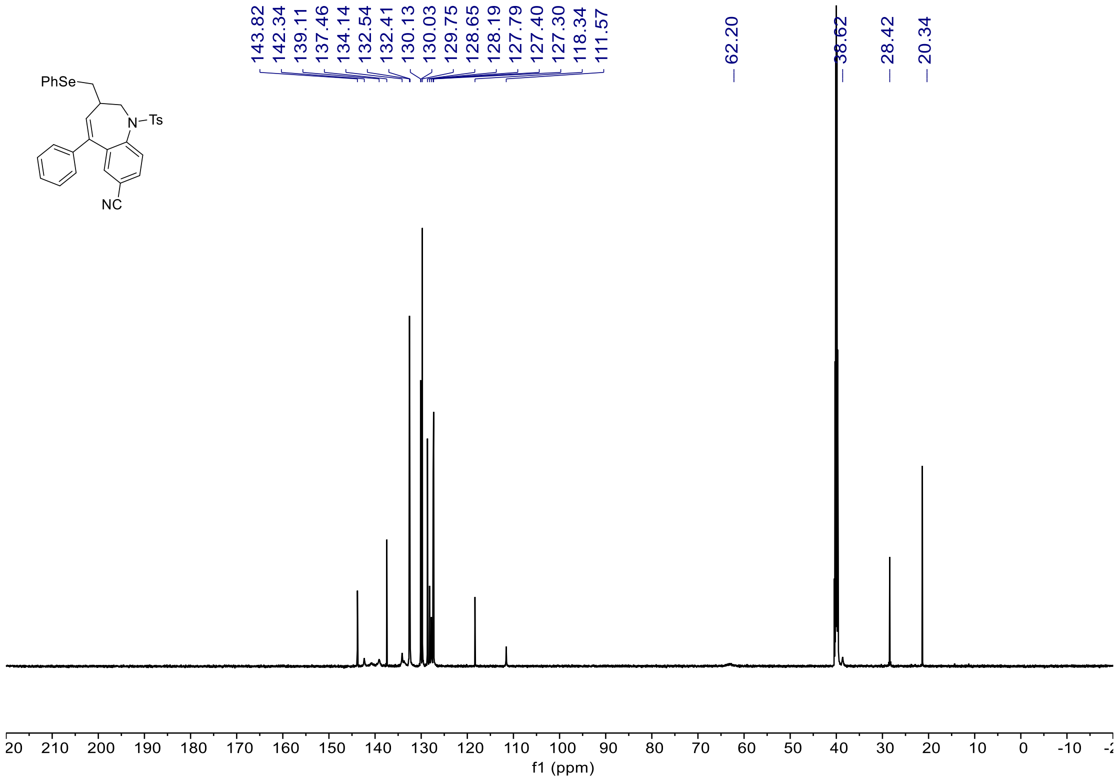

^{1}H NMR (600 MHz, DMSO-d_6) of compound **3k**

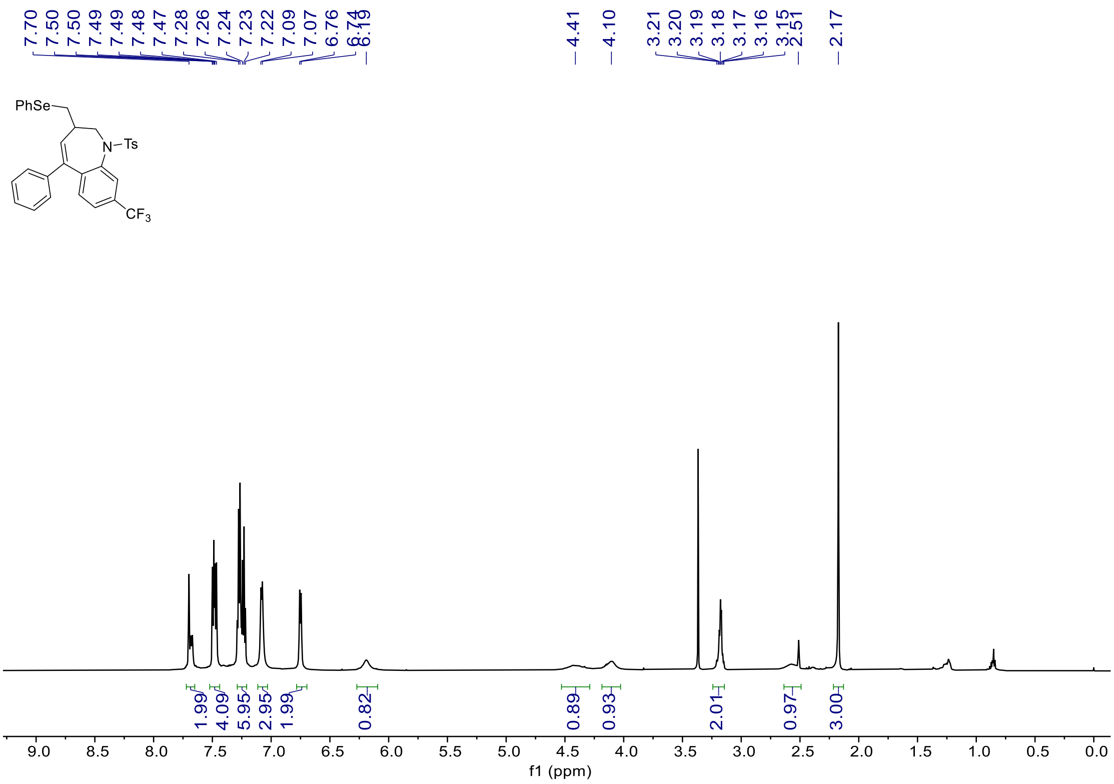

^{13}C NMR (151 MHz, DMSO-d_6) of compound **3k**

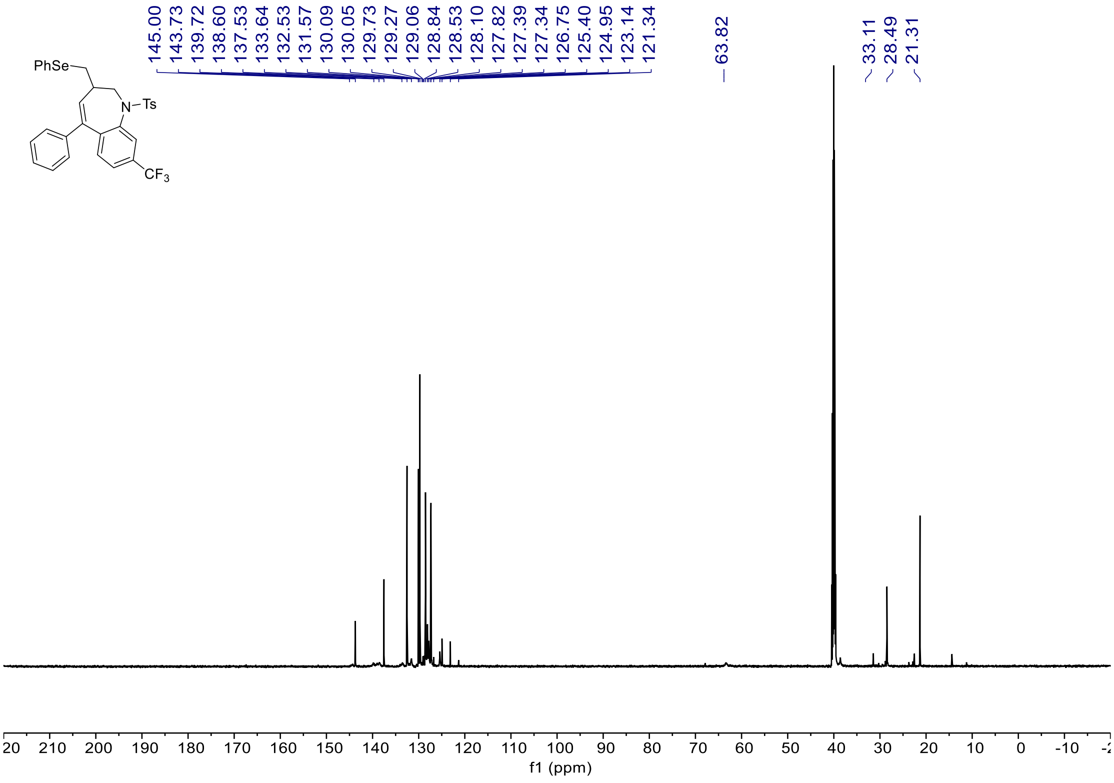

^{19}F NMR (565 MHz, DMSO-d_6) of compound **3k**

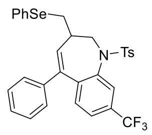

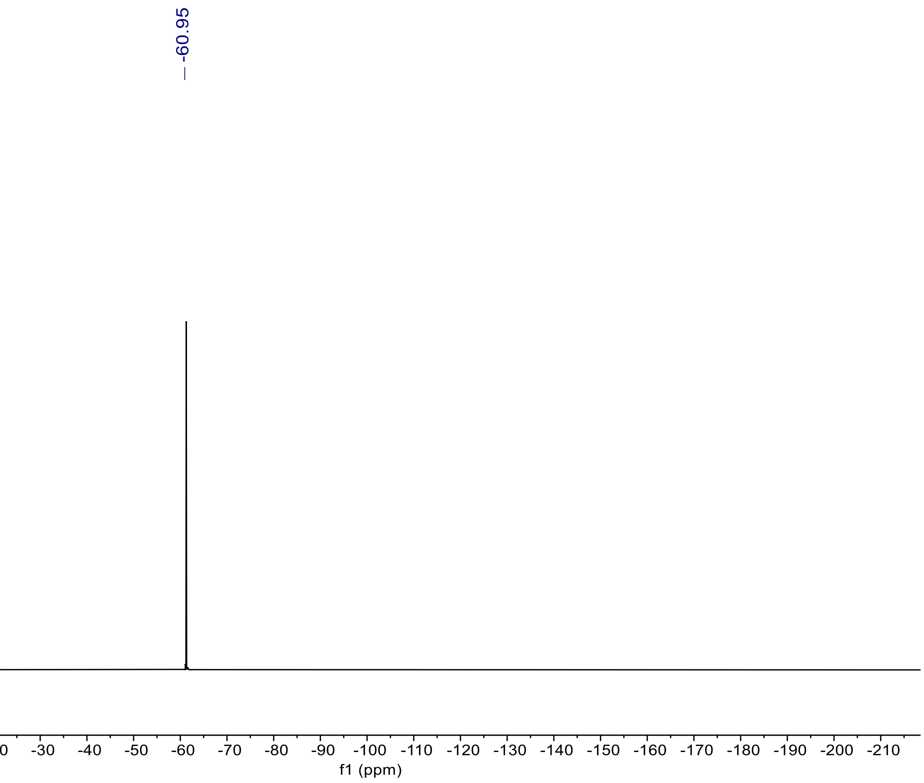

^{1}H NMR (600 MHz, DMSO-d_6) of compound **3l**

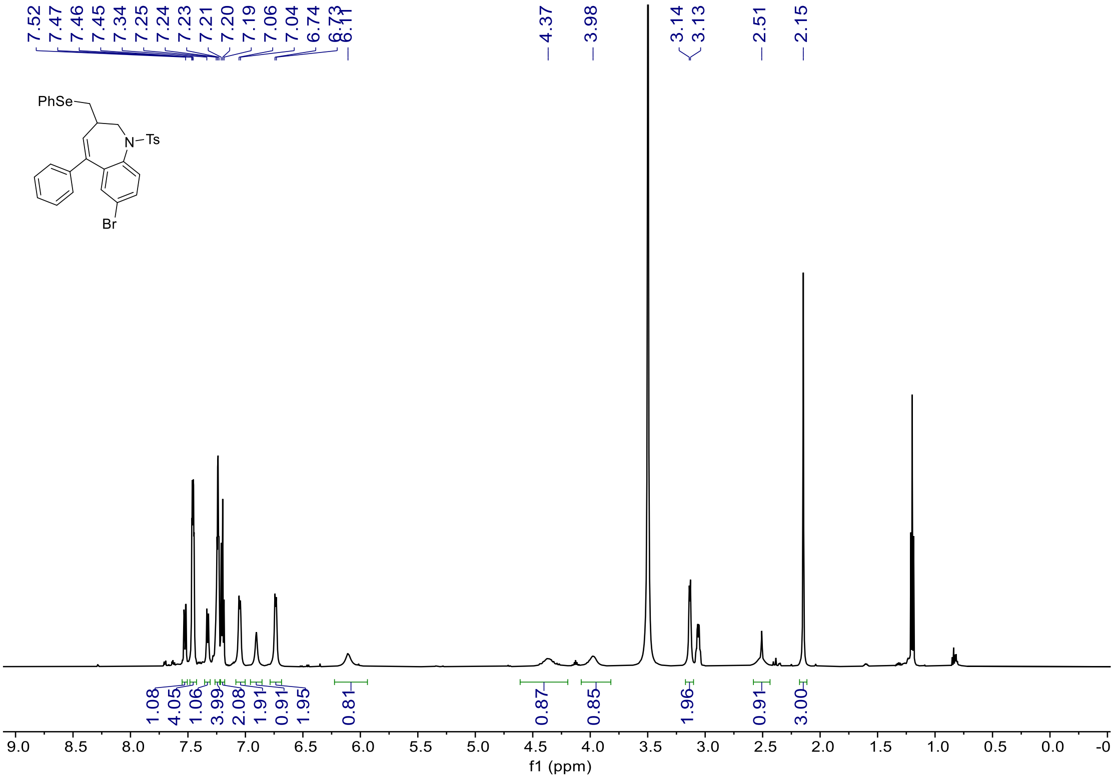

^{13}C NMR (151 MHz, DMSO-d_6) of compound **3l**

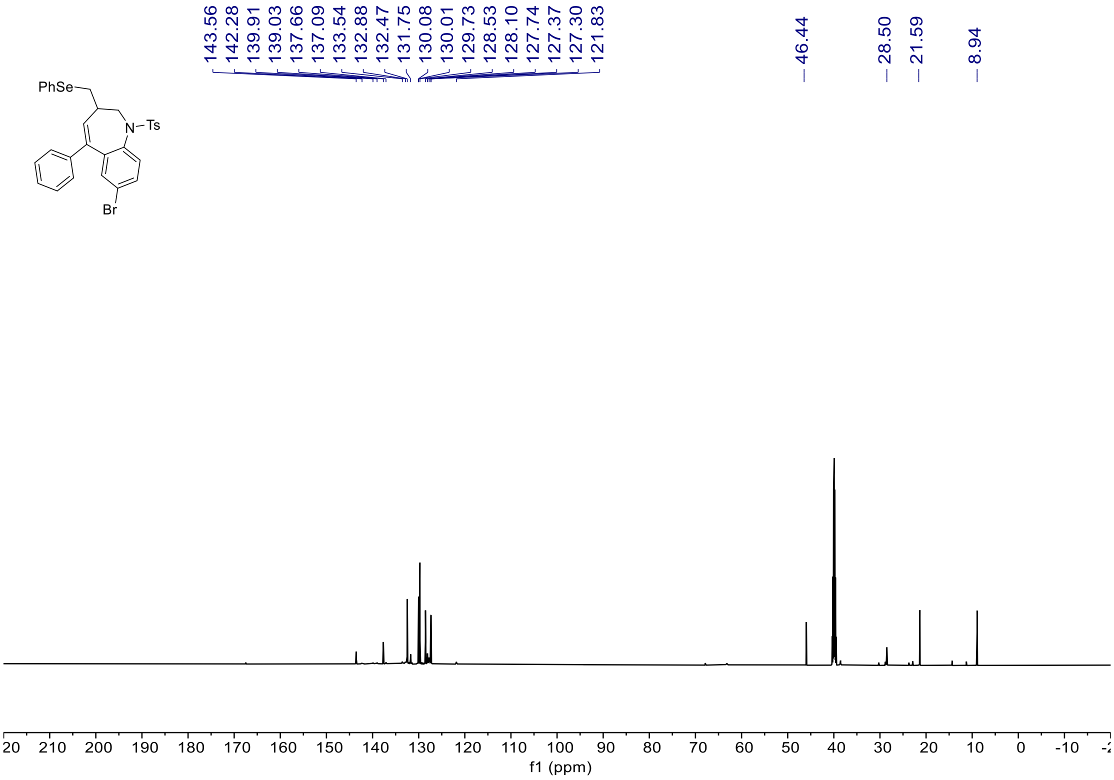

^{1}H NMR (600 MHz, DMSO-d_6) of compound **3m**

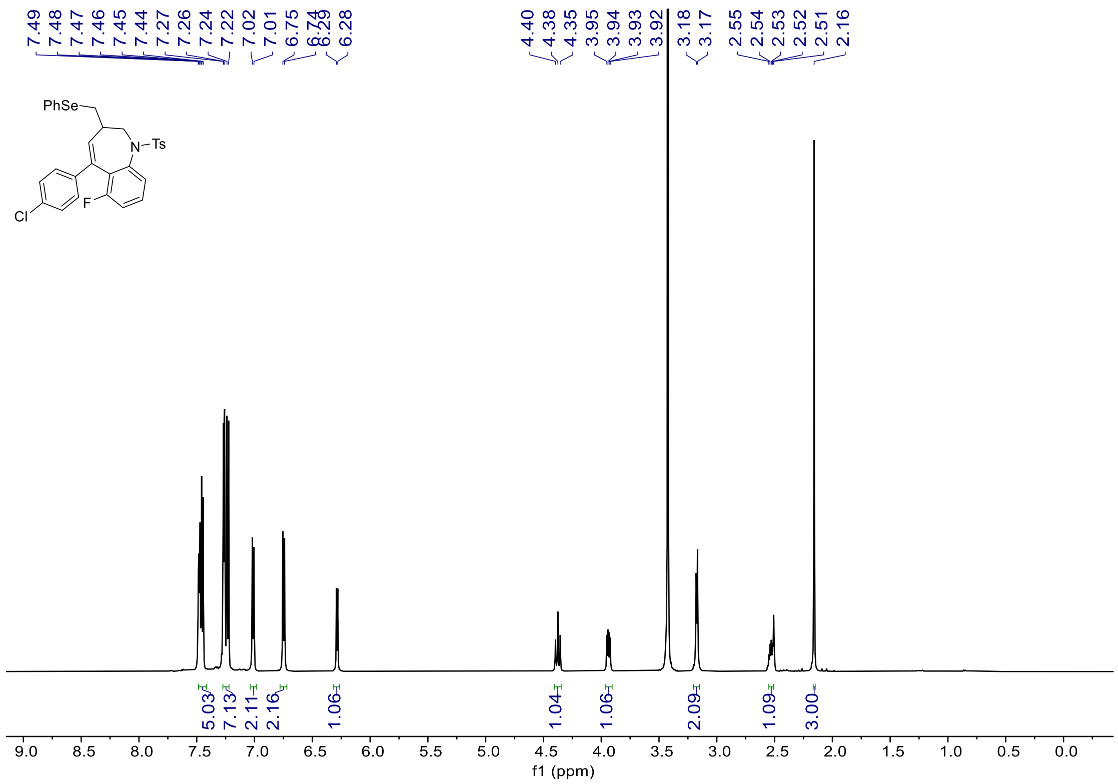

^{13}C NMR (151 MHz, DMSO-d_6) of compound **3m**

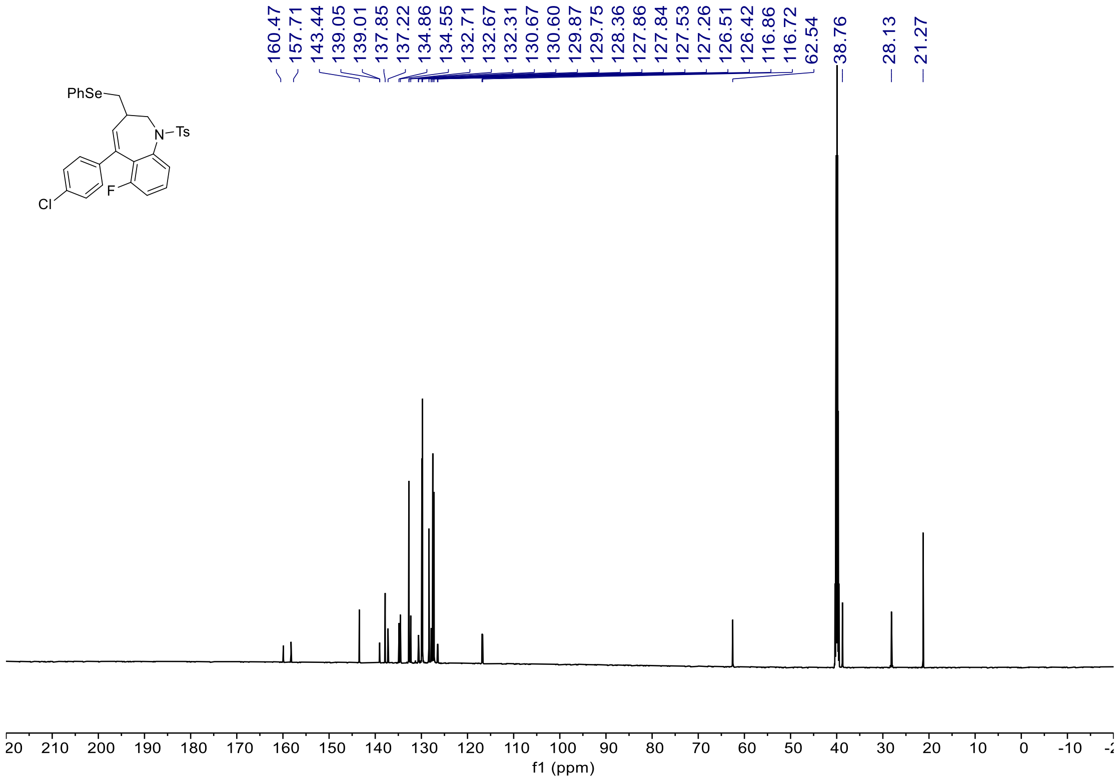

^{19}F NMR (565 MHz, DMSO-d_6) of compound **3m**

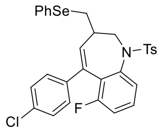

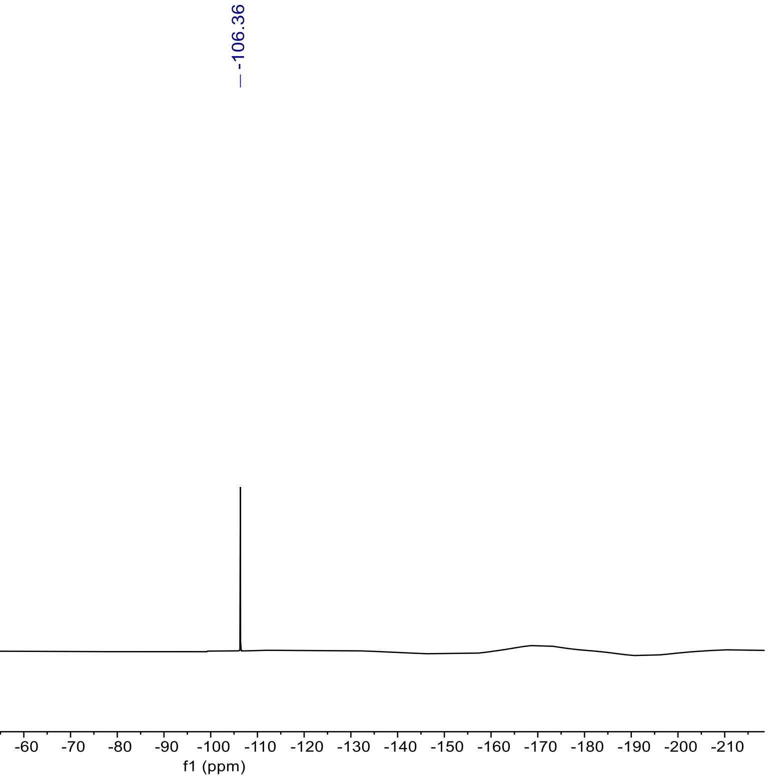

^{1}H NMR (600 MHz, DMSO-d_6) of compound **3n**

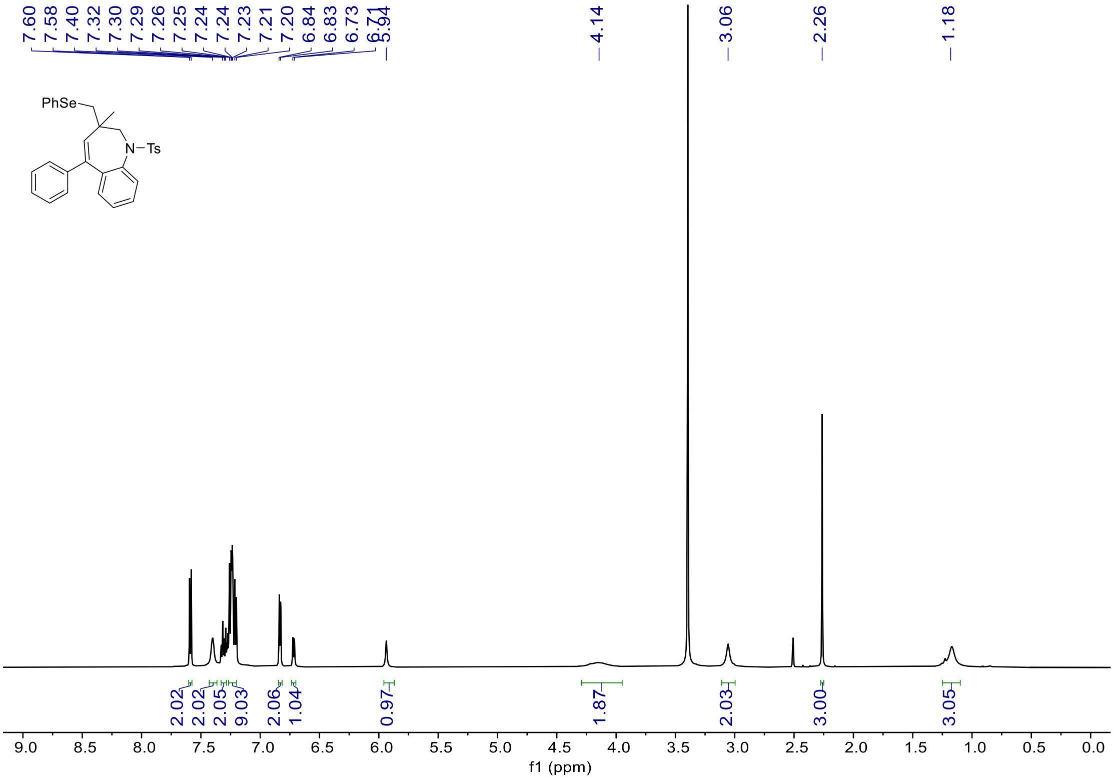

^{13}C NMR (151 MHz, DMSO-d_6) of compound **3n**

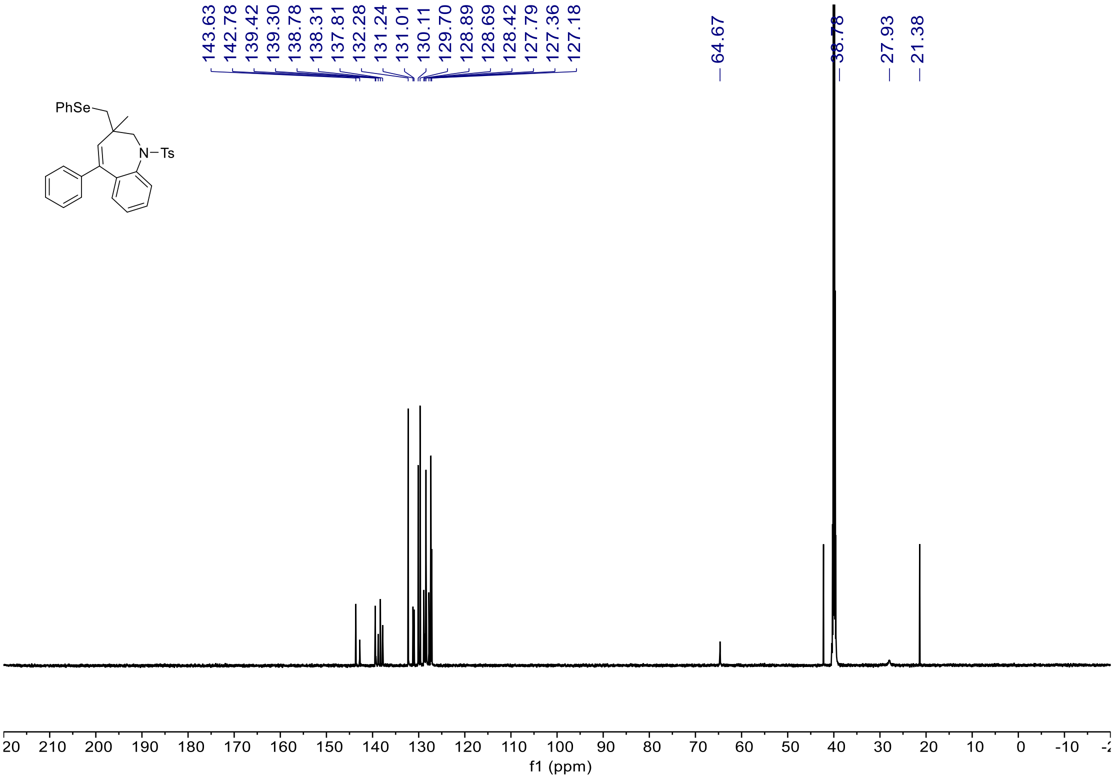

^{1}H NMR (600 MHz, DMSO-d_6) of compound **3o**

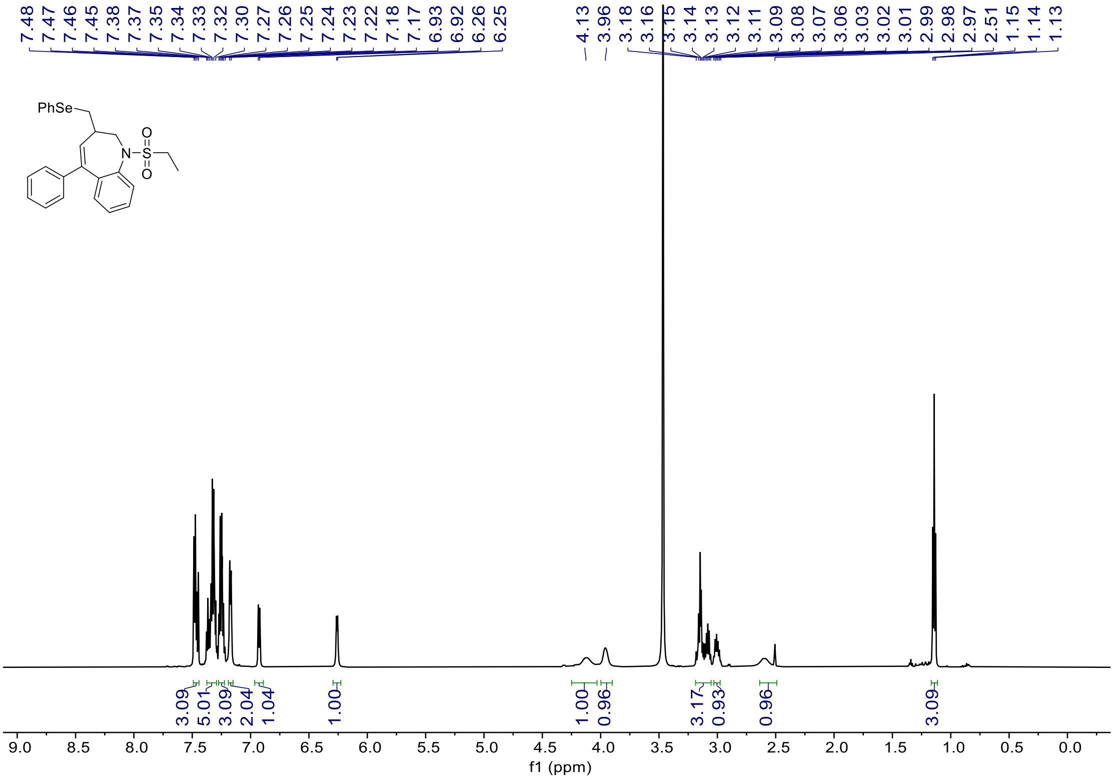

^{13}C NMR (151 MHz, DMSO-d_6) of compound **3o**

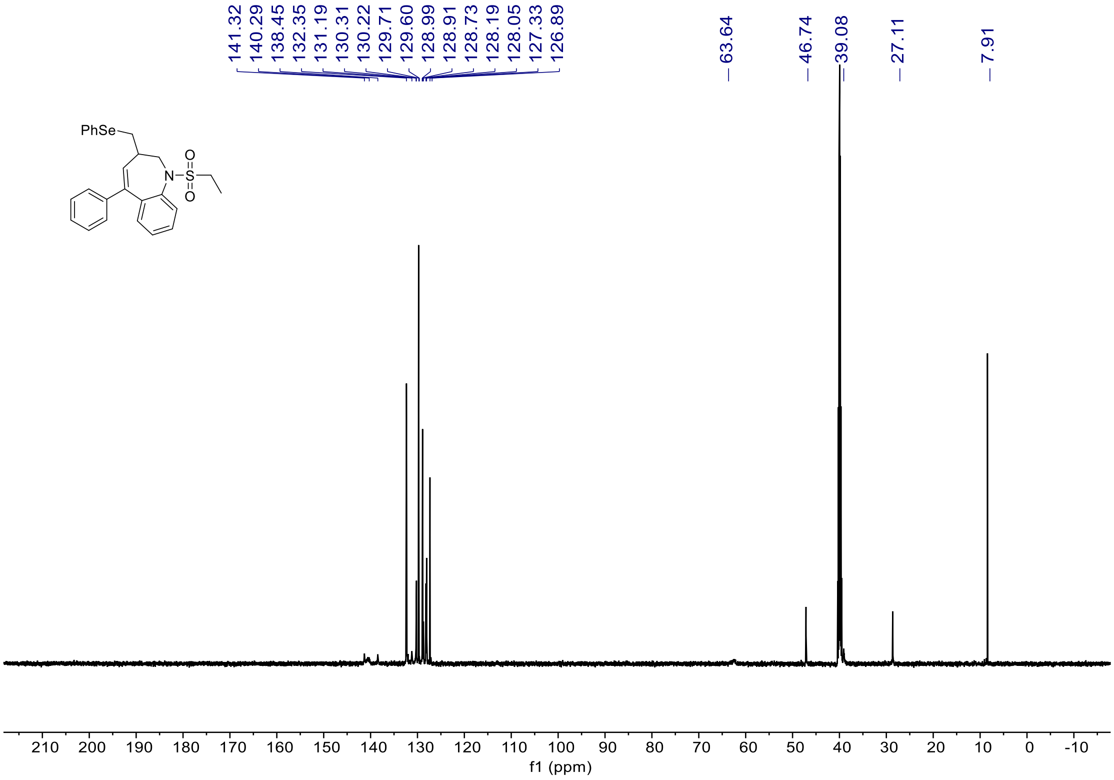

^{1}H NMR (600 MHz, DMSO-d_6) of compound **3p**

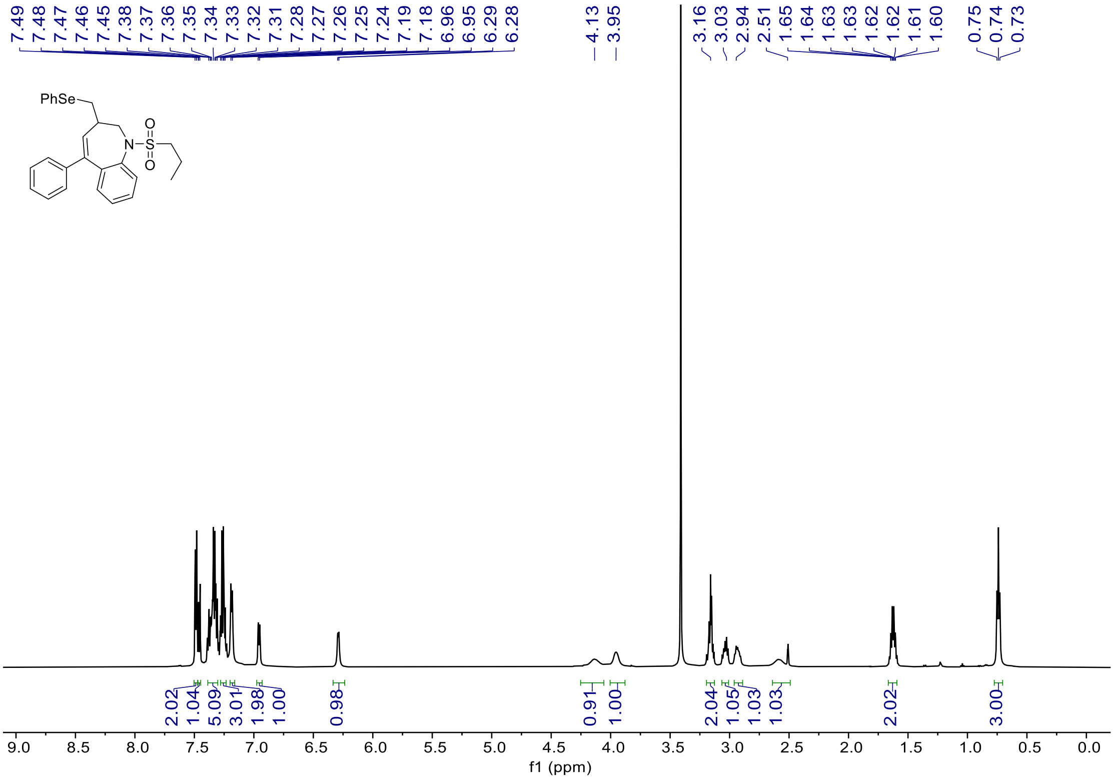

^{13}C NMR (151 MHz, DMSO-d_6) of compound **3p**

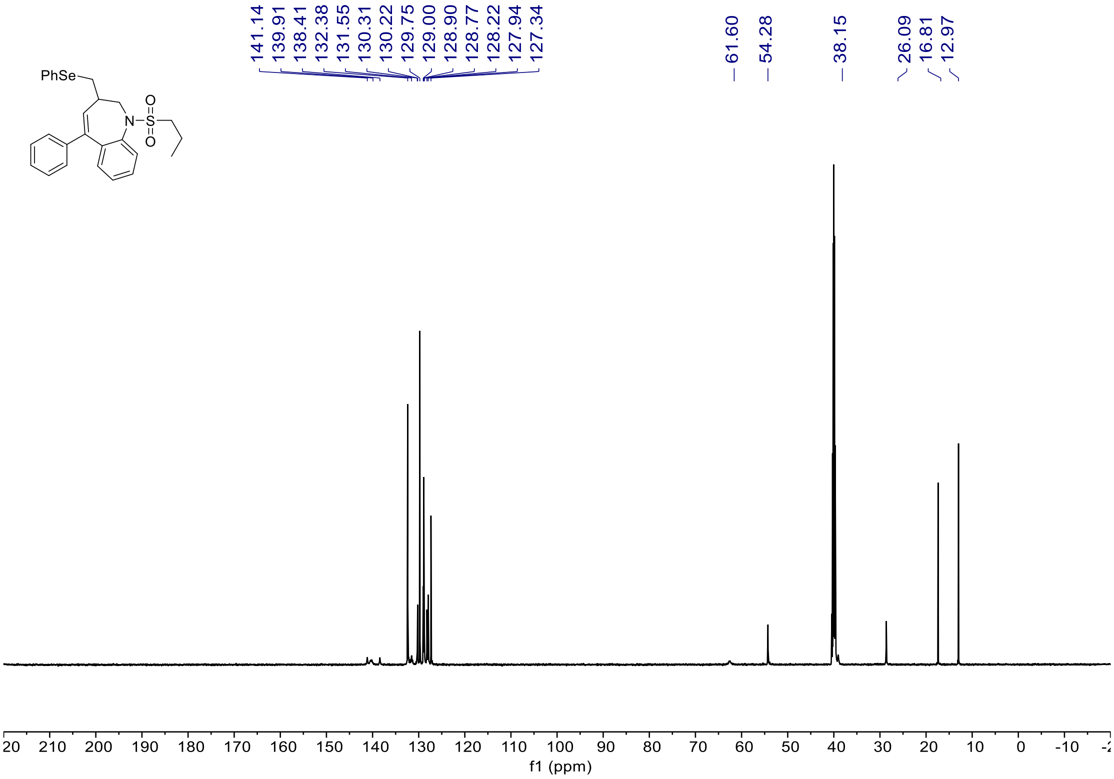

^{1}H NMR (600 MHz, DMSO-d_6) of compound **3q**

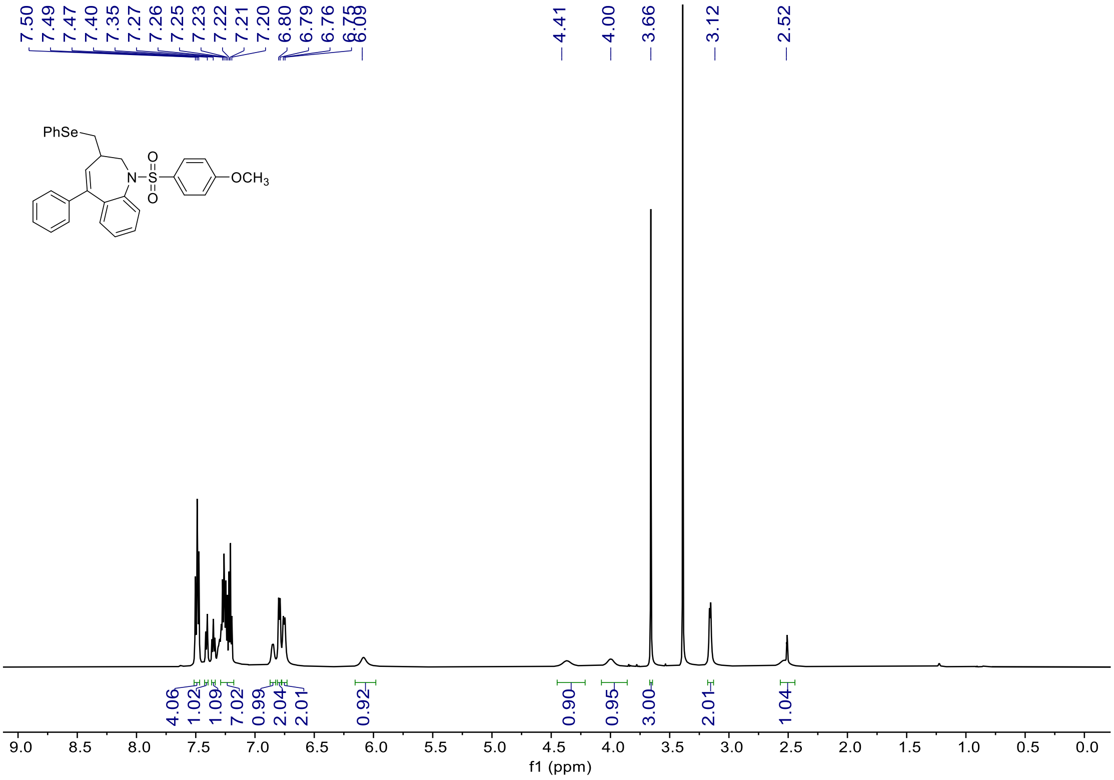

^{13}C NMR (151 MHz, DMSO-d_6) of compound **3q**

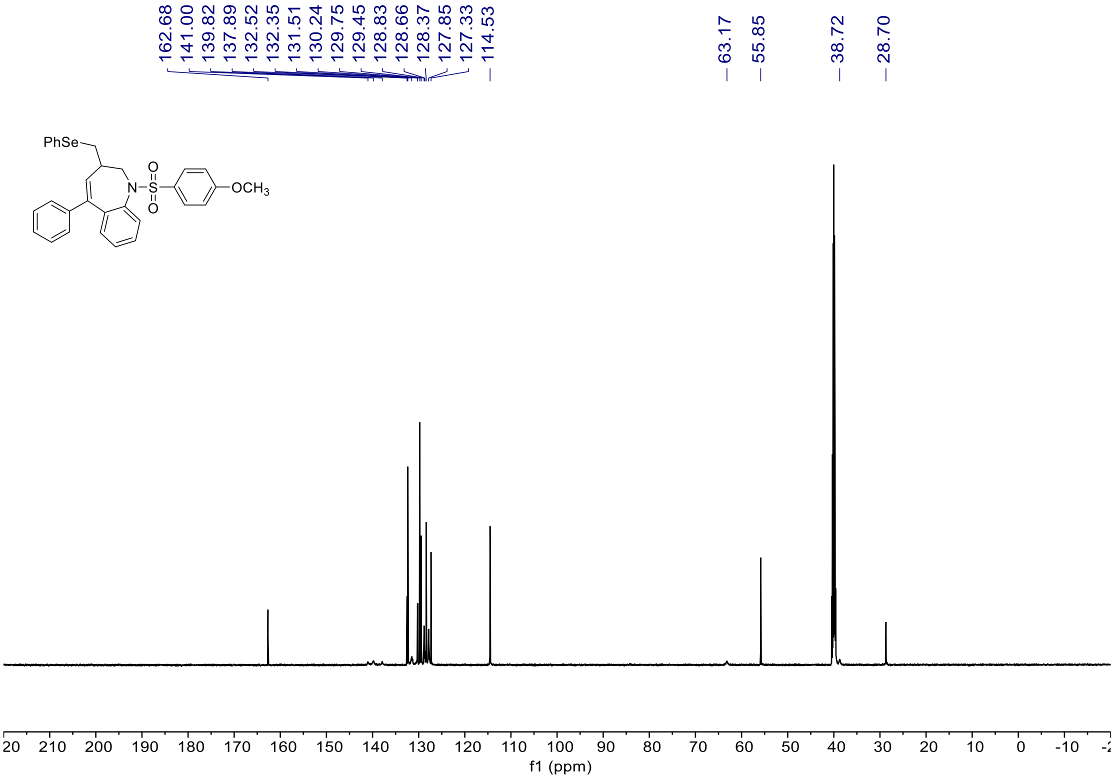

^{1}H NMR (600 MHz, DMSO-d_6) of compound **3r**

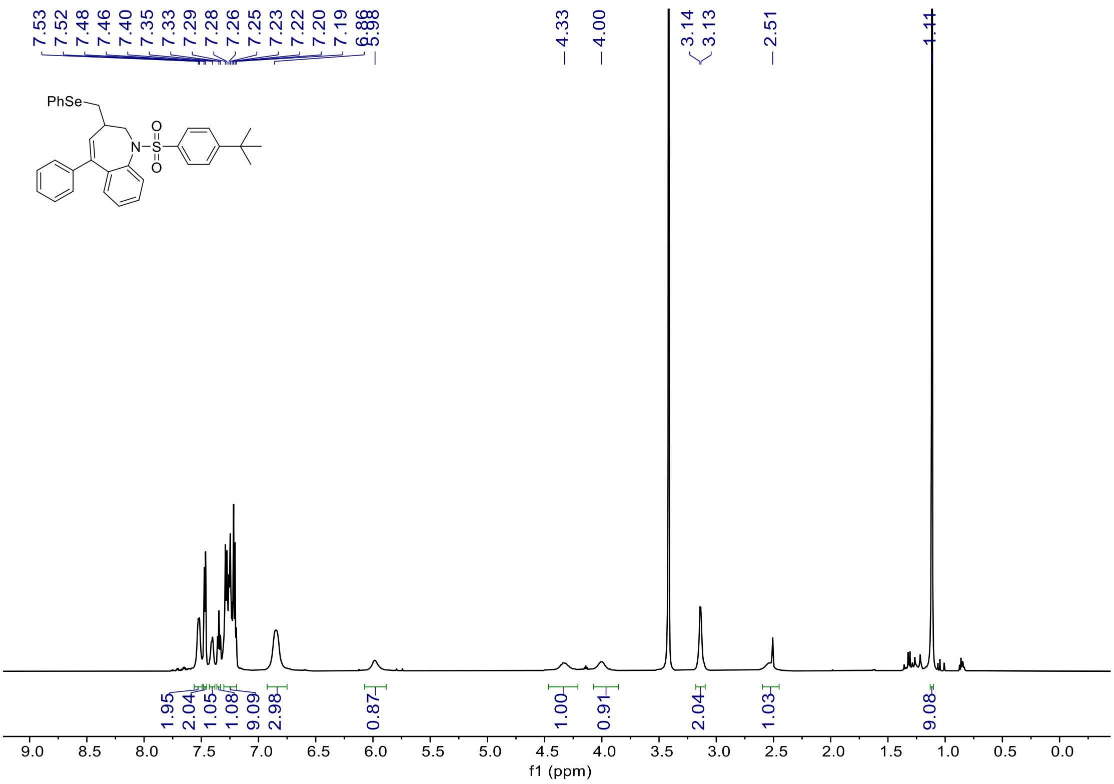

^{13}C NMR (151 MHz, DMSO-d_6) of compound **3r**

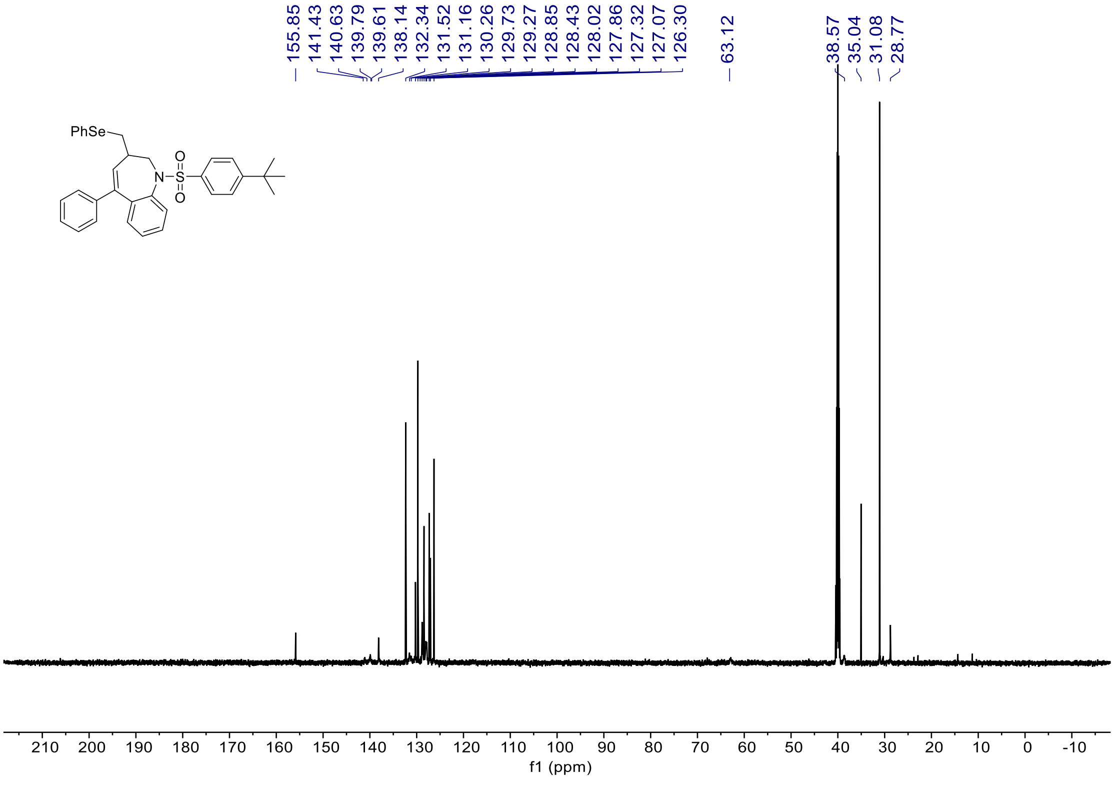

^{1}H NMR (600 MHz, DMSO-d_6) of compound **3s**

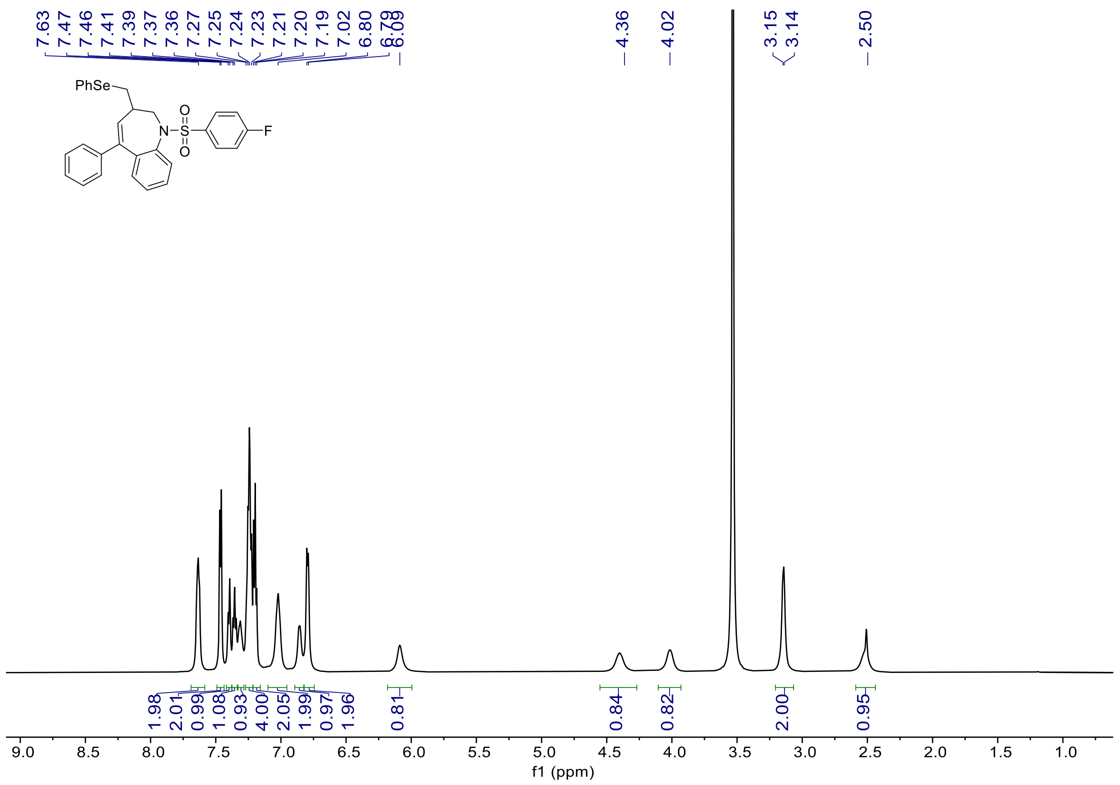

^{13}C NMR (151 MHz, DMSO-d_6) of compound **3s**

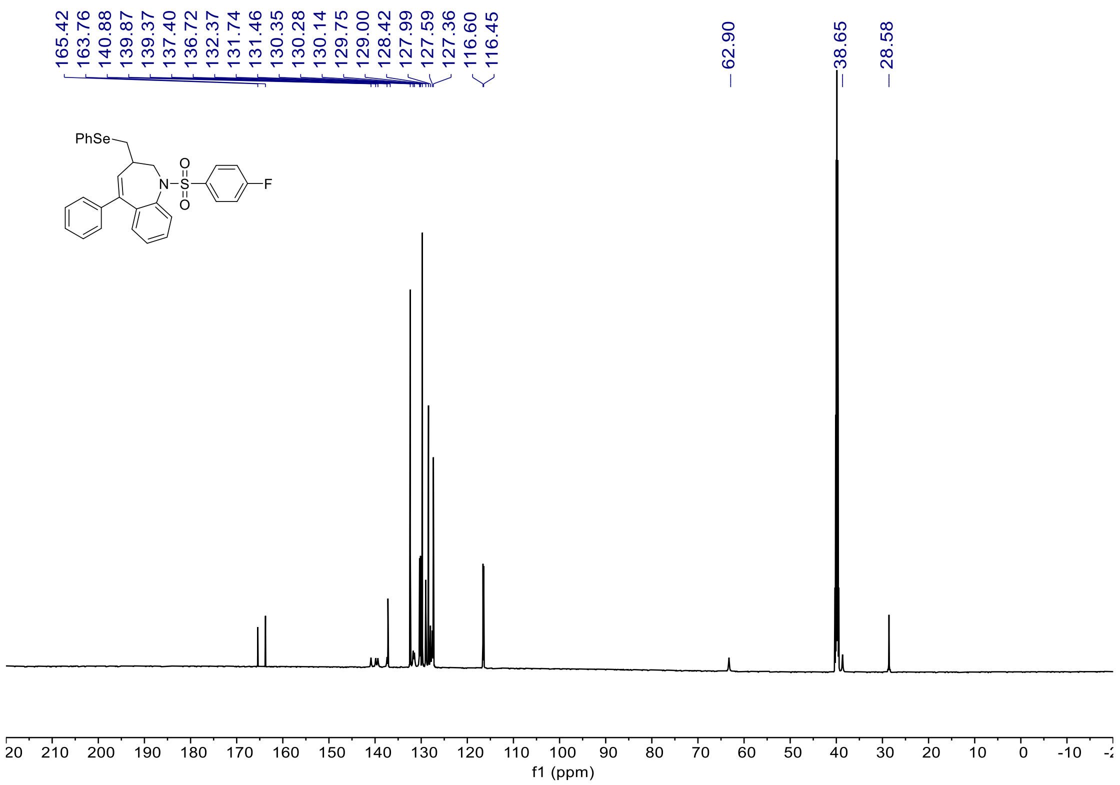

^{19}F NMR (565 MHz, DMSO-d_6) of compound **3s**

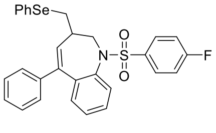

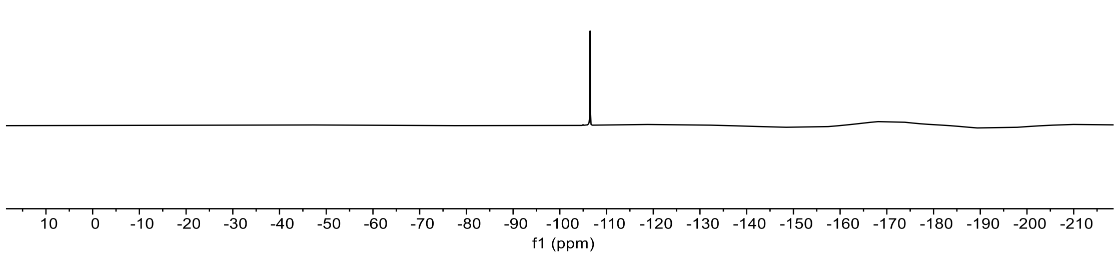

^{1}H NMR (600 MHz, DMSO-d_6) of compound **3t**

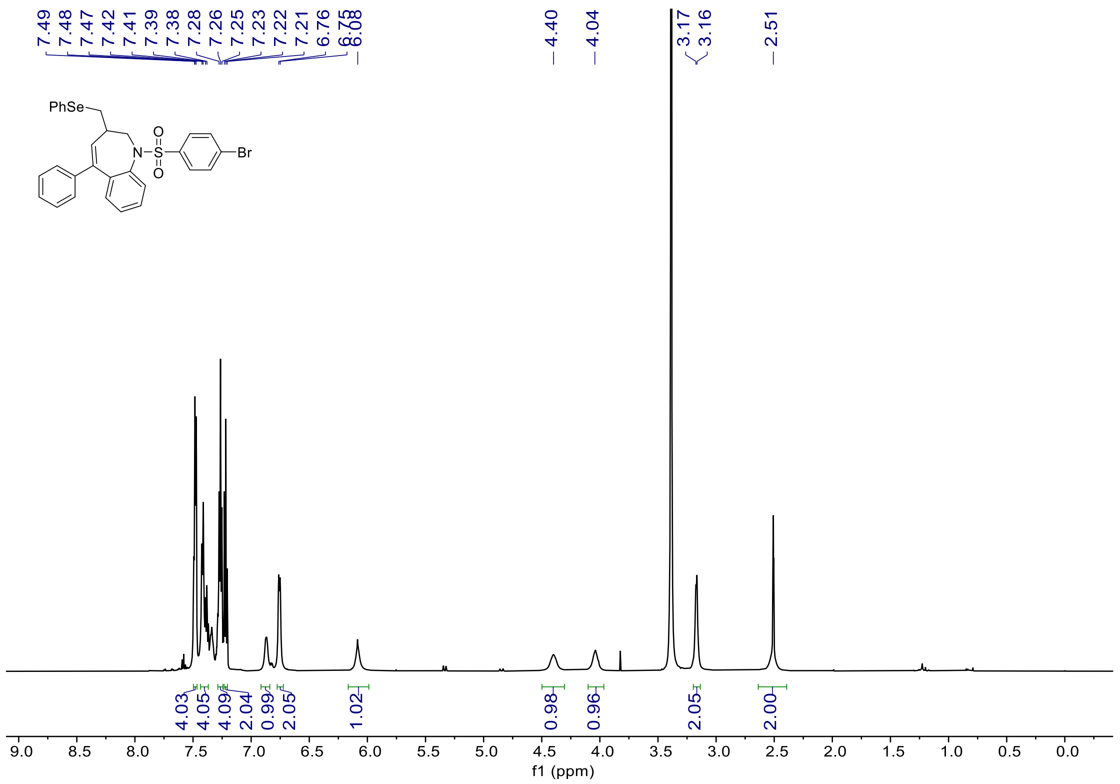

^{13}C NMR (151 MHz, DMSO-d_6) of compound **3t**

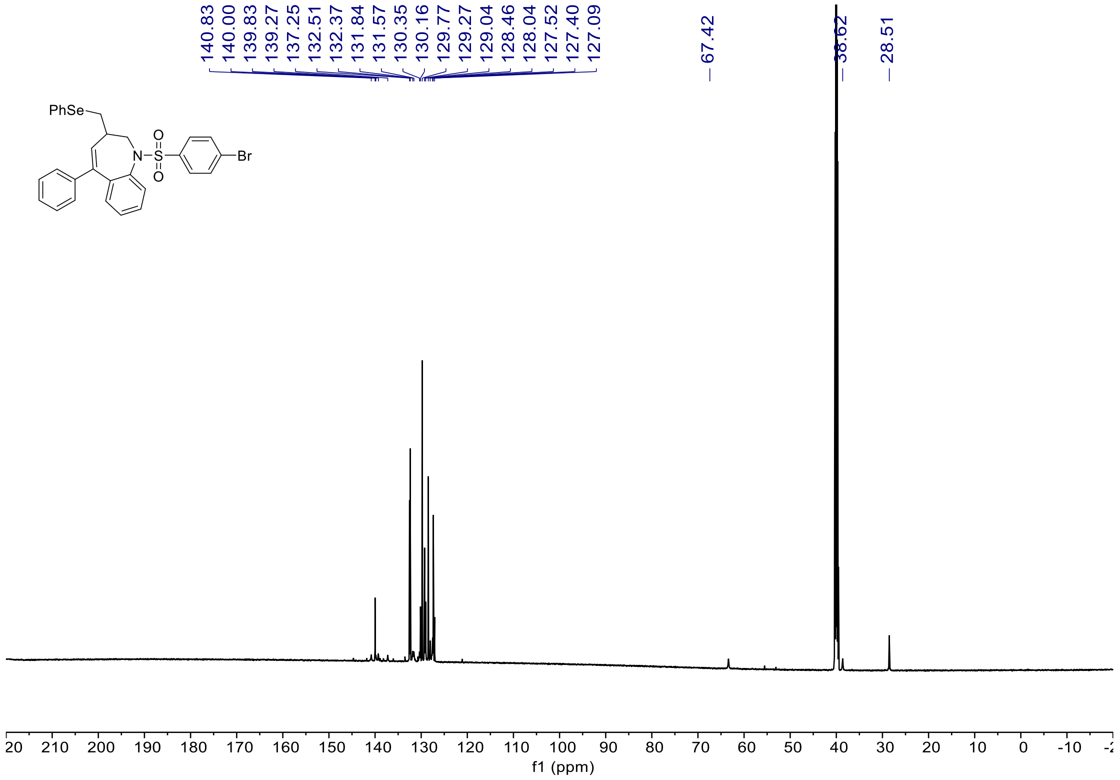

^{1}H NMR (600 MHz, DMSO-d_6) of compound **3u**

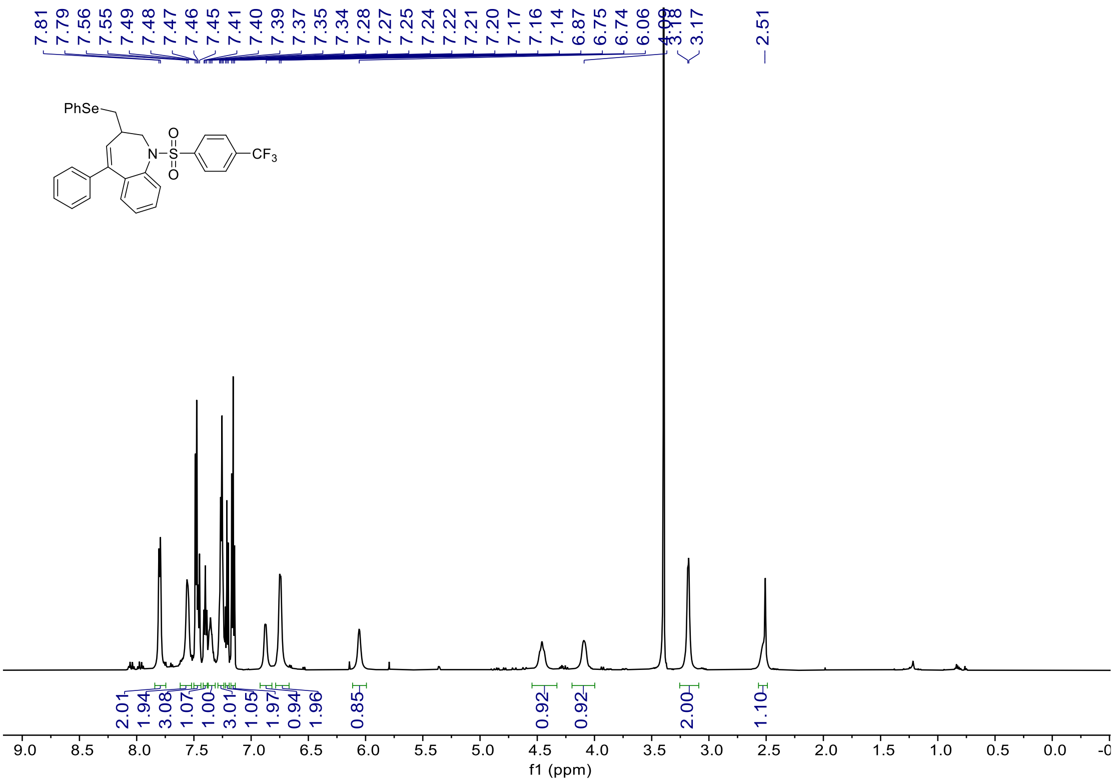

^{13}C NMR (151 MHz, DMSO-d_6) of compound **3u**

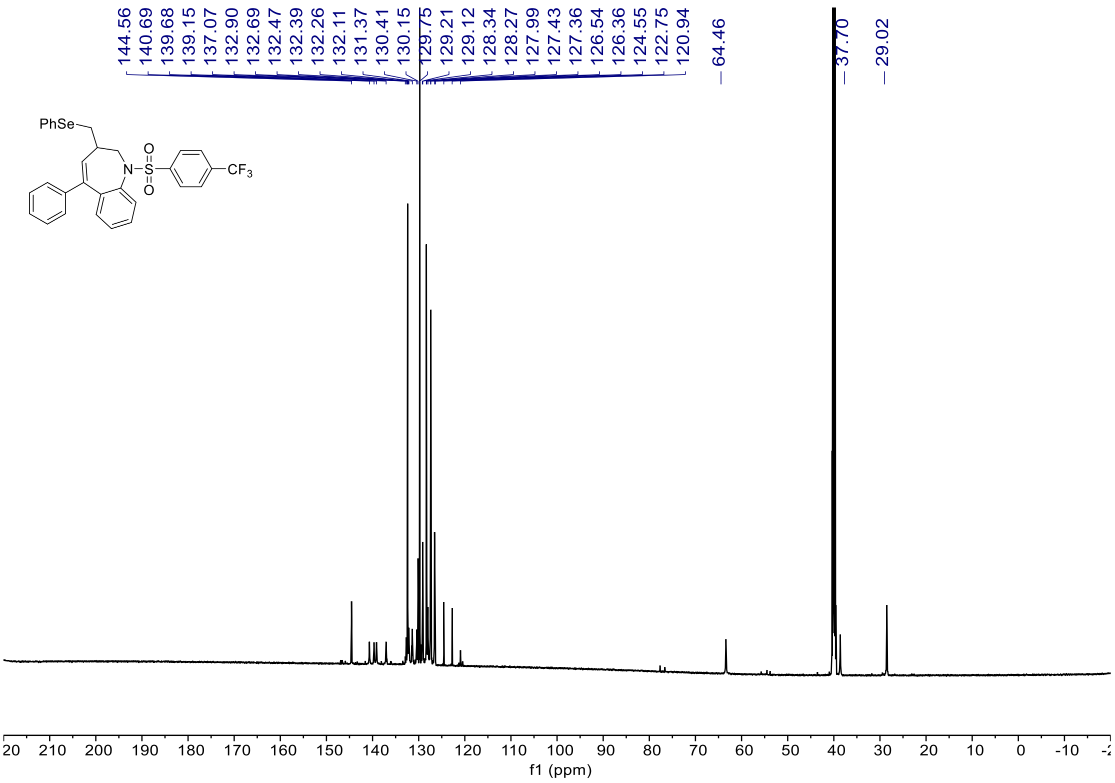

^{19}F NMR (565 MHz, DMSO-d_6) of compound **3u**

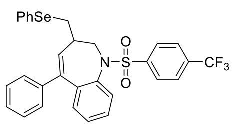

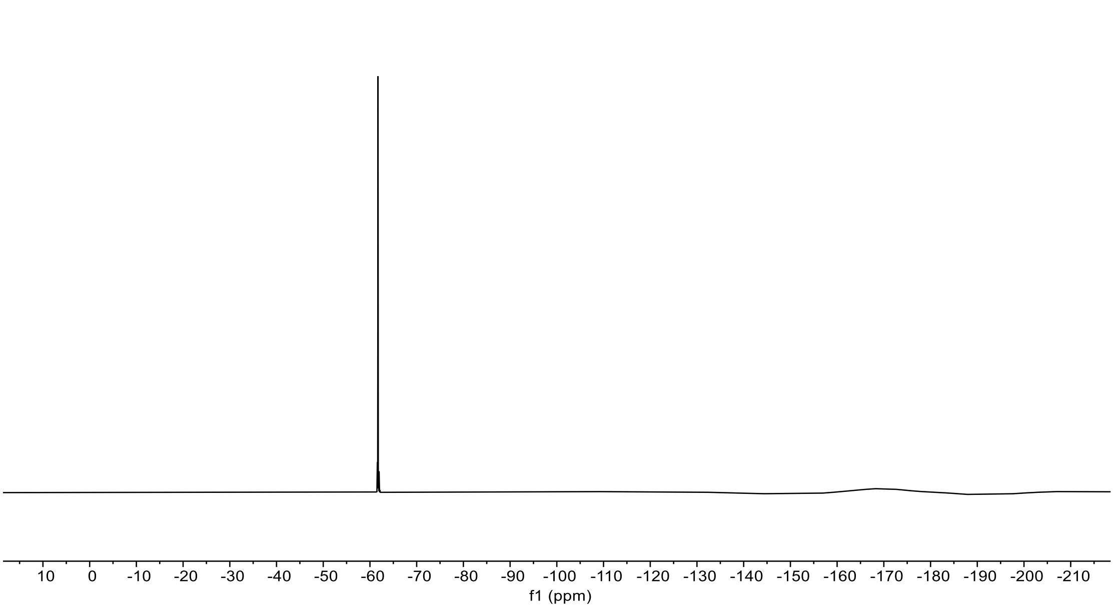

^{1}H NMR (600 MHz, DMSO-d_6) of compound **3v**

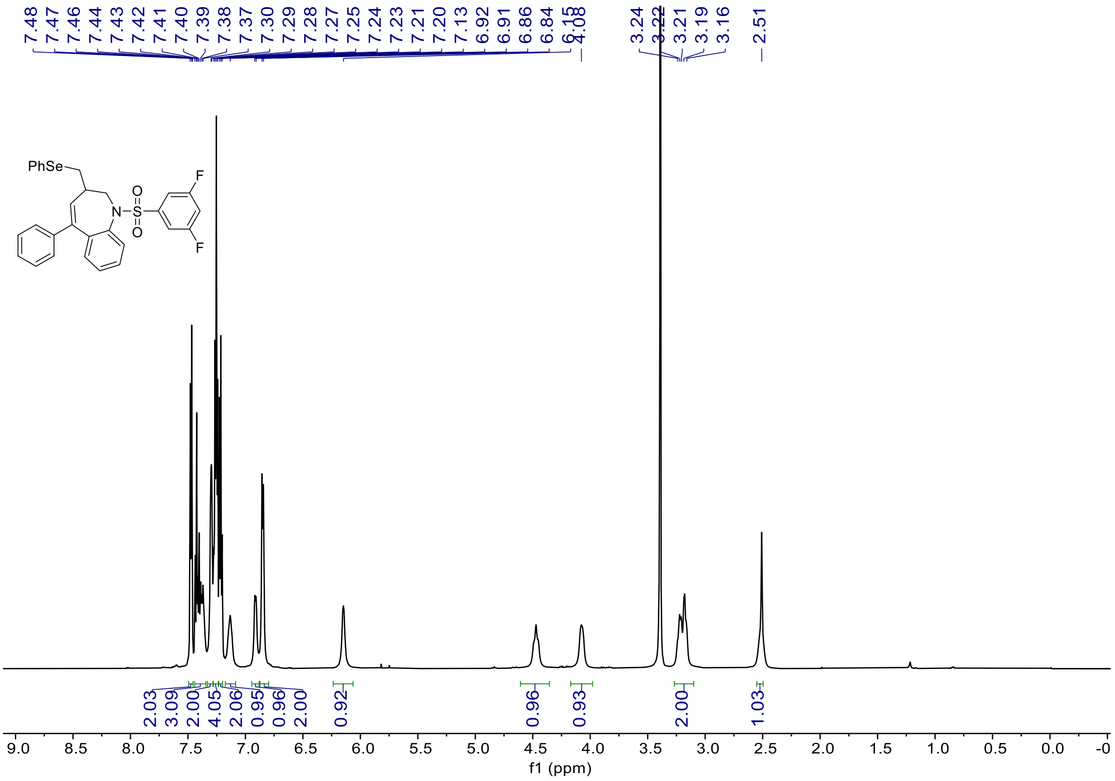

^{13}C NMR (151 MHz, DMSO-d_6) of compound **3v**

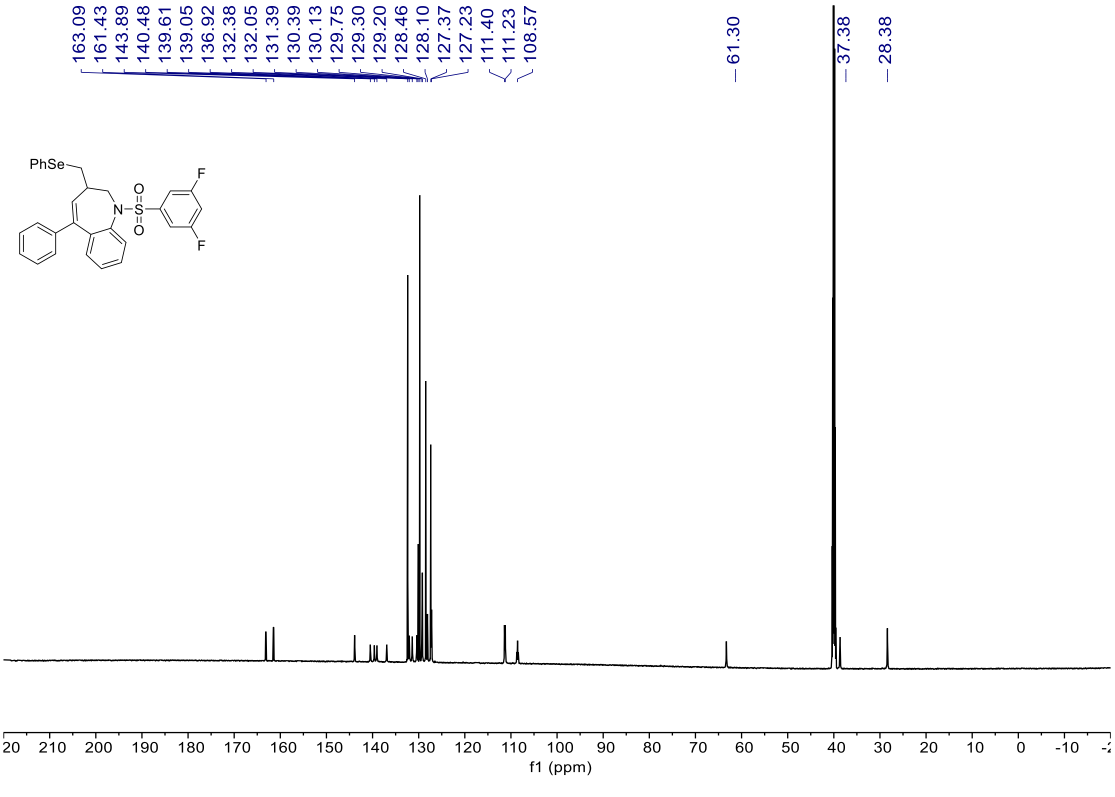

^{19}F NMR (565 MHz, DMSO-d_6) of compound **3v**

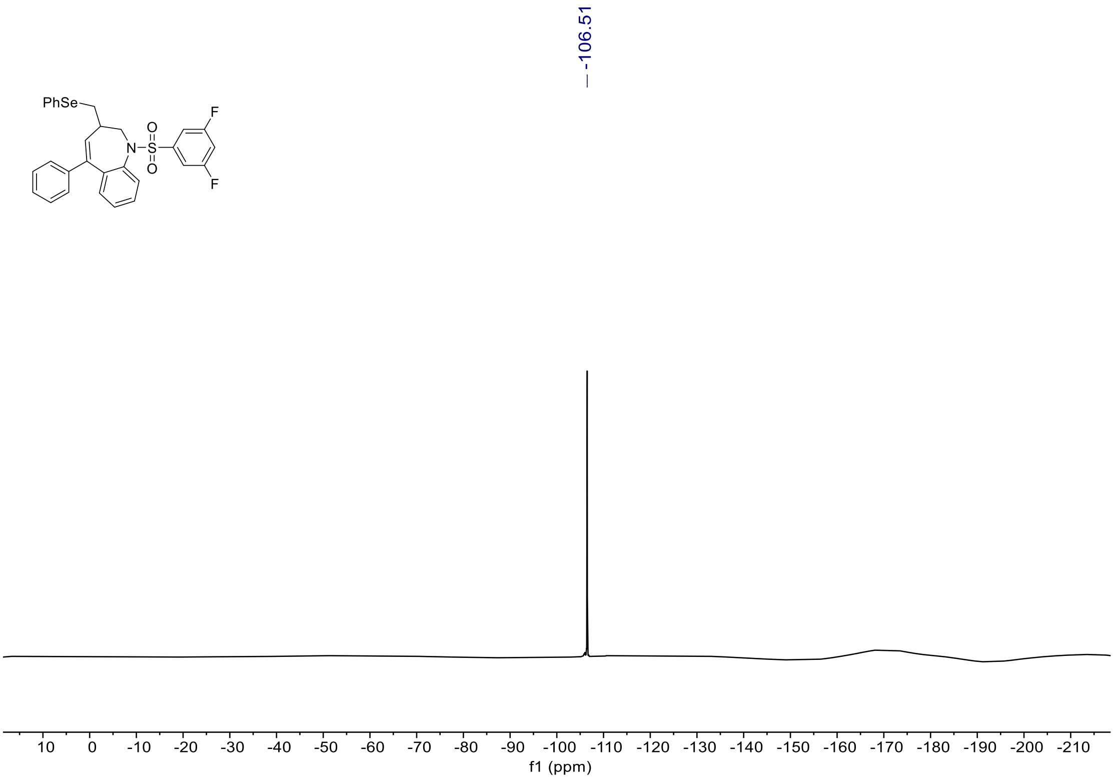

^{1}H NMR (600 MHz, DMSO-d_6) of compound **3w**

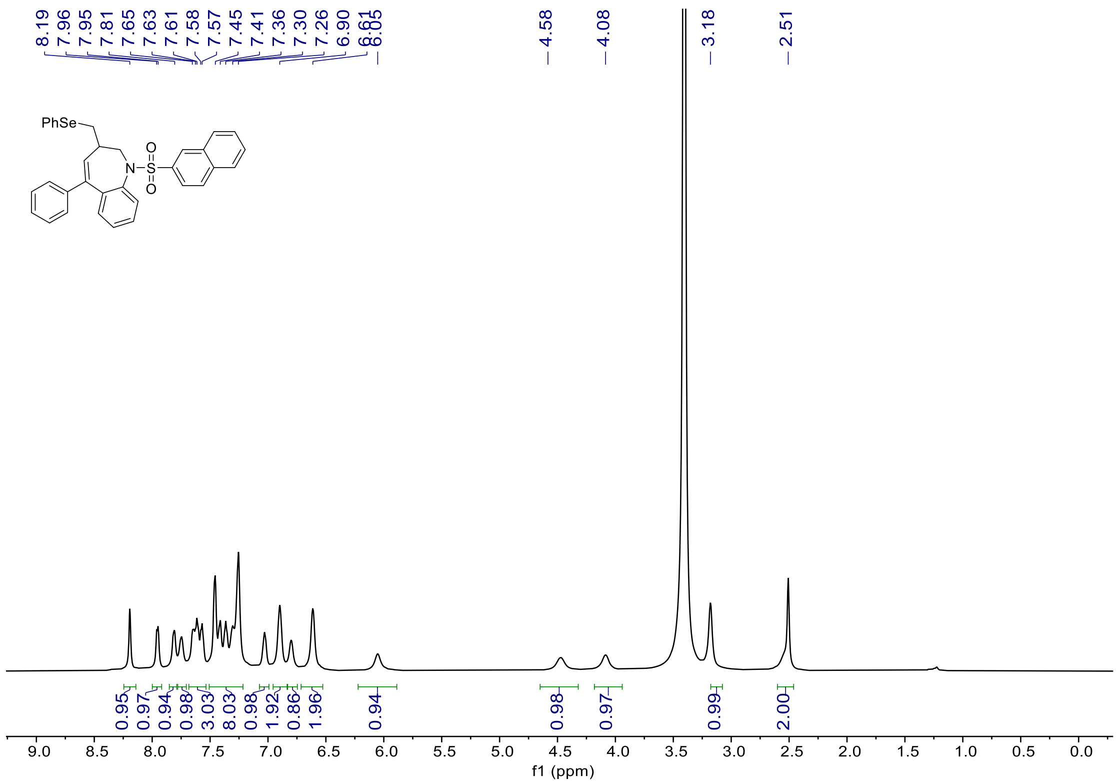

^{13}C NMR (151 MHz, DMSO-d_6) of compound **3w**

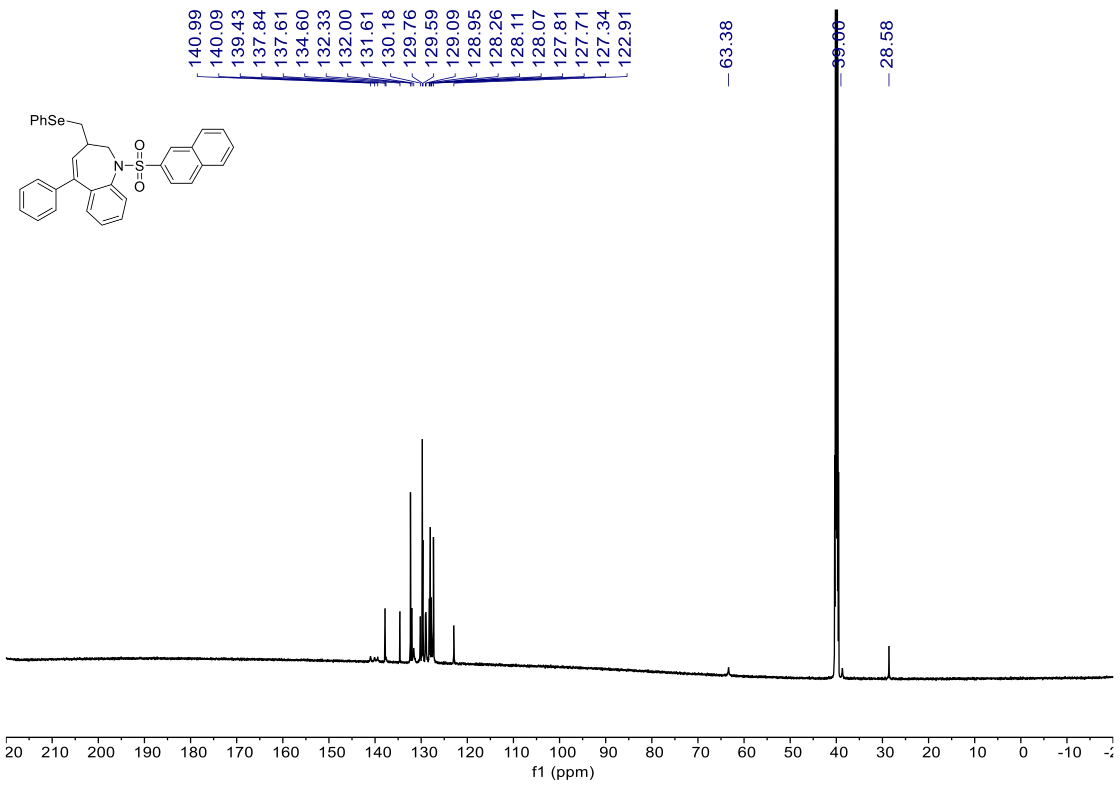

^{1}H NMR (600 MHz, DMSO-d_6) of compound **3x**

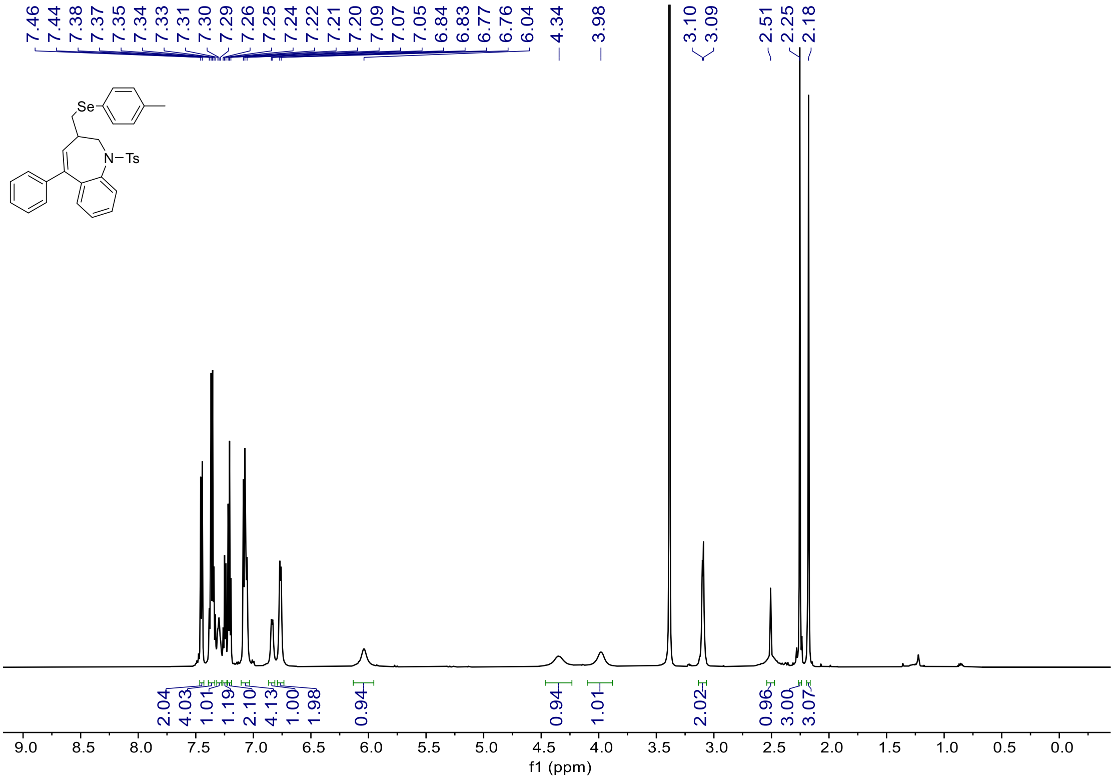

^{13}C NMR (151 MHz, DMSO-d_6) of compound **3x**

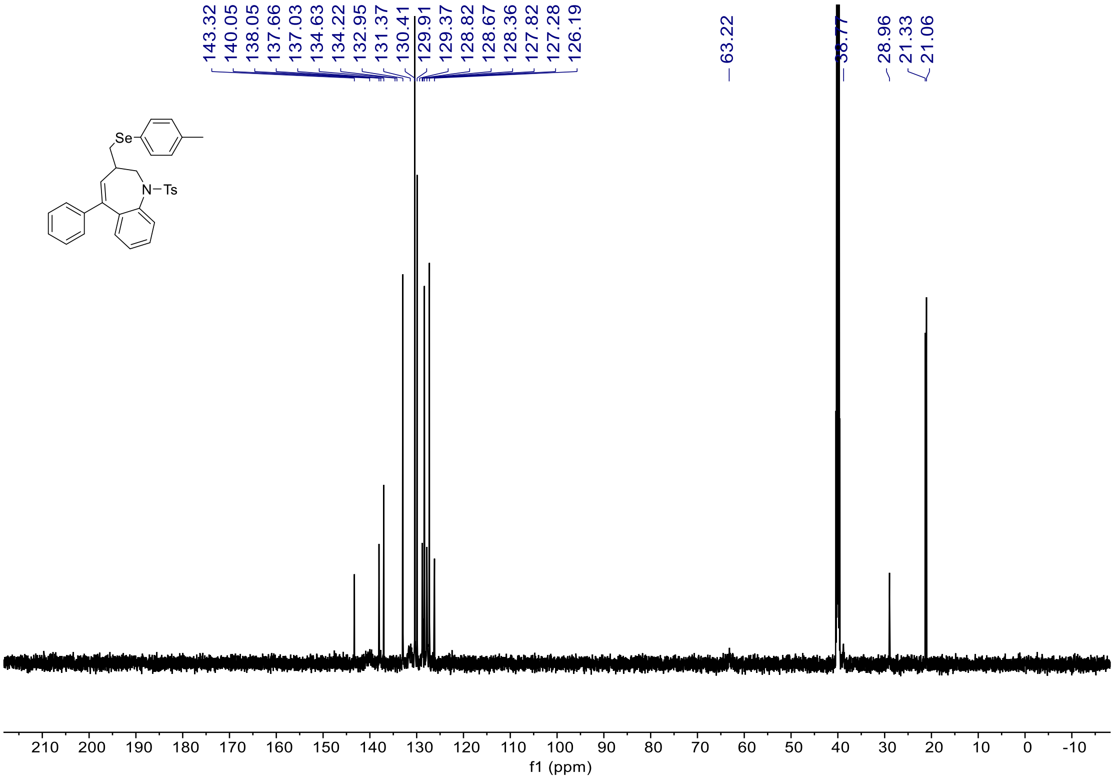

^{1}H NMR (600 MHz, DMSO-d_6) of compound **3y**

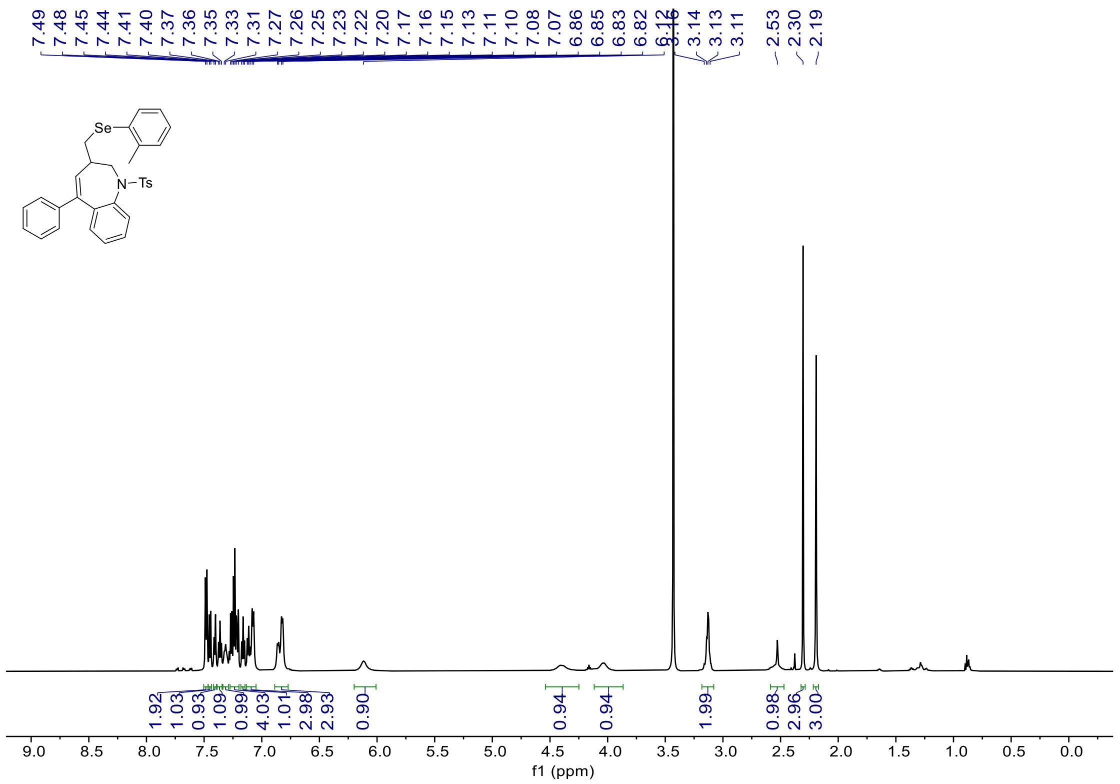

^{13}C NMR (151 MHz, DMSO-d_6) of compound **3y**

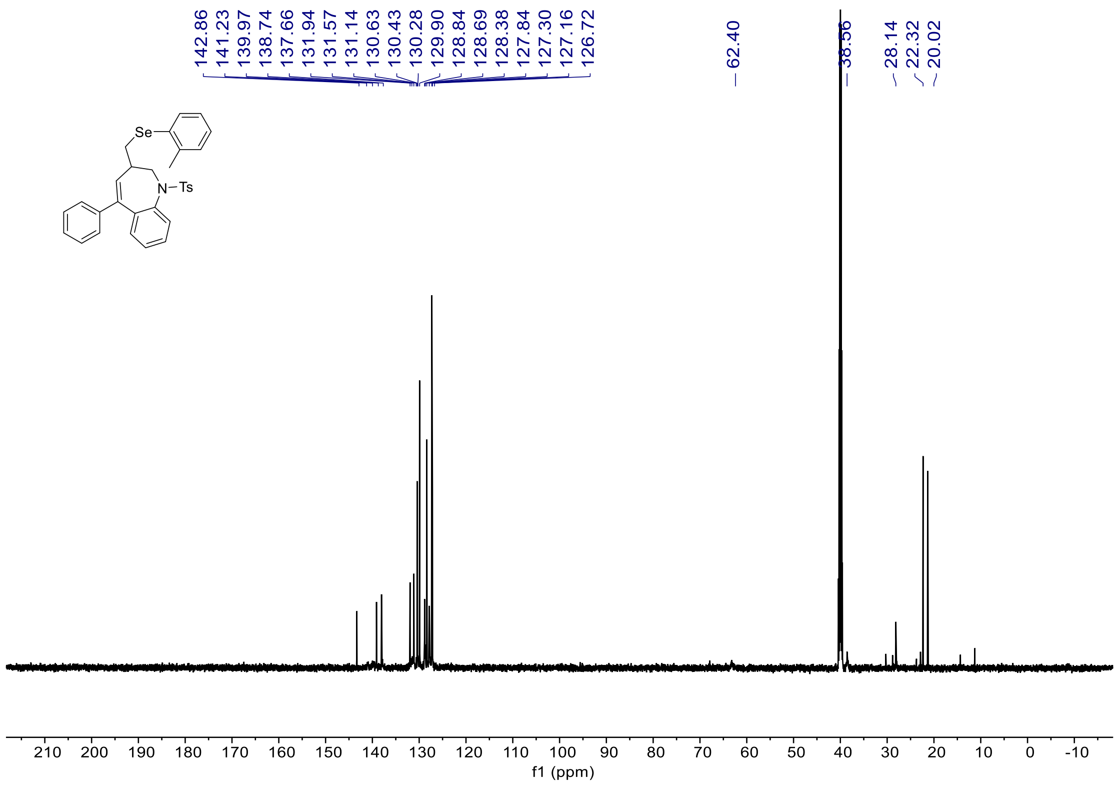

^{1}H NMR (400 MHz, DMSO-d_6) of compound **3z**

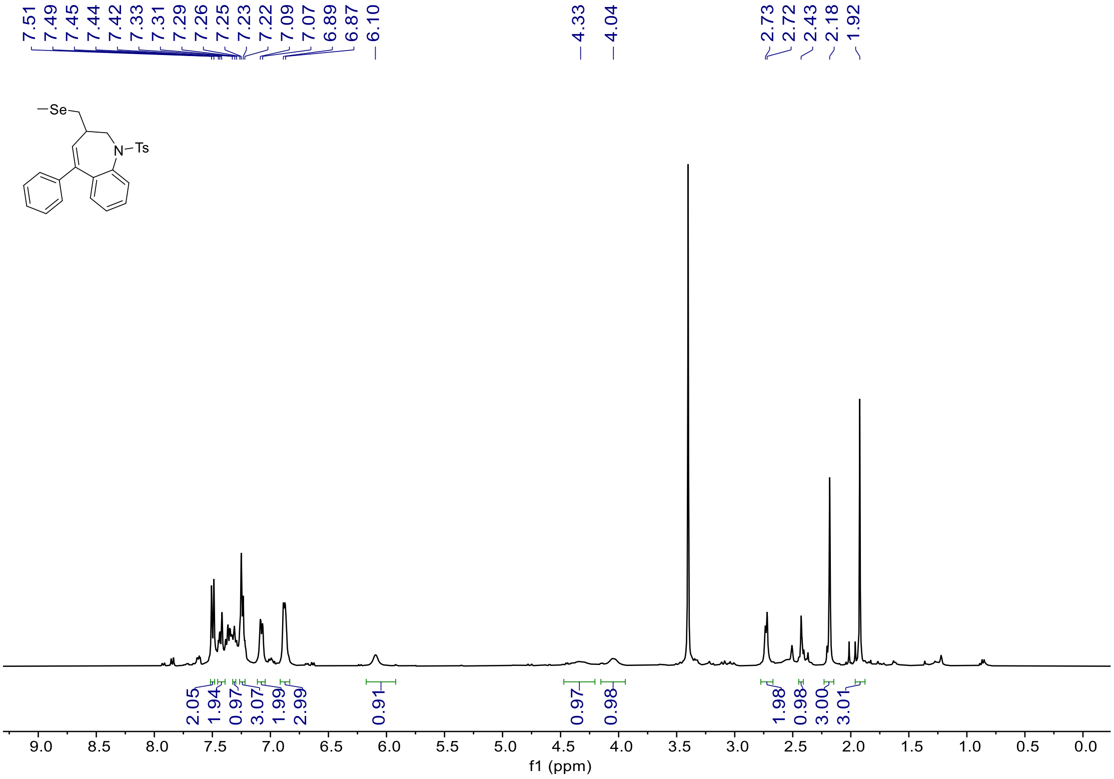

^{13}C NMR (101 MHz, DMSO-d_6) of compound **3z**

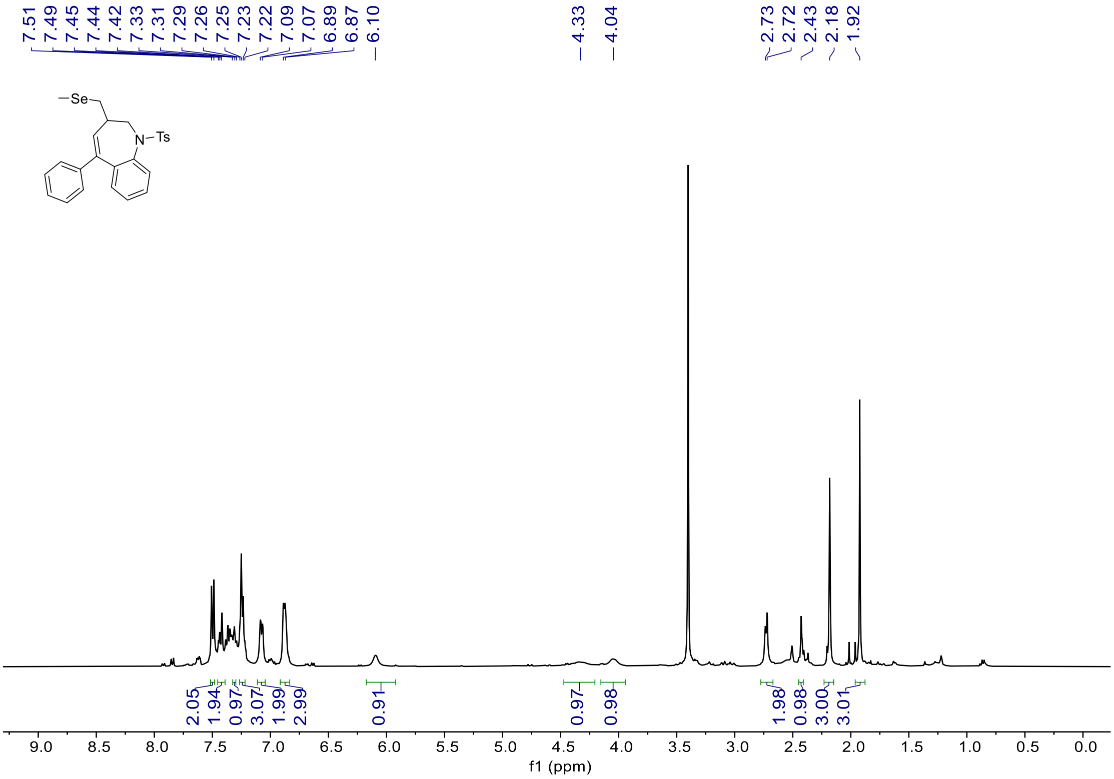

^{1}H NMR (600 MHz, DMSO-d_6) of compound **3za**

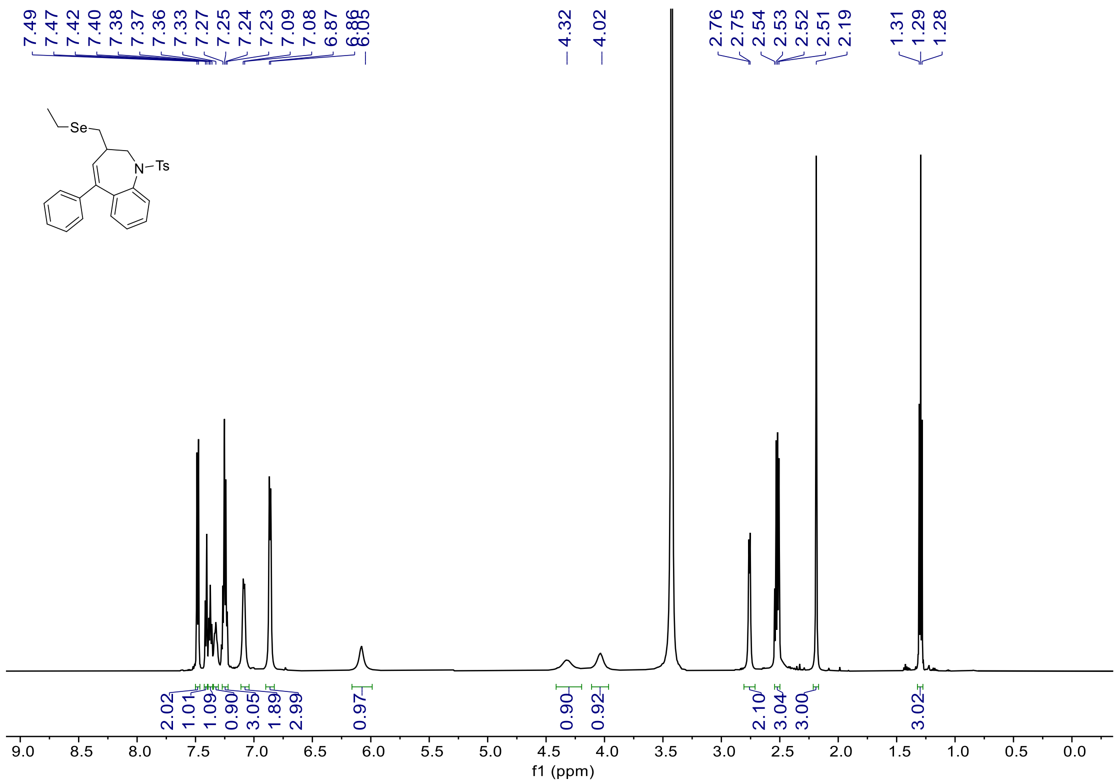

^{13}C NMR (151 MHz, DMSO-d_6) of compound **3za**

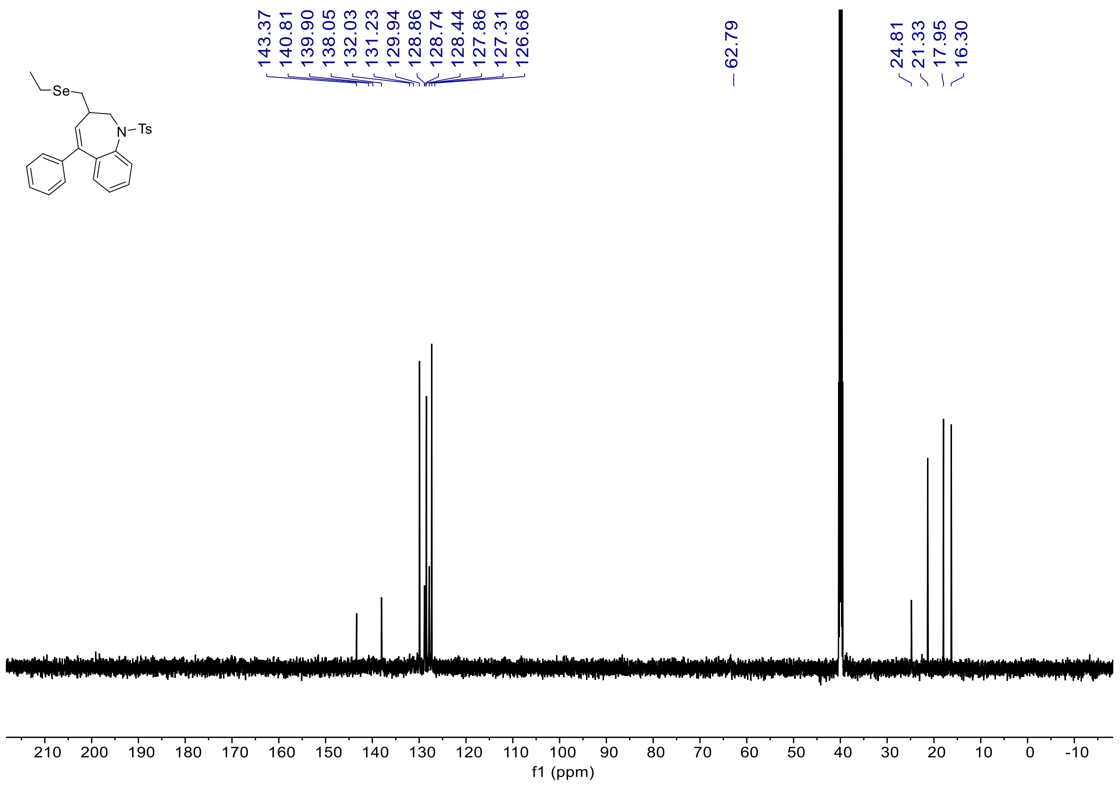

^{1}H NMR (600 MHz, DMSO-d_6) of compound **3zb**

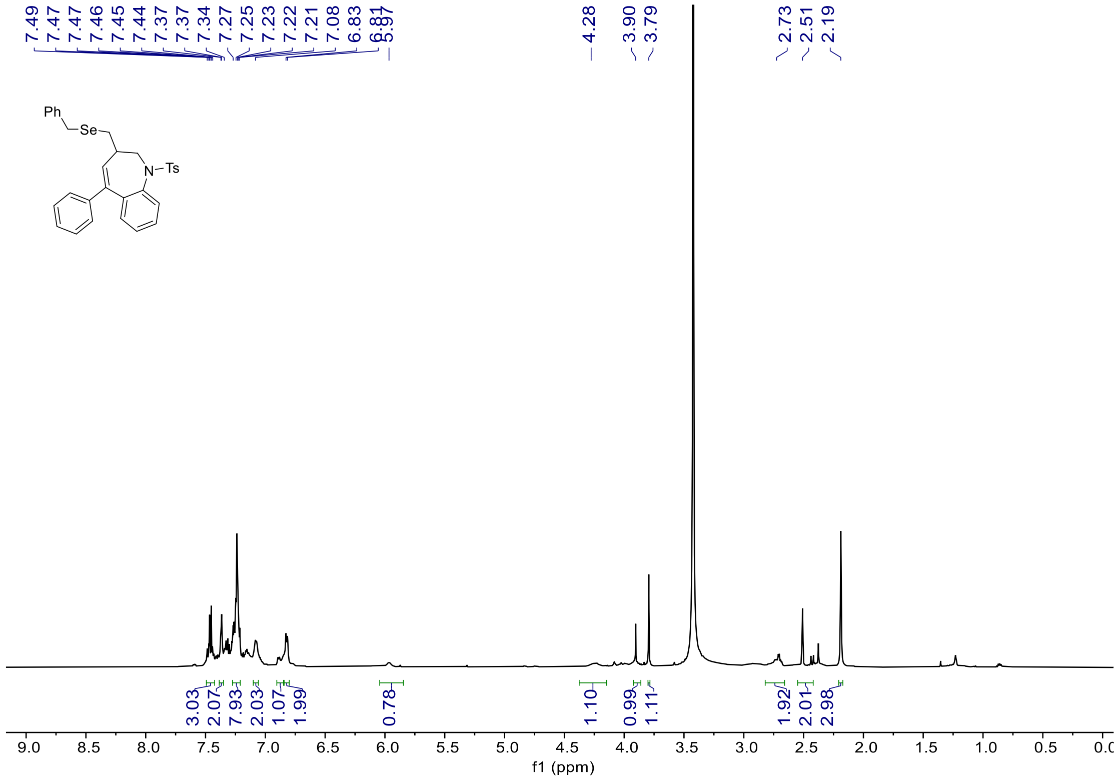

^{13}C NMR (151 MHz, DMSO-d_6) of compound **3zb**

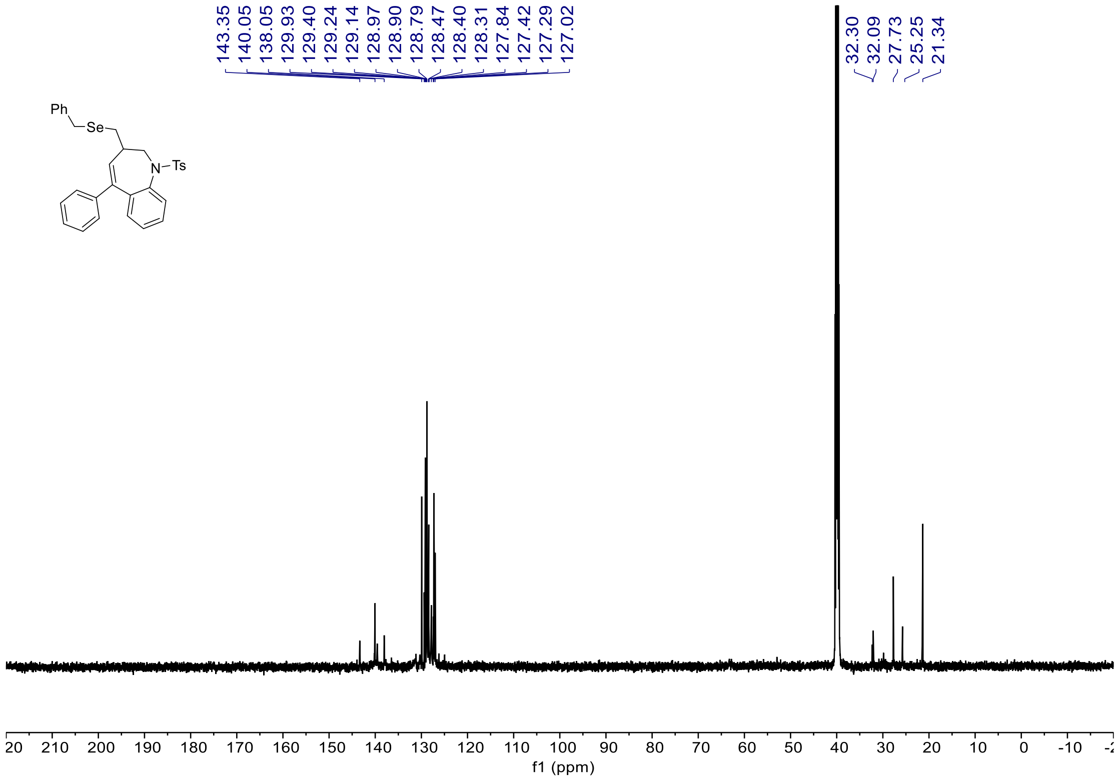

^{1}H NMR (600 MHz, DMSO-d_6) of compound **3zc**

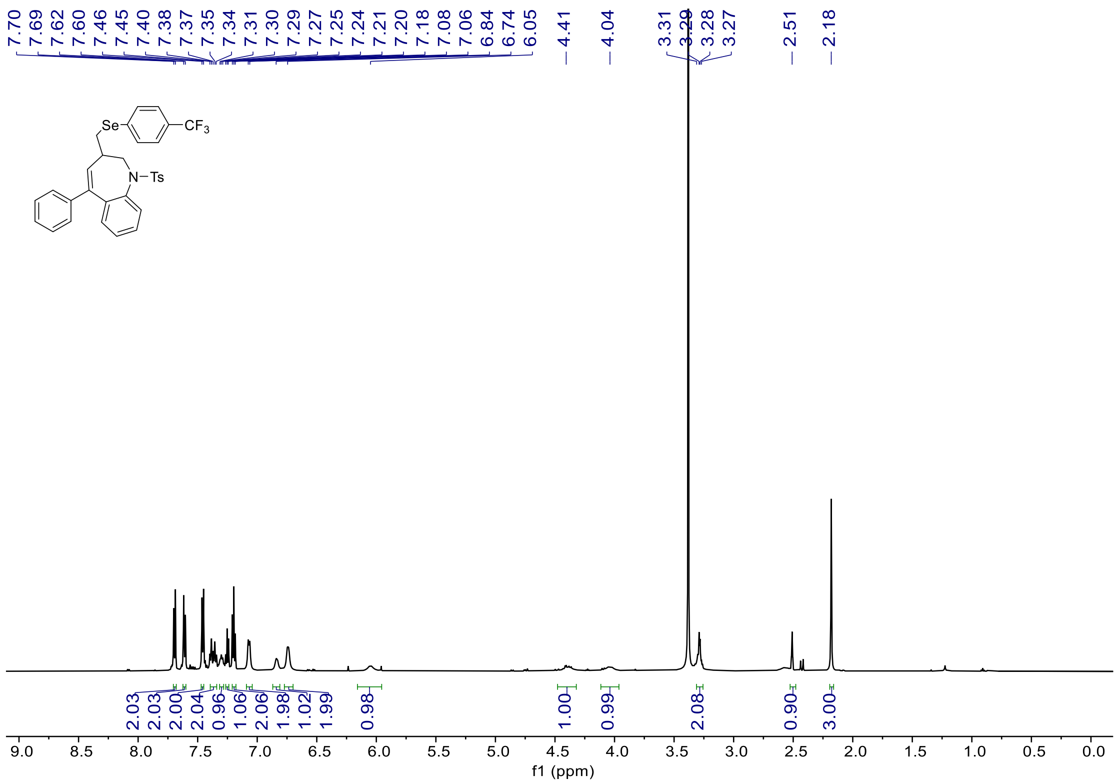

^{13}C NMR (151 MHz, DMSO-d_6) of compound **3zc**

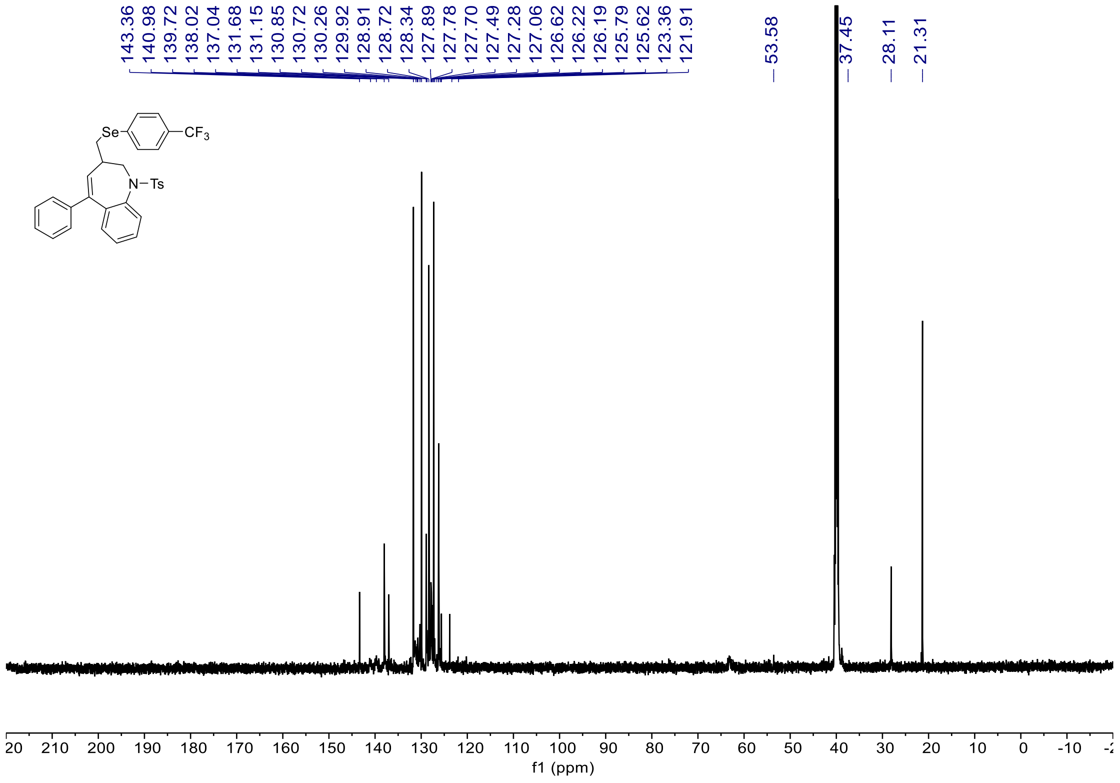

^{19}F NMR (565 MHz, DMSO-d_6) of compound **3zc**

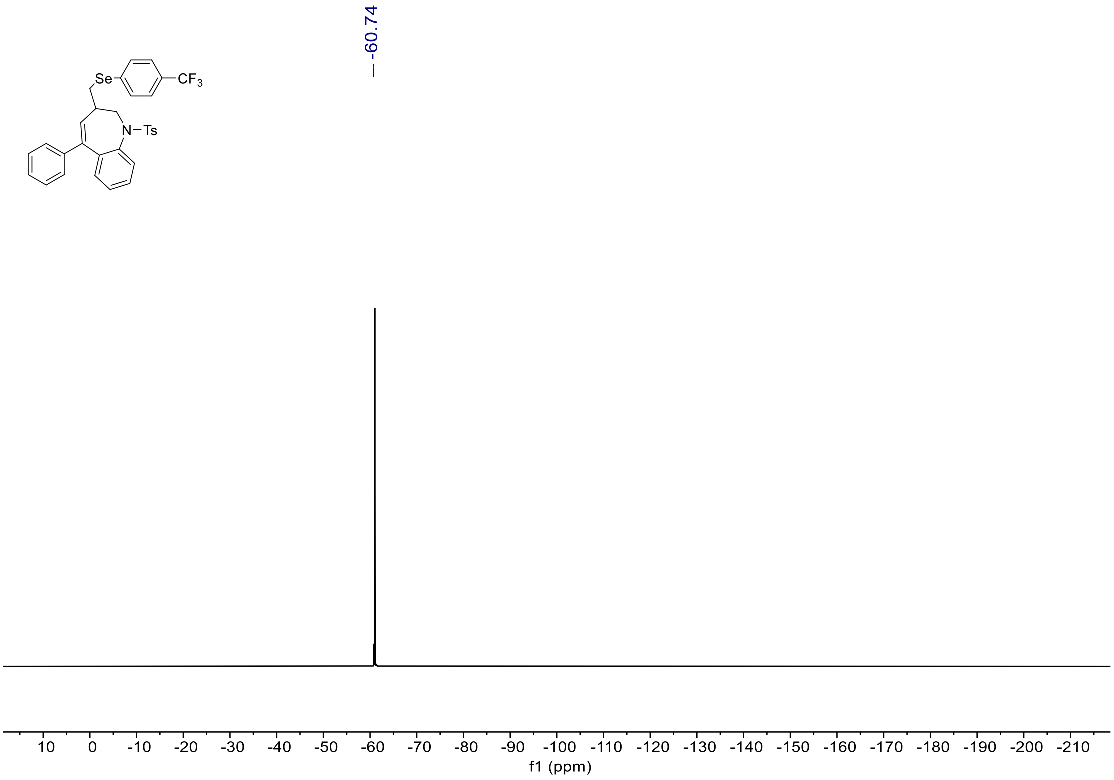

^{1}H NMR (600 MHz, DMSO-d_6) of compound **4a**

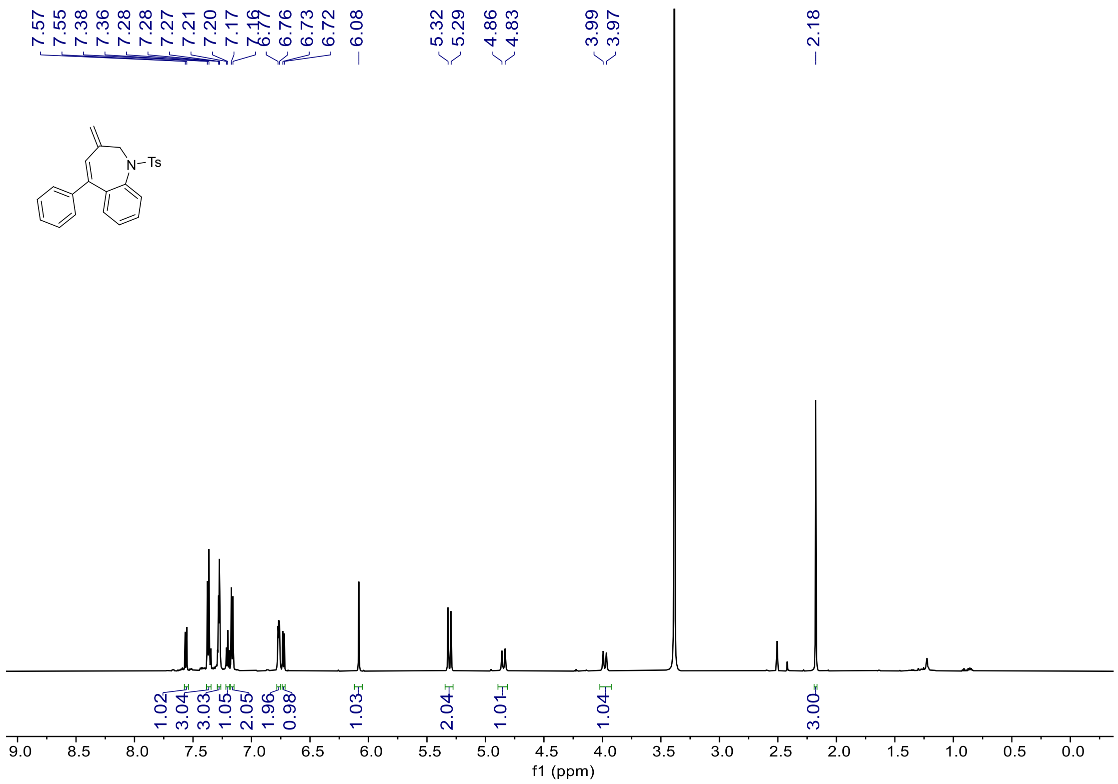

^{13}C NMR (151 MHz, DMSO-d_6) of compound **4a**

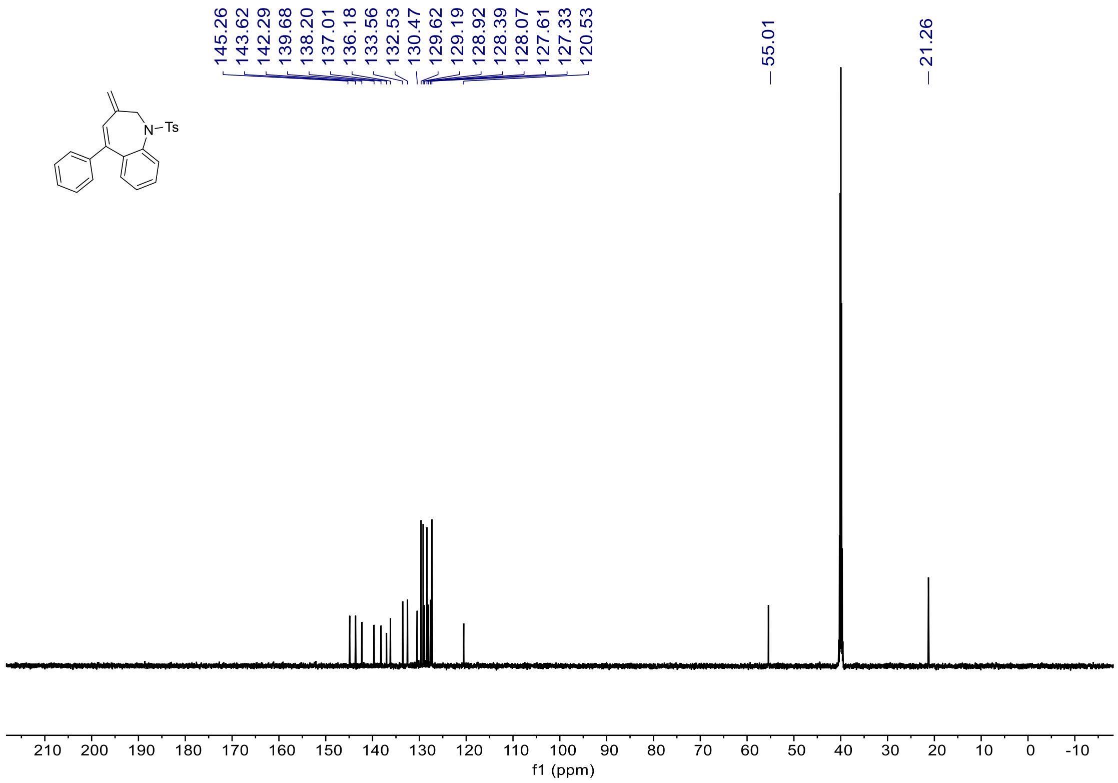

Supplement: RA-014-D4RA01914H-s001 [file RA-014-D4RA01914H-s001.pdf]
